# Fungal Hydrophobins: Taxonomic Distribution, Functional Roles, Physicochemical Properties, and Biotechnological Applications

**DOI:** 10.3390/jof12080587

**Published:** 2026-08-07

**Authors:** Sandra de Camargo Lameu, Matheus Henrique Galvão, Isabelle Teixeira Mello, Fabio Marcio Squina

**Affiliations:** Laboratory of Molecular Sciences, University of Sorocaba (UNISO), Rodovia Raposo Tavares, km 92.5, Sorocaba 18023-000, São Paulo, Brazil; drasandraclameu@gmail.com (S.d.C.L.); matheus.ebpb44@gmail.com (M.H.G.); isamello2002@gmail.com (I.T.M.)

**Keywords:** hydrophobins, filamentous fungi, physicochemical properties, amphipathic proteins, surface-active biomolecules

## Abstract

Hydrophobins are small, cysteine-rich amphipathic proteins predominantly produced by filamentous fungi, known for their ability to self-assemble at hydrophobic–hydrophilic interfaces. These proteins are essential for fungal development, surface interactions, pathogenicity, and environmental adaptation, and they have attracted growing interest for biotechnological applications. In this work, we provide a narrative review of fungal hydrophobins, based on a systematic literature search and manual curation of eligible studies integrated with protein database records from Ascomycota and Basidiomycota. Information was compiled from peer-reviewed publications selected according to predefined eligibility criteria and complemented with UniProt records, covering taxonomic distribution, functional and biophysical properties, and physiological and pathogenic roles. Significant diversity in molecular features and physicochemical profiles was observed, indicating functional specialization across different ecological niches and lifestyles. Additionally, the compiled data highlight various biotechnological applications, such as surface modification, enzyme immobilization, drug delivery systems, biomaterial development, and environmentally sustainable technologies. By consolidating molecular, functional, and applied information across a wide range of fungal species, this review provides a comprehensive reference framework for hydrophobin research and biotechnological innovation.

## 1. Introduction

**Figure 1:**
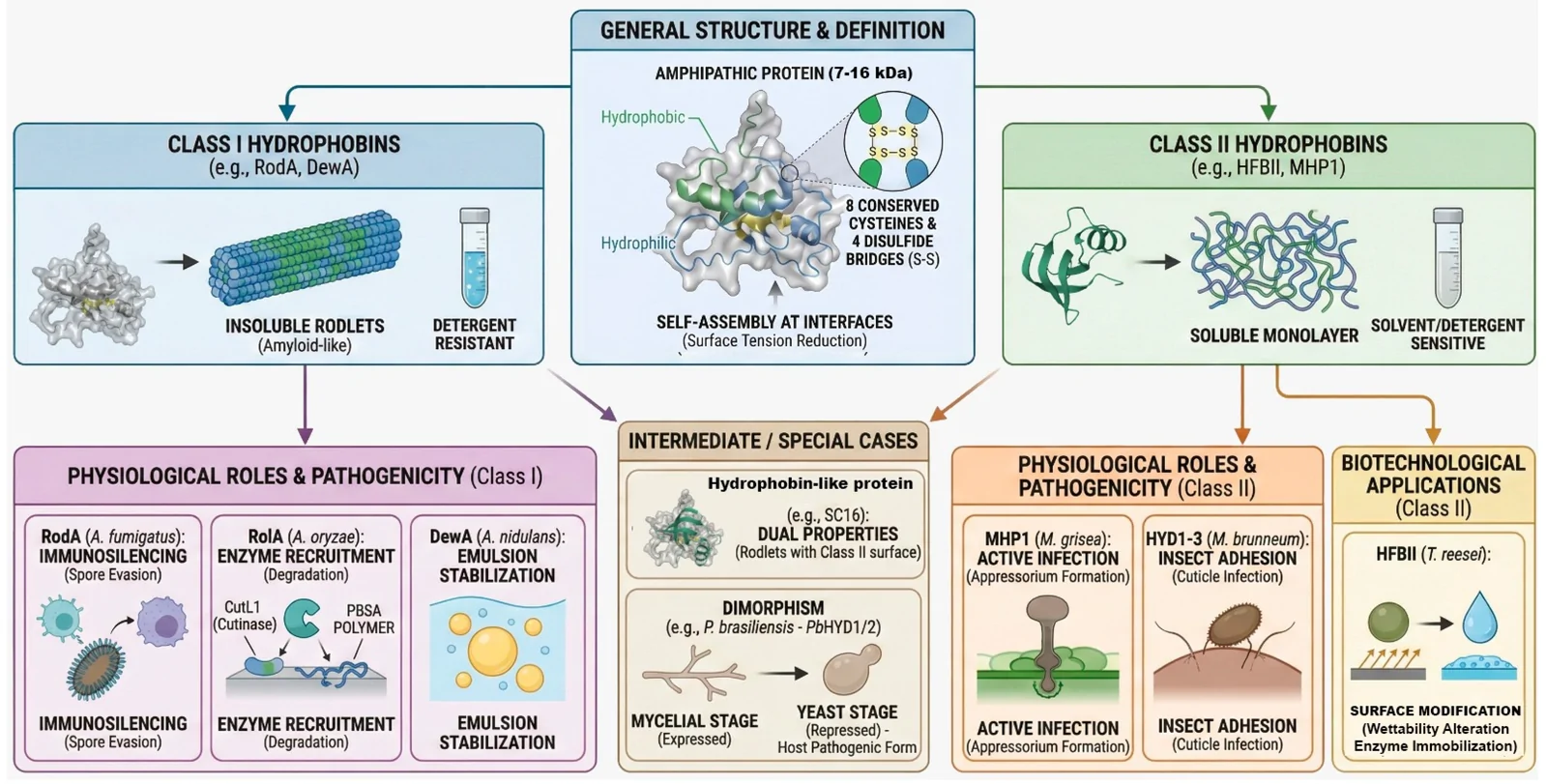
Main structural classification, functional diversity, and applications of fungal hydrophobins. Typically, the molecular mass of hydrophobins ranges from 7 to 16 kDa, but there are hydrophobin-like proteins with higher molecular masses, containing additional protein domains or extended sequence insertions (See Supplementary Tables S1 and S2).

Hydrophobins are small amphipathic proteins predominantly produced by fungi of the phyla Ascomycota and Basidiomycota, characterized by their ability to self-assemble at hydrophobic–hydrophilic interfaces, forming structured layers that alter surface properties. As surface-active molecules, they play essential roles in fungal growth and development, contributing to the formation of aerial hyphae, spore adhesion to surfaces, evasion of host immune responses, and interactions with the environment (Figure 1) [1].

Hydrophobin genes have also been identified in dimorphic fungi such as *Paracoccidioides brasiliensis* and *Ustilago maydis*, where their expression is predominantly associated with the filamentous growth phase rather than the yeast morphotype [2,3]. This observation reinforces the link between hydrophobin production and filamentous development and suggests that fungi restricted to yeast-form growth lack these proteins.

Typically ranging from 7 to 16 kDa and composed of approximately 70 to 130 amino acid residues, these proteins are stabilized by four conserved intramolecular disulfide bridges formed by eight cysteine residues. Many structurally characterized hydrophobins adopt a compact *β*-barrel-like fold, although structural variations occur among different classes and species. Despite their small size, they exhibit structural stability and surface activity, driven by the spatial segregation of hydrophobic and hydrophilic regions that define their amphipathic character. These molecular features underpin their ability to assemble at hydrophilic–hydrophobic interfaces, a property central to their roles in both natural and technological applications [1,4].

Based on structural and physicochemical properties, hydrophobins can be classified into two major groups. The Class I hydrophobins form highly stable, insoluble rodlet layers that resist chemical and thermal disruption, whereas Class II hydrophobins are more soluble, forming flexible monolayers at interfaces [1,5]. Both classes share the conserved cysteine pattern but differ in solubility and aggregation behavior [6].

Hydrophobins that cannot be assigned to either Class I or Class II represent a subset of proteins with atypical biophysical and self-assembly properties, but exhibiting cysteine patterns, disulfide bond topologies, and surface activity consistent with the hydrophobin functional paradigm. These unconventional hydrophobins exhibit variable aggregation behaviors that differ from the canonical rodlet layers typical of Class I and from the soluble, reversible films observed for Class II. Although some authors have proposed classifying it as Class III (or pseudo-Class I), this is not universally accepted. Their structural organization and surface assembly are often highly responsive to environmental conditions, including pH, ionic strength, and the presence of amphiphilic molecules such as lipids or polysaccharides [7].

The growing interest in these proteins reflects their multifaceted roles in fungal biology, development, and pathogenesis, as well as their biotechnological applications. Hydrophobins have been studied as stabilizers in emulsions, for coating nanoparticles, for immobilizing enzymes, and for improving the solubility and bioavailability of hydrophobic compounds [8,9]. Understanding the structural diversity and ecological functions of hydrophobins provides a foundation for expanding their use in medicine, materials science, and environmental biotechnology [10]. Therefore, a comprehensive and up-to-date review of fungal hydrophobins contributes not only to understanding their biological relevance but also to advancing the development of efficient and sustainable technologies.

## 2. Review Design

This study presents a curated narrative review based on a literature search, focusing on fungal hydrophobins and emphasizing their taxonomic distribution, functional roles, physicochemical characteristics, and biotechnological applications across the phyla Ascomycota and Basidiomycota. A literature search was conducted in PubMed, Web of Science, and Scopus, complemented by protein records retrieved from UniProt. The search included articles published up to February 2026 using combinations of the terms hydrophobin, fungal hydrophobin, Ascomycota, Basidiomycota, structure, function, and biotechnology.

Eligible studies included original research articles reporting (i) gene identification and annotation, (ii) expression evidence, (iii) biochemical characterization, (iv) structural and self-assembly properties, and/or (v) functional validation in vivo or in vitro. After screening titles, abstracts, and full texts for relevance, the references meeting these criteria were used as the primary source for selecting hydrophobin entries included in the compiled tables. Hydrophobin entries were subsequently curated and integrated with protein database information to generate the comparative datasets.

All data generated in this study consist of curated information derived from publicly available peer-reviewed publications and public protein databases (e.g., UniProt). The curated dataset is fully reported in the manuscript tables. Protein identifiers and accession numbers are provided whenever available to ensure reproducibility and traceability. For some hydrophobins, accession numbers could not be included because these proteins were identified only in the original publications and are not currently annotated in the UniProt database. For each hydrophobin, the following metadata were compiled, when available: UniProt entry, organism name, taxonomic placement, gene name and synonyms, reported functional and biophysical properties, experimental evidence described in the literature, and accession identifiers (including nucleotide accessions when reported in UniProt) (Supplementary Tables S1 and S2).

The curated protein sequences were subsequently used for sequence alignment and phylogenetic analyses to investigate the evolutionary relationships among fungal hydrophobins. Sequence alignments were performed using the MUSCLE algorithm [11] with default gap penalties (gap open penalty −2.90, gap extension penalty 0.00) and the UPGMA clustering method, implemented in the MEGA 12 software [12]. To minimize statistical noise generated by highly divergent terminal regions, a partial deletion approach was applied with a 95% site coverage cutoff. This strict filtering parameter resulted in final alignments comprising 45 conserved amino acid positions for the Ascomycetes dataset (191 sequences) and 89 positions for the Basidiomycetes dataset (115 sequences).

Evolutionary analyses were conducted using the Maximum Likelihood (ML) method based on the Jones-Taylor-Thornton (JTT) matrix-based substitution model [13] with uniform rates among sites. The initial trees for the heuristic search were generated automatically by applying Neighbor-Joining [14] and Maximum Parsimony (MP) algorithms to a matrix of pairwise distances computed using the p-distance model [15], subsequently selecting the topology with the superior log-likelihood value.

Statistical support for the clades was evaluated using an adaptive standard bootstrap test, which achieved convergence at 120 replicates for the Ascomycetes tree and 128 replicates for the Basidiomycetes tree. All phylogenetic computations were performed in MEGA 12 utilizing up to 7 parallel computing threads. The resulting original ML trees, conserving branch lengths and bootstrap support values, were exported in Newick format and graphically rendered using the Interactive Tree Of Life (iTOL) web platform [16]. The resulting phylogenetic analyses are presented in Supplementary Figures S1 and S2.

## 3. Hydrophobins in Ascomycota

The phylum Ascomycota comprises a genomically diverse group of fungi occupying a broad range of ecological niches, including saprotrophic, symbiotic, and pathogenic lifestyles [5,17,18,19,20,21,22,23,24,25,26,27,28,29,30,31,32,33,34,35,36,37,38,39,40,41,42]. Members of this phylum are also of considerable industrial importance due to their production of enzymes, antibiotics, and fermented products. Within Ascomycota, hydrophobins constitute one of the most extensively characterized protein families, displaying remarkable structural diversity and functional specialization associated with fungal development, environmental adaptation, host interactions, and biotechnological applications [43]. To organize and contextualize the available data, Table S1 compiles hydrophobins identified in Ascomycota, integrating taxonomic information with molecular and physicochemical features, including amino acid length, molecular weight, solubility, isoelectric point, GRAVY score, Pfam domain classification, subcellular localization, and accession identifiers. Also lists hydrophobins not deposited in the UniProt platform and includes proteins annotated as putative hydrophobins. Additionally, to complement the molecular and physicochemical dataset, Supplementary Figure S1 presents a phylogenetic analysis of Ascomycota hydrophobins, which are described throughout this review. Collectively, these data provide a comprehensive overview that emphasizes the broad functional diversity of Ascomycota hydrophobins, which are involved in processes such as fruiting body formation, hyphal organization, surface attachment, environmental resilience, and interactions with hosts.

### 3.1. Physiological Roles of Ascomycota Hydrophobins

The hydrophobins play essential roles in fungal morphogenesis and in modulating surface properties, serving as key determinants of the transition between submerged and aerial growth, substrate adhesion, and the stability of reproductive structures. A previous comparative genomic analysis of seven *Aspergillus* species identified 50 putative hydrophobins based on cysteine motifs, protein size, secretion signals, and hydropathy profiles, although most remain experimentally uncharacterized [44]. These data were subsequently integrated into functional genomics analyses, enabling a deeper understanding of regulatory and gene expression mechanisms within the genus [45].

In *A. nidulans*, six hydrophobin genes (RodA, DewA–E) have been identified and characterized. *RodA* is the main structural component of the conidial rodlet layer, responsible for hydrophobicity and resistance to environmental stresses, while *DewA*–*E* act in a complementary manner to protect against desiccation and ultraviolet radiation and to promote adhesion to substrates [46,47]. The RodA gene (AN8803), which encodes a 157-amino-acid protein, is transcriptionally regulated by BrlA and localized exclusively in the conidial wall, functioning synergistically with DewA to ensure spore hydrophobicity [48,49]. In vitro assays show that AnRodA and DewA can form fibrillar structures similar to those seen in vivo. Additionally, RodF and RodG display intermediate characteristics and cannot be classified as either Class I or Class II, which may be linked to spore adhesion and dispersal [43,50,51].

Hydrophobin structural conservation among *Aspergillus* species is evident in *A. fumigatus*, where the hydrophobin HYP1 can restore the rodlet phenotype in RodA-deficient strains. *A. fumigatus* also possesses at least seven hydrophobin genes (*RodA*–*RodG*), with *AfRodA* functionally similar to its homologues in *A. nidulans* [43,52]. In *A. oryzae*, the Class I hydrophobin RolA (HypA) forms Langmuir films and stable fibrillar assemblies through hydrophobic interactions mediated by Cys–Cys loops and zeta potential. This property confers amphipathic properties that support aerial hyphae formation, adhesion, and biofilm stability [4,46,51]. Another *A. oryzae* hydrophobin, HsbA, contributes to spore hydrophobicity and adhesion [43,53,54].

In *Neurospora crassa*, the Class I hydrophobin EAS is among the most extensively studied examples. Its ability to form rodlets and amphipathic layers confers resistance to spores and facilitates aerial dispersal, demonstrating remarkable stability under varying pH and temperature conditions [55,56].

The following table summarizes the physiological roles of hydrophobins from other Ascomycota members (Table 1):

### 3.2. Pathogenicity Roles of Ascomycota Hydrophobins

Comparative genomic analyses of *A. fumigatus*, *A. nidulans*, and *A. oryzae* have revealed substantial genetic diversity among these species, providing an evolutionary framework for understanding the diversification of hydrophobin functions and their adaptation to distinct ecological niches [102,103,104]. In *A. fumigatus*, hydrophobins form densely organized fibrillar structures that act as both physical and immunological barriers, masking epitopes recognized by the host immune system. Their expression is induced during sporulation, and deletion of the *RodA* gene results in conidia that lose hydrophobicity, exhibit increased permeability, and are more susceptible to antifungal agents, highlighting their protective function and importance in pathogenicity [7,105].

Δ*RodA* mutants in *A. fumigatus* exhibit structurally disorganized conidia that are more vulnerable to antifungal compounds and phagocytosis, confirming their role as an immune barrier [106]. The Class I hydrophobin RodB is expressed in biofilms and appears to participate in hyphal adhesion and cohesion, although it is not involved in rodlet formation in *A. fumigatus* [43]. The cysteine-rich proteins RodC, RodD, and RodE are also expressed during sporulation and likely play complementary roles in protecting and maintaining the cell wall, under conditions relevant to environmental adaptation and host interaction [7].

Functional diversity among hydrophobins in Ascomycota is further evidenced by the coexistence of multiple classes within the same organism. In *C. fulvum*, six hydrophobins (HCF-1 to HCF-6) have been identified, each with complementary functions and distinct structural distributions [72,73,107]. The first four belong to Class I and are located in conidia and aerial hyphae, whereas HCF-5 and HCF-6, both Class II, are associated with surface growth and adhesion during infection [108,109,110]. Among these, *C. fulvum* HCF-1 participates in conidial dispersal in aqueous environments, while HCF-6 is involved in adhesion and penetration into plant tissues; they contribute to the species’ pathogenicity. Constitutive expression of HCF-6 in mycelia, which decreases under carbon limitation, suggests that hydrophobin regulation depends on physiological and metabolic factors, reinforcing their adaptive character in mediating fungal–environment interactions [111].

In the genus *Trichoderma*, many species are mycoparasitic and widely used as biological control agents against plant pathogens. Species of *Trichoderma* possess one of the largest repertoires of hydrophobin-encoding genes among fungi, particularly Class II members [112], supporting roles in development, dispersal, and plant-associated interactions [85,113]. A Class I hydrophobin identified in *T. asperellum* is involved in root colonization and rhizosphere interaction [113], while hydrophobins such as HYTLO1 from *T. longibrachiatum* contribute to early plant–fungus signaling [114].

In the dermatophyte *Arthroderma benhamiae*, a causative agent of superficial mycoses in humans and animals [115], the hydrophobin HypA is the dominant cell-surface protein. HypA forms the rodlet layer that maintains conidial hydrophobicity and modulates host immune recognition. Deletion of HypA increases neutrophil activation and immune mediator release, indicating that this hydrophobin contributes to immune evasion during infection [116].

Among phytopathogenic fungi, the genus *F. harbours* multiple Class I and II hydrophobins. In *F. graminearum*, proteins such as FgHyd1–5 are regulated by G-protein complexes and the velvet system, participating in conidiogenesis, secondary-metabolite production, and host colonization [79]. In *F. culmorum*, the hydrophobin FcHyd3p facilitates adhesion to plant tissues and initiates infection [117].

In the genus *Cladosporium*, the species *C. fulvum* is an important biotrophic pathogen of tomato and a model for plant–microbe interactions. In the mango pathogen *Colletotrichum asianum*, hydrophobins contribute to early infection processes. The hydrophobin gene 14730 is involved in spore germination on hydrophobic plant surfaces and is regulated by CaGα1 signaling, suggesting a role in surface sensing and host penetration during infection [118].

In *Penicillium expansum*, the causal agent of blue mold in pome fruits, the hydrophobin HfbA is a major determinant of conidial surface hydrophobicity and promotes spore dispersal through both air and water. Deletion of *hfbA* alters conidial and conidiophore morphology, reducing dispersal efficiency and highlighting its essential role in fungal dissemination and environmental fitness [83].

In the *Magnaporthe* genus, hydrophobins play essential roles in morphological differentiation and plant infection. In *M. grisea*, the Class I hydrophobin MPG1 is required for appressorium formation, a penetration structure used to invade leaf tissues and that functions as a morphogenetic signal for surface recognition [119]. The Class II hydrophobin MHP1 is involved in conidial germination and the maintenance of virulence, and is expressed during infectious growth [81].

In entomopathogenic fungi such as *M. anisopliae* and *B. bassiana*, hydrophobins are directly linked to insect infection. In *M. anisopliae*, the Class I hydrophobin Hyd1 is a key mediator for cuticle adhesion, environmental stress resistance, and virulence, while Hyd2 and Hyd3 perform complementary functions [120,121]. In *B. bassiana*, Hyd1 and Hyd2 are structural components of the conidial wall and influence germination, conidiogenesis, and symbiotic interaction with plant roots [122,123].

Another entomopathogenic fungus, *Beauveria bassiana*, is used as a biological control agent against insect pests and disease vectors. Nine hydrophobins have been identified, including six Class I (Hyd1A–F) and three Class II proteins (Hyd2A–C). These proteins localize mainly on the cell walls of aerial hyphae and conidia, while accumulating in vacuoles and vesicles in submerged hyphae and blastospores, suggesting roles in surface hydrophobicity and developmental differentiation [68,69].

In the *Ophiostoma genus*, cerato-ulmin (CU), a Class II hydrophobin, is associated with Dutch elm disease. Although its toxic effects remain controversial, CU has been proposed to facilitate disease development by moving freely through the aqueous environment of the xylem in infected elm trees, where it may contribute to host damage. In addition, CU promotes the formation of hydrophobic biofilms at air–water interfaces, enhancing fungal growth and adhesion to plant tissues [124].

In *Verticillium dahliae*, a soilborne pathogen responsible for vascular wilt in numerous crops, the hydrophobin VdHP1 contributes to fungal morphogenesis and host interaction. VdHP1 influences surface hydrophobicity and can induce plant immune responses and cell death. It also participates in fungal adaptability and regulation of pathogenicity during plant colonization [125].

Among dimorphic fungi, *P. brasiliensis* expresses the Class I hydrophobins PbHyd1 and PbHyd2 during the mycelial phase. Both are related to the formation of aerial structures and adhesion of hyphae to hydrophobic surfaces, processes that are fundamental to the initiation of infection [126].

Extremophilic species such as *S. alkalinus* produce hydrophobins adapted to alkaline environments. The Class II hydrophobin Sa-HFB1 exhibits antifungal activity against opportunistic pathogens, including *C. neoformans*, suggesting a defensive role and potential pharmaceutical application for this protein [127]. In the following table, the pathogenicity-related roles of hydrophobins in Ascomycota members are summarized (Table 2):

### 3.3. Biotechnological Applications of Hydrophobins from Ascomycota

The industrial species *A. oryzae* produces the Class I hydrophobin RolA, which plays a unique role in polymer degradation and in the interface between biocatalysis and surface engineering. When cultivated in media containing the biodegradable polyester PBSA (polybutylene succinate-co-adipate), RolA is highly expressed and localized at the fungal cell wall, where it binds to hydrophobic surfaces and recruits the cutinase CutL1, catalyzing polymer hydrolysis. This cooperative mechanism between RolA and CutL1 exemplifies how hydrophobins can function not only as structural elements but also as mediators of enzymatic localization and interfacial catalysis [53].

Subsequent studies deepened the understanding of the RolA and CutL1 system. Specific ionic interactions between acidic residues, such as Asp142, Asp171, and Glu31, in CutL1, and basic residues, such as His31 and Lys34, in RolA that regulate enzymatic anchoring, are modulated by physicochemical parameters such as pH and ionic strength [4,150].

The ability of RolA to form organized films and alter surface properties also makes it relevant in biomedical and nanotechnological applications. Hydrophobin coatings enhance cellular uptake and the antitumor efficacy of curcumin nanoparticles without altering the drug-release profile [151]. Similarly, the functionalization of clay nanotubes with hydrophobins has yielded more efficient sustained-release systems for doxorubicin, improving dispersion and in vitro cytotoxicity [152].

In more complex therapeutic systems, the hydrophobin GLP-1 complexes extend the half-life of bioactive peptides by protecting them from enzymatic degradation, an effect associated with the high conformational stability of Class I hydrophobins, such as those from *A. fumigatus* [153]. The ability of these proteins to form stable amphipathic films and to modulate wettability (assessed by contact angle measurements) has been explored in the design of water-responsive surfaces and gas-exchange control devices [9]. In lipid-based systems, coating liposomes and niosomes with hydrophobins such as RodA from *A. fumigatus*, which represents a promising alternative to PEGylation, provides greater selectivity and biocompatibility to drug formulations such as doxorubicin, while reducing immune recognition [154].

On an applied scale, simple techniques such as dip-casting with RolA enable the stable anchoring of enzymes, antibodies, and other biomolecules on solid surfaces, creating robust platforms for biosensors, implant coatings, and diagnostic devices [155]. Comparative mapping of hydrophobins among *Aspergillus* species therefore provides a valuable set of structural targets for the rational design of coatings and nanosystems with predictable performance [43,44,45].

In *Trichoderma*, a genus widely exploited for its biotechnological potential, Class II hydrophobins predominate, including HFBI, HFBII, HFBIII, HFB4, and HFB7, which are involved in biocontrol, adhesion, and emulsion stabilization. HFBI and HFBII, in particular, display a high capacity for self-assembly at air–water interfaces and have been used for nanoparticle functionalization and recombinant protein purification systems [5,85,112].

Beyond their established biotechnological roles, hydrophobins have recently been implicated in environmental remediation, particularly in the removal of microplastics. Klauer et al. (2025) [156] demonstrate that hydrophobins mediate strong fungal–microplastic interactions, enabling efficient flocculation and particle removal from aqueous systems, even in the absence of fungal cells. These findings highlight the potential of surface-active hydrophobins, such as HFBI and HFBII, for engineered strategies to decontaminate microplastic pollution. In the following table, the biotechnological applications of hydrophobins in Ascomycota members are summarized (Table 3):

## 4. Hydrophobins in Basidiomycota

The phylum Basidiomycota comprises a diverse group of fungi, including mushrooms, rusts, smuts, and basidiomycetous yeasts. Sexual reproduction in this phylum is characterized by the formation of basidiospores on specialized structures called basidia, which in many species are organized into complex fruiting bodies known as basidiocarps [5]. Within Basidiomycota, hydrophobins contribute to fungal morphogenesis and ecological fitness by regulating processes such as fruiting body development, aerial hyphae formation, surface adhesion, and host interactions. Comprehensive genomic and biochemical studies have also highlighted their functional diversity and their importance in environmental adaptation and fungal colonization [6,43,173,174,175,176,177,178,179,180,181,182,183,184,185].

Hydrophobins characterized in Basidiomycota species display considerable diversity in structural features and physiological functions, reflecting their participation in complex developmental and ecological processes. To integrate UniProt data on characterized hydrophobins, Table S2 presents all the biophysical properties of hydrophobins in Basidiomycota and includes putative hydrophobins identified through sequence-based prediction from the UNIPROT database. Additionally, to complement the molecular and physicochemical dataset, Supplementary Figure S2 presents a phylogenetic analysis of Basidiomycota hydrophobins, which are described throughout this review.

### 4.1. Physiological Roles of Basidiomycota Hydrophobins

Basidiomycota hydrophobins allow the fungus to regulate surface interactions, facilitating the transition from submerged to aerial growth and providing structural integrity to reproductive tissues [5,186]. In *Schizophyllum commune*, the hydrophobin SC3 is among the most extensively studied examples. It self-assembles into highly ordered, fibrillar films at the air–water interface, promoting the formation of aerial hyphae and conferring hydrophobicity to the hyphal surface [187,188]. These assemblies also protect spores and hyphae against desiccation and environmental stress [1].

In *Agaricus bisporus*, several hydrophobins, such as ABH1, ABH2, ABH3, and HYPA, are expressed at specific stages of fruiting body development [189]. ABH1 forms hydrophobic rodlet layers in the cap tissues, protecting the fruiting body from mechanical and water stress, while ABH4 displays a unique self-assembly pattern that modulates surface tension at hydrophilic–hydrophobic interfaces [190]. Differential gene expression of HypA and hypB reveals functional specialization. HypA predominates in undifferentiated hyphae, whereas hypB is expressed during primordium differentiation [189,191].

In *Pleurotus ostreatus*, hydrophobins Vmh2, Vmh3, and Hydph16 contribute to cell wall formation, lignin degradation, and adaptation to stress conditions such as oxidative or detergent exposure [192,193,194]. The deletion of *Hydph16* results in thinner aerial hyphal walls, confirming its role in morphogenesis [194]. Some of these hydrophobins are light and temperature-inducible, mediating the formation of aerial mycelium and primordia in response to environmental triggers [195].

Fruiting body initiation and tissue differentiation in *Flammulina velutipes* are also tightly linked to hydrophobin expression. Genes such as *fvh1* and *Hyd9* are specifically expressed after induction of fruiting, controlling the formation of aerial hyphae knots and primordia. Their temporal regulation underscores the importance of hydrophobins as developmental markers in mushroom morphogenesis [196,197,198].

Similarly, *Lentinula edodes* produces multiple hydrophobins, including Le.hyd1 and Le.hyd2, whose expression patterns differ across developmental stages [199,200]. Le.hyd1 transcripts are abundant in primordia and pre-hymenial regions, while Le.hyd2 predominates in the dikaryotic mycelium. Electrical stimulation of the substrate increases hydrophobin secretion, linking these proteins to electrophysiological signaling during primordium induction [201,202].

In *Coprinopsis cinerea*, the hydrophobins CoH1 and CoH2 are developmentally regulated, with expression confined to monokaryotic vegetative hyphae [203]. Their absence in oid-1 mutants correlates with the loss of aerial growth, indicating a requirement for these proteins in fruiting initiation. Recent evidence suggests that regulatory miRNAs (cci-milR-12c, cci-milR-13e-5p) fine-tune hydrophobin expression during the transition from vegetative to reproductive stages [204].

Hydrophobins also contribute to environmental sensing and morphogenetic regulation in genera such as *Pholiota* and *Agrocybe*. In *P. nameko*, hydrophobin genes *pnh1*–*3* are induced under phosphate deficiency and electrical stimulation, linking environmental stress to morphogenesis [205]. In *A. cylindracea*, hydrophobins act as surface modifiers, altering the wettability of glass and Teflon and enabling the formation of aerial hyphae even in nutrient-rich conditions [206].

In symbiotic basidiomycetes like *Pisolithus tinctorius*, hydrophobins such as HydPt-1, HydPt-2, and HydPt-3 mediate hyphal adhesion and ectomycorrhiza formation. Their expression peaks during the early stages of plant–root interaction, assisting in hyphal aggregation and water exclusion at the symbiotic interface [207,208]. Finally, in *Tricholoma vaccinum*, hydrophobin Hyd8 is strongly induced during aerial mycelium formation and ectomycorrhizal symbiosis, where it contributes to surface structuring and compatible host recognition [209].

In the following table, the physiological roles of hydrophobins in Basidiomycota are summarized (Table 4):

### 4.2. Roles of Basidiomycota Hydrophobins in Fungal–Host Interactions

Hydrophobins in pathogenic and symbiotic Basidiomycota act as molecular interfaces between fungal cells and their environments, regulating adhesion, recognition, and immune evasion. In the phytopathogenic fungus *U. maydis*, which causes corn smut disease, the hydrophobins Hum2 and Hum3 were initially implicated in host adhesion and the formation of hydrophobic aerial hyphae. However, subsequent functional and genetic analyses demonstrated that these hydrophobins are dispensable for pathogenic development and are functionally replaced by repellents, a distinct family of secreted amphipathic proteins that assemble at the cell surface, reduce wettability, and ensure the maintenance of hydrophobicity during host colonization [239].

In ectomycorrhizal Basidiomycetous species that establish mutualistic or semi-pathogenic relationships with plants, also exploit hydrophobins to mediate attachment and colonization. In *P. tinctorius*, an ectomycorrhizal fungus forming symbiotic associations with tree roots, hydrophobins HydPt-1, HydPt-2, and HydPt-3 are transcriptionally upregulated during the early stages of root colonization [207,208]. Likewise, Hyd8 from *T. vaccinum* is highly expressed during ectomycorrhiza formation with *Picea abies*, where it participates in fungal surface organization and host recognition [209]. These proteins promote hyphal aggregation and facilitate adhesion to root epidermal cells, forming hydrophobic barriers that limit desiccation and microbial invasion while stabilizing the mycorrhizal sheath. Although primarily symbiotic, these interactions share functional parallels with pathogenesis, including controlled penetration and defense modulation at the host–fungus interface [242].

In *C. neoformans*, a pathogenic yeast that infects humans, hydrophobin-like proteins contribute to virulence and biofilm formation by providing a hydrophobic barrier that shields the fungal surface from immune detection [164].

Regarding pathogenicity roles in Basidiomycota, the hydrophobins Hah1 (Q3HS86) and Hah2 (Q3HS87) from *H. annosum* belong to Class I and are involved in the regulation of aerial mycelial development and environmental responsiveness to gas composition. HAH1 is expressed in aerial hyphae but is only weakly expressed in submerged mycelia and during seedling infection, with its transcription reduced under antagonistic interactions and low-oxygen conditions. HAH2 exhibits greater sensitivity to atmospheric composition, showing increased expression under ambient conditions and repression under oxygen-limited conditions [243,244].

Similarly, the hydrophobins Pgh1 (Q2VTP0) and Pgh2 (Q2VTN9) from *Phlebiopsis gigantea* belong to Class I and play roles in hyphal development and antagonistic interactions during wood colonization. Pgh1 is strongly expressed during early hyphal growth and in barrage zones, with late-stage upregulation supporting aerial hyphae formation, sporulation, and enhanced antagonistic performance. Pgh2 exhibits basal expression under nutrient-rich conditions, is induced during the early stages of confrontation, but is downregulated in barrage zones and at later stages. Together, these hydrophobins contribute to the biological control potential of *P. gigantea*, enabling it to outcompete *Heterobasidion* spp. on conifer stumps and thereby assisting in the management of pine root and butt rot caused by *H. annosum* [245,246].

### 4.3. Applications of Hydrophobins from Basidiomycota in Biotechnology

Hydrophobins from Basidiomycota have properties that make them candidates for surface functionalization, nanostructure design, and biomaterial engineering, bridging natural biological interfaces with technological applications [5]. The Class I hydrophobin SC3 from *S. commune* is a well-established model for biotechnological surface modification. Its amphipathic films, which self-assemble into stable rodlet layers, can be immobilized on metals, glass, polymers, and biological membranes [188]. The surface-modifying capacity of SC3 is reversible under controlled conditions, allowing reactivation and regeneration of biofunctional surfaces without structural degradation [6].

In environmental and materials biotechnology, ABH3 from *A. bisporus* has been used to create self-cleaning, anti-fouling, and moisture-regulating materials [247]. These hydrophobins can reduce surface energy and adjust wettability, serving as biological alternatives to fluorinated coatings in sustainable manufacturing. Their ability to immobilize enzymes such as laccases or cellulases further expands their use in bioremediation and biocatalysis, particularly in aqueous or multi-phase systems [190].

Hydrophobins from *P. ostreatus* (Vmh2 and Vmh3) also show promise in nanobiotechnological applications. Their ordered films exhibit distinct electrical and mechanical properties, making them suitable as biotemplates for nanowire formation and in biosensor design [193]. In the following table, the biotechnological applications of hydrophobins from Basidiomycota members are summarized (Table 5):

## 5. Conclusions

Altogether, hydrophobins from both Ascomycota and Basidiomycota represent an example of functional convergence between fungal biology and applied sciences. Across these phyla, hydrophobins play essential physiological roles in fungal growth and development, including the formation of aerial structures, spore dispersal, surface adhesion, and adaptation to environmental stresses. In addition, they are key determinants in fungal–host interactions, contributing to pathogenicity through mechanisms such as immune evasion, surface masking, host colonization, and infection structure formation.

Beyond their biological significance, the unique physicochemical properties of hydrophobins, particularly their ability to self-assemble at hydrophobic–hydrophilic interfaces, have positioned them as versatile molecules for biotechnological applications. These proteins have been successfully explored in areas such as surface functionalization, enzyme immobilization, drug delivery, biomaterial engineering, and environmentally sustainable technologies, including biocontrol and pollutant remediation.

Therefore, integrating structural, functional, and ecological knowledge across both fungal phyla highlights hydrophobins as multifunctional proteins with broad relevance. Continued investigation into their diversity and mechanisms of action will not only deepen our understanding of fungal biology but also expand their potential as natural building blocks for innovative and sustainable biotechnological solutions.

## Figures and Tables

**Table 1:** Physiological Roles of Ascomycota Hydrophobins.

Organism	Gene (Accession) and Class	Physiological Roles	References
*Aspergillus fumigatus*	RodA (P41746), RodB (E9QT94), RodC (Q4WBR8) and RodE (Q4WC31): Class I. RodD (Q4WE22), RodF (Q4WEK0) and RodG (Q4X055): hydrophobin-like protein.	Involvement on Surface/spore hydrophobicity, fungal growth and development, and environmental stress tolerance. *RodA* forms rodlets on the conidial surface. *RodB*-*G* do not participate in rodlet formation.	[7,57,58,59,60,61,62]
*Aspergillus nidulans*	DewA (P52750), DewB (Q5BC93), DewC (Q5AZ79), DewE (Q5AVZ1) and RodA (P28346): Class I. DewD (Q5BEU0): hydrophobin-like protein.	Involvement in surface/spore hydrophobicity and fungal dispersal. *DewD* and *DewE* are expressed in hyphae; *DewA*, *DewB* and *DewC* are mainly associated with conidial surfaces. *DewC* contributes to growth on lignocellulose. *RodA* is essential for the formation of hydrophobic rodlet layers on asexual spores and for conidial transfer. *RodA* indirectly enhances lignocellulose utilization by promoting close substrate–enzyme–fungus interactions.	[43,46,47,50,63,64,65,66,67]
*Beauveria bassiana*	Hyd1A (J4KPV6), Hyd1B (J4W2A8), Hyd 1C (J5JJC1), Hyd 1D (J4VS95), Hyd 1E (J4UGK4) and Hyd 1F (J4UJ50): Class I. Hyd 2A (J4WDT5), Hyd2B (J5JTB1) and Hyd2C (J4WMI6): Class II.	Involvement in cell wall hydrophobicity and fungal development. *Hyd1A* and *Hyd1B* form and coregulate the spore coat rodlet layer essential for conidial hydrophobicity, whereas *Hyd1C-F* and *Hyd2A*-*B* are not required for conidial hydrophobicity. *Hyd1B* participates in pre-penetration or surface conditioning processes, and *Hyd2C* contributes to asexual fungal development.	[68,69]
*Botryotinia fuckeliana*	Bhp1 (A0A384JA57), Bhp2 (A0A384JW35) and Bhp3 (A0A384JJM8): Class II.	*Bhp1* is required for maintaining conidial and hyphal hydrophobicity and for normal conidiation. *Bhp3* contributes to conidial morphology and chain formation. *Bhp2* shows no detectable role in hydrophobicity, growth or sporulation, suggesting a redundant or condition-specific function.	[70,71]
*Cladosporium fulvum*	Hcf-1 (Q00367), Hcf-2 (O94217), Hcf-3 (Q7Z9L5) and Hcf-4: Class I. Hcf-5 (O94203) and Hcf-6 (Q9C2X0): Class II.	Involvement in surface hydrophobicity, conidial development, germination and fungal adhesion. *HCF*-*1* forms part of the rodlet layer and conidial hydrophobicity and water-mediated spore dispersal. *HCF*-*2* cell surface hydrophobicity. *HCF*-*3* involved in maintenance of hyphal integrity in planta. *HCF*-*4* may assist adhesion within the apoplast. *HCF*-*5* is strongly upregulated during sporulation under nutrient-depleted conditions and shows stage- and environment-dependent regulation, whereas *HCF*-*6* is involved in fungal adhesion.	[72,73]
*Claviceps fusiformis*	TH1 (Q9UVI4): Class II.	Involvement in the reduction of water surface tension at the water–air interface and contribution to aerial hyphae formation.	[74]
*Cordyceps militaris*	CmHyd1 (A0A2H4SBS0) and CmHyd2 (A0A2H4SHM4): Class II. CmHyd3 (A0A2H4SKW2) and CmHyd4 (A0A2H4SI69): Class I.	Involvement in conidiation, aerial hyphae formation, stress tolerance and fruiting body development. *CmHYD1* contributes to osmotic, oxidative and thermal stress tolerance. *CmHYD4* negatively regulates aerial mycelial growth, carotenoid and adenosine synthesis and oxidant-stress resistance. *CmHYD2* and *CmHYD3* contribute to fruiting body development, and *CmHYD2* contributes to the formation of aerial hyphae.	[75,76,77]
*Fusarium graminearum*	FgHyd1-4 (N/A): Class I. FgHyd5 N/A): Class II.	Involvement in conidiation, secondary metabolism and modulation of aerial structure properties. *FgHyd1*, *FgHyd2* and *FgHyd4* are expressed in aerial and submerged structures. *FgHyd3* is exclusively expressed in aerial hyphae on solid surfaces. *FgHyd2* and *FgHyd3* contribute to hyphal penetration. *FgHyd5* specifically affects aerial mycelial hydrophobicity.	[78,79,80]
*Magnaporthe grisea*	Mhp1 (N/A): Class II.	Involvement in conidial hydrophobicity, conidiation, appressorium induction, plant colonization. and fungal development.	[81]
*Neurospora crassa*	Eas (Q04571): Class I.	Self-assembly into cross-β amyloid rodlets, promotion of spore formation and dispersal, and adaptation to environmental and circadian cues.	[55,82]
*Penicillium expansum*	HfbA (A0A0A2K7M3), HfbB (A0A0A2JSZ6), HfbC (A0A0A2IC94), HfbD (A0A0A2K253), HfbE (A0A0A2KI06) and HfbF (A0A0A2JK41): Class I. HfbG (A0A0A2J434): Class II.	Involvement in surface hydrophobicity, conidiophore development and spore dispersal. *HfbA* is uniquely expressed in spores and is essential for hydrophobicity, spore morphology and both air and water dispersal. *HfbB* is expressed during conidiophore development and influences hydrophobicity. *HfbC*–*HfbF* are expressed during hyphal growth. *HfbG* is expressed during hyphal growth.	[83]
*Trichoderma guizhouense*	Hfb2 (N/A), Hfb3 (A0A1T3CWR1), Hfb4 (A0A1T3C949) and Hfb10 (A0A5P8PB18): Class II.	Involvement in light-responsive regulation and modulation of spore-surface hydrophobicity during asexual development. All are induced by blue light in a BLR1 and ENV1-dependent manner. *hfb2* is moderately expressed during development. *hfb3* shows low basal expression and indirect light-mediated induction. *hfb4* is more expressed during development and plays a prominent role in spore-surface hydrophobicity. *hfb10* is the most expressed gene and uniquely depends on the HOG1 MAPK pathway in addition to BLR1 and ENV1, positioning it centrally in light-responsive hydrophobin production.	[84,85]
*Trichoderma harzianum*	Qid3 (P52755), Srh1 (P79072), Hfb2 (N/A), Hfb 2-a2 (N/A), Hfb3 (A0A2N1LQG4), Hfb4 (A0A2N1LTE2), Hfb7 (A0A0B5H3H0) and Hfb10 (A0A5P8PAX6): Class II or hydrophobin-like protein.	Involvement in plant interaction, adhesion, spore formation, stress response, and dispersal regulation. *Qid3* is induced by chitin and nutrient deprivation, influencing cytokinesis. *Srh1* is associated with sporulation. *HFB2* and *HFB3* exhibit low basal expression, suggesting condition-specific roles. *HFB2*-*a2* shows enhanced adsorption to hydrophobic surfaces. *HFB4* and *HFB10* regulate aerial spore dispersal. *HFB7* likely functions in oxidative-stress tolerance and environmental establishment.	[86,87,88,89,90,91,92,93]
*Trichoderma reesei*	hfb1 (P52754), hfb2 (P79073) and hfb3 (G0RVE5): Class II.	Involvement in hyphal development, aerial mycelium formation, sporulation and surface hydrophobicity. *hfb1* is central to aerial hyphae formation. *hfb2* is required for efficient sporulation. *Hfb3* contributes to vegetative growth and asexual development.	[94,95,96,97,98]
*Xanthoria ectaneoides*	XEH1 (Q9HFD2): Class I.	Involvement in rodlet formation and species delimitation, with pronounced sequence polymorphism among Mediterranean populations providing a molecular signature for distinguishing *Xanthoria* species.	[99,100]
*Xanthoria parietina*	XPH1 (Q9HFD1): Class I.	Involvement in rodlet-layer formation and maintenance of population cohesion, with high genetic conservation supporting its use as a marker for species boundary assessments.	[99,100,101]

**Table 2:** Pathogenicity Roles of Ascomycota Hydrophobins.

Organism	Gene (Accession) and Class	Pathogenicity Roles	References
*Arthroderma benhamiae*	HypA (D4ARW0): Class I.	Masks fungal cell wall components and prevents recognition by the host immune system, thereby influencing disease progression by reducing immune detection of pathogen-specific biopolymers.	[116]
*Aspergillus flavus*	-	Associated with corneal infection	[128]
*Aspergillus fumigatus*	RodA (P41746), RodB (E9QT94), RodC (Q4WBR8) and RodE (Q4WC31): Class I. RodD (Q4WE22): RodF (Q4WEK0) and RodG (Q4X055): hydrophobin-like protein.	*RodA* can mask immunogenic cell-wall components and prevents early Dectin-1 and Dectin-2 recognition, allowing germination prior to immune detection; its removal promotes recognition by dendritic cells and alveolar macrophages, highlighting *RodA* secretion as a potential antifungal target. *RodB*-*G* contribute to rodlet-layer integrity, and defects or alterations in these proteins increase fungal sensitivity to antifungal agents, supporting their relevance for the development of targeted antifungal therapies.	[7,57,58,59,60,61,62]
*Aspergillus nidulans*	RodA (P28346): Class I.	Provides immune evasion, while acting as a virulence factor by masking conidial recognition by the host receptors Dectin-1 and Dectin-2.	[57,59,60]
*Cladosporium herbarum*	HCh-1 (Q8NIN9): Class I.	Exhibits IgE-binding allergenic properties, and recombinant forms of this fungal allergen improve the sensitivity and specificity of fungal allergy diagnosis.	[129]
*Colletotrichum asianum*	Gene 14730 (A0A8H3W1T0): Class not informed	Proposed to mediate germination on hydrophobic surfaces, acting downstream of CaGα1-dependent GPCR signaling and enabling sensing of hydrophobic cues and initiating pathogenicity-related germination responses.	[118]
*Colletotrichum siamense*	CsHydr1 (A0A6G6VYL3): Class I.	Required for full virulence on rubber tree leaves.	[130]
*Cordyceps militaris*	CmHYD1 (A0A2H4SBS0): Class II.	Regulates conidiation and cuticle-bypassing infection by modulating frq and vosA transcription and by influencing primordium formation through MAPK signaling. It also contributes to osmotic, oxidative, and thermal stress tolerance, acts as a defensive barrier against *Calcarisporium cordycipiticola*, and controls AreA transcription via a developmental positive feedback loop.	[75,76]
*Cryphonectria parasitica*	Cryparin (P52753): Class II.	The most abundant protein produced in liquid culture and plays a key role in the fitness of this plant pathogen by enabling the eruption of fungal fruiting bodies through the host tree bark.	[131,132]
*Fusarium verticillioides*	Hyd1 (Q6YF32), Hyd 2 (Q6YF31) and Hyd 3 (Q6YF30): Class I. Hyd 4 (Q6YF29) and Hyd 5 (Q6YD93): Class II.	Microconidial chain formation, spore–surface interaction, and adhesion to hydrophobic epicuticles are compatible with insecticides. and insect-associated virulence. *HYD1* contributes to attachment to waxy epicuticles. *HYD2* supports proper chain architecture and adhesion to hydrophobic surfaces. *HYD3* participates in cuticle adhesion and supports epizootic spread in insect hosts. *HYD4* contributes to spore attachment to insect cuticles. *HYD5* regulates hydrophobic spore–epicuticle interactions and enhances insect-related virulence.	[133,134]
*Magnaporthe grisea*	MPG1 (P52751): Class I.	Highly expressed during early host infection and appressorium morphogenesis, with expression dependent on CPKA and NPR1 regulatory pathways.	[119,135,136]
*Metarhizium acridum*	HYD3 (E9DZB1): Class I.	It is located on the conidial surface and specifically activates humoral and cellular immunity in *Locusta migratoria manilensis*, enhancing resistance to fungal pathogens without affecting non-host insects.	[137]
*Metarhizium anisopliae*	ssgA (P52752): Class I.	Hydrophobic-surface sensing, infection structure differentiation and secretion of penetration enzymes during insect infection. It is induced by nutrient deprivation and late conidiation and is expressed across the pH range typical of insect cuticles.	[120,138,139]
*Metarhizium robertsii*	hyd1 (E9FDE9) and hyd2 (E9FBT2): Class I. Hyd3 (E9EN44): Class II.	Conidial hydrophobicity, adhesion to insect cuticle, sporulation, stress tolerance and pathogenicity. *Hyd1* is essential for insect virulence, with deletion causing reduced sporulation, increased hydrophilicity, impaired stress tolerance and disorganized rodlet bundles, although it has a negligible role in hyphal growth and asexual development. *Hyd2* and *Hyd3* show no significant role.	[121]
*Oidiodendron maius*	OmSSP1 (A0A0C3D4E3): Class I hydrophobin-like.	Symbiosis establishment and promotion of ericoid mycorrhiza formation, acting as an effector required for efficient colonization of *Vaccinium myrtillus* roots.	[140]
*Ophiostoma novo-ulmi*	Cerato-ulmin (CU) (Q06153): Class II.	Increases surface hydrophobicity and adhesion of yeast-like propagules, protects infectious cells against desiccation, and may induce Dutch elm disease–like wilt symptoms when expressed in nonpathogenic strains, functioning as a parasitic fitness factor whose contribution to virulence depends on the genetic background.	[141,142,143,144]
*Paracoccidioides brasiliensis*	Pbhyd1 (Q8NJQ1) and Pbhyd2 (Q6T935): Class I–like hydrophobins.	Regulation of morphological switching and early dimorphism-associated remodeling. Both are mycelium-specific and strongly induced during the early mycelium-to-yeast transition, suggesting involvement in growth and differentiation processes.	[126]
*Penicillium camembertii*	RodA (Q1I187): Class I.	ACTS in cell surface hydrophobicity, promotion of conidiation under aerial conditions and formation of a rodlet layer that masks airborne conidia from immune recognition.	[145,146]
*Pyricularia oryzae*	MPG1 (P52751): Class I. MHP1 (N/A): Class II.	Rodlet-layer assembly, hydrophobic-surface sensing, conidiation, pathogenicity and surface-associated enzyme retention. *MPG1* is highly induced during appressorium formation, plant infection and nutrient starvation, directing rodlet formation on conidia and mediating surface sensing for appressorium development. *MHP1* assembles into a non-fibrillar film and participates in retention of cutinase 2 at hydrophobic interfaces.	[119,135,147,148]
*Trichoderma virens*	TVHYDII1 (N/A): Class II.	Functions as an effector protein, promoting plant root colonization and antagonism against *Rhizoctonia solani*, likely aiding immune evasion, protection from plant defenses, and establishment of beneficial fungus–plant interactions.	[149]
*Verticillium dahliae*	VdHP1 (N/A): Class II.	Triggers plant cell death and immune activation. Acts as a negative regulator of virulence, modulating development, adaptability, and pathogenicity.	[125]

**Table 3:** Biotechnological Applications of Ascomycota Hydrophobins.

Organism	Gene (Accession) and Class	Biotechnological Applications	References
*Aspergillus nidulans*	DewA (P52750), DewC (Q5AZ79), DewE (Q5AVZ1) and RodA (P28346): Class I. DewD (Q5BEU0): hydrophobin-like protein.	*DewA* is a promising candidate for orthopedic implant coatings, promoting cell adhesion and tissue integration, and also functions as an emulsifier for biotechnological uses. *DewC*-*E* have been explored for surface modification, stabilization of emulsions and foams, enhancement of enzyme activity and antifouling strategies. The *RodA* rodlet layer contributes to immune evasion, biofilm formation and efficient lignocellulosic substrate deconstruction.	[43,46,47,50,63,64,65,66,67]
*Aspergillus oryzae*	RolA (Q2U3W7): Class I.	Overall roles include binding to hydrophobic polymer surfaces, recruitment of hydrolytic enzymes and enhancement of polymer degradation. *RolA* binds to polymers such as PBSA and PET, recruits and increases the activity of cutinases and PETases, and promotes polymer hydrolysis at the liquid–solid interface. Its interaction with PETase highlights its potential for eco-friendly PET recycling and for improving hydrolytic reactions on hydrophobic solid substrates.	[43,53,157]
*Beauveria bassiana*	Hyd1A (J4KPV6), Hyd1B (J4W2A8), Hyd 1C (J5JJC1), hyd1D (J4VS95), Hyd 1E (J4UGK4) and Hyd1F (J4UJ50): Class I. Hyd 2A (J4WDT5), Hyd 2B (J5JTB1) and Hyd2C (J4WMI6): Class II.	In entomopathogenic fungi such as *Beauveria bassiana*, these hydrophobins (*hyd1A-F*, *hyd2A-C*) contribute to traits associated with insect infection, supporting their potential use as biological alternatives to chemical pesticides due to the organism’s insecticidal activity.	[68,69]
*Clonostachys rosea*	Hyd1 (W8P1J0), Hyd2 (W8NXX4) and Hyd3 (W8NQ71): Class II.	Conidial hydrophobicity, biocontrol mechanisms and regulation of germination under stress conditions. *HYD1* is induced during conidiation and contributes to stress-mediated inhibition of germination. *HYD3* restricts premature germination under abiotic stress and is required for effective root colonization of *Arabidopsis thaliana*. *HYD2* shows constitutive basal expression, and its function remains unresolved, suggesting a potentially essential role.	[158]
*Fusarium culmorum*	FcHyd3p (N/A): Class I. FcHyd5p (N/A): Class II.	*FcHyd3p* exhibits weak foam-stabilizing activity and is not associated with gushing in beer. In contrast, *FcHyd5p* modifies surface hydrophobicity to increase wettability, stabilizes air bubbles in aqueous solutions, and acts as a potent gushing-inducing factor in carbonated beverages.	[117,159]
*Metarhizium brunneum*	Hyd1 (N/A) and Hyd 3 (N/A): Class I. Hyd 2 (N/A): Class II.	Sporulation, pigmentation, surface hydrophobicity and virulence. *HYD1* is strongly expressed in mycelia and conidiophores and weakly in conidia. *HYD2* is primarily expressed in blastospores. *HYD3* contributes to conidiogenesis and pathogenicity.	[160]
*Neurospora crassa*	NC2 (Q7S3P5): Class II.	Stabilizes air bubbles and forms ordered molecular networks on hydrophobic substrates, supporting applications such as biocompatible nanodevices and drug delivery systems.	[56,161]
*Paecilomyces farinosus*	PfaH1 (N/A); PfaH2 (N/A): Class I.	Modulation of surface hydrophobicity through increased water contact angles. *PfaH1* is abundantly produced in mycelium under diverse growth conditions, whereas *PfaH2* is constitutively expressed.	[162,163]
*Sodiomyces alkalinus*	Sa- Hfb1 (A0A3N2PQC3): Class II.	Isolated from the alkaliphilic fungus *Sodiomyces alkalinus* that exhibits antifungal activity, with MIC values ranging from 1 to 8 µg/mL against opportunistic and clinically relevant pathogens, including inhibition of *Cryptococcus neoformans*, suggesting a role in the organism’s antimicrobial defense and highlighting its potential as a promising antifungal lead compound.	[164]
*Trichoderma asperellum*	TasHyd1 (A0A6V8R0V1): Class I hydrophobin-like protein. Hfb 2-1 (A0A2T3ZKC8), Hfb 2-2 (A0A2T3ZC06), Hfb 2-3 (A0A2T3Z3L3), Hfb 2-4 (A0A2T3ZJV9), Hfb 2-5 (A0A2T3ZCF4), Hfb 2-6 (I7AQG9) and Hfb 2-7 (A0A2T3YYV2): Class II. Hfb2-8 (A0A2T3ZJ73): Hydrophobin-like gene lacking a signal peptide.	Root attachment and colonization, plant interaction, stress responsiveness and environmental sensing. *TasHyd1* is required for conidial wettability and colonization and is induced during plant interaction and carbon limitation. *HFB2*-*1* is selectively induced by woody plant extracts. *HFB2*-*5* and *HFB2*-*6* are strongly induced by plant-derived substrates and stress-related cues, whereas *HFB2*-*7* shows broader but lower-level induction. *HFBII*-*4* enhances plant defense. *HFB2*-*2* and *HFB2*-*3* are predicted to participate in stress-responsive and plant-associated processes. *HFB2*-*8* likely represents a nonfunctional pseudogene.	[113,165,166]
*Trichoderma atroviride*	Hfb-1b (N/A), Hfb-1c (N/A), Hfb-2a (N/A), Hfb-2b (N/A), Hfb-2c (N/A), Hfb-5a (N/A), Hfb-6a (N/A), Hfb-6b (N/A), and Hfb-6c (N/A): Class II; hfb-22a (N/A): Hydrophobin-like gene. Hfb4 (A0A7D5LZ09) and srh1 (N/A): hydrophobin-like protein.	Participate in light and stress-responsive regulation, surface interaction, and environmental adaptation. *hfb*-*1b*, *hfb*-*1c*, *hfb*-*2a*, *hfb*-*6a*, and *hfb*-*6b* are induced by BLR1/BLR2-dependent light signaling, whereas *hfb*-*2c* is BLR-independent and upregulated during early plant–host interaction. *hfb*-*2b* shows constitutive expression, including during host confrontation, while *hfb*-*6a* and *hfb*-*6b* are repressed during self-confrontation. The gene *hfb*-*22a* is likely a pseudogene due to lack of detectable expression. *HFB4* binds to diverse surfaces and enhances PET hydrolysis by cutinase, and *srh1* is predicted to contribute to surface interaction and environmental responsiveness.	[112,167,168,169]
*Trichoderma harzianum*	ThHyd1 (A0A219TTW8) and ThHyd2 (A0A1B1RVB9): Class II.	Involved in plant interaction, adhesion, spore formation, stress response, and dispersal regulation. *ThHyd1* acts as an elicitor of induced systemic resistance via brassinosteroid and JA/ET pathways, enhancing plant resistance without growth penalties, whereas *ThHyd2* shows limited ability to activate host defenses.	[86,87]
*Trichoderma longibrachiatum*	Hytlo1 (N/A): Class II.	Activation of plant defense signaling, induction of systemic resistance, antifungal activity and promotion of plant growth. It functions as a multifunctional MAMP that triggers rapid Ca^2+^ influx via a NAADP-dependent pathway, localizes as a surface film on plant cell walls, and is required for full antagonistic activity and plant-growth promotion during Trichoderma–plant interactions.	[114,170]
*Trichoderma reesei*	Hfb1 (P52754): Class II.	Surface modification and biotechnological applications, showing strong potential for technical and medical uses, particularly in the development of implant coatings.	[94,95,96,97,98]
*Trichoderma virens*	Hfb9a (N/A) and Hfb9b (N/A): Class I.	Plant root colonization, antagonistic activity and interfacial self-assembly. *HFB9a* and *HFB9b* self-assemble into ordered monolayers that modify surface wettability, and in combination with EPL1 form hybrid interfacial layers with altered wetting properties distinct from pure hydrophobin films.	[171,172]

**Table 4:** Physiological Roles of Basidiomycota Hydrophobins.

Organism	Gene (Accession) and Class	Physiological Roles	References
*Agaricus bisporus*	HypA (P49072), HypB (O43122) and HypC (O13300): Class I.	Fruiting-body development, tissue differentiation and surface adhesion. *HypA* accumulates during cap expansion and self-assembles into rodlets that generate protective hydrophobic coatings on the cap, veil and stipe. *hypC* is enriched in fruit bodies, particularly at the pin stage. *hypB* is highly expressed during early fruiting-body differentiation, especially in developing cap tissues.	[186,189,190,210]
*Agrocybe aegerita*	Aa-Pri2 (Q9Y8F0): Class I.	Fruiting-body development and developmental regulation. *Aa-Pri2* encodes a secreted protein specifically expressed during basidiocarp differentiation, with promoter architecture indicating tight developmental control and conserved homologs across several basidiomycete species, supporting a role in reproductive structure formation.	[211]
*Coprinopsis cinerea*	CoH1 (P78601) and CoH2 (P78602): Class I.	Aerial mycelial growth and light-regulated developmental transitions during fruiting-body formation. *CoH1* and *CoH2* are predominantly expressed in monokaryotic mycelia and contribute to aerial hyphal growth.	[203]
*Dictyonema glabratum*	Dgh1 (Q8WZJ3), Dgh2 (Q8WZJ2) and Dgh3 (Q8WZJ1): Class I.	Formation of hydrophobic hyphal wall coatings, maintenance of gas-filled interhyphal spaces, water translocation, and structural organization of the fruiting body. *DGH1* forms rodlet layers that maintain hydrophobic cavities within the photobiont layer and support tissue stratification. *DGH2* produces similar hydrophobic coatings in both photobiont and lower strata, contributing to basidiocarp structural integrity. *DGH3* shows developmentally regulated expression and participates in hydrophobic wall formation and compartmentalization in mature fruiting-body tissues.	[212,213]
*Flammulina velutipes*	fvh1 (Q9HDD5), Hyd-1 (Q75T84), Hyd-2 (A0A1I9QLC3), Hyd-3 (A0A1I9QLC4), Hyd-4 (A0A1I9QLC5), Hyd-5 (A0A1I9QLC6), Hyd-6 (A0A1I9QLC7), Hyd-7 (A0A1I9QLC8), Hyd-8 (A0A1I9QLC9), Hyd-9 (A0A1I9QLD0) and Hyd-10 (A0A1I9QLD1): Class I.	Regulation of fruiting-body development, hyphal behavioral transition, and structural differentiation during mushroom morphogenesis. *Fvh1* is strongly expressed immediately after fruiting induction in substrate-associated mycelia. *Hyd-1* is light-induced and highly expressed from the primordial stage through mature fruiting bodies. *Hyd-2-10* show low expression in vegetative mycelia and reduced transcription in fruiting bodies, suggesting specialized functions restricted to early or tissue-specific stages of morphogenesis.	[198,214]
*Laccaria bicolor*	LbH1 (B0CX61), LbH5 (B0DG11), LbH6 (N/A), LbH11 (B0E003) and LbH14 (B0E2E5): Class I.	Overall roles include fruiting-body development and host-dependent regulation. *LbH1* is induced specifically in Douglas fir–associated fruiting bodies, *LbH5* and *LbH14* are upregulated in fruiting bodies from both Douglas fir and poplar, *LbH6* is repressed during fruiting-body formation, and *LbH11* shows host-specific regulation, being repressed in Douglas fir but induced in poplar fruiting bodies.	[215]
*Lentinula edodes*	Le.hyd1 (Q9P8T0) and Le.hyd2 (Q9P8S9): Class I.	Fruiting-body development, mating-type–dependent regulation, and stage-specific hyphal growth. *Le*.*hyd1* is transcribed during the primordial stage and functions primarily in fruit body initiation. *Le*.*hyd2* is expressed in dikaryotic mycelia.	[199,201]
*Paxillus involutus*	hydA (Q4G2I6), hydB (A9X617), hydC (A9X618), hydD (A9X619), hydE (A9X668), hydF (A9X669) and hydG (A9X622): Class I.	Coordinated developmental regulation and differential transcriptional specialization during fungal growth. *hydA*-*C* and *hydE* show similar expression profiles, indicating shared regulatory control within the dominant hydrophobin module, with *hydE* displaying relatively stronger induction. *hydD* and *hydF* exhibit lower expression and distinct regulatory patterns, suggesting functional divergence and increased tissue specificity. *hydG* likely represents an allelic variant of *hydD*.	[216]
*Pholiota nameko*	pnh1 (Q8X1T9) and pnh2 (Q8X1T8); pnh3 (pnh3): Class I.	Adaptive responses to inorganic phosphate (Pi) deficiency, extracellular protein secretion and nutrient acquisition from wood substrates. *PNH1* is upregulated under Pi limitation. *PNH2* is induced during Pi deficiency. *PNH3* shows expression patterns similar to *PNH2*, being expressed under low Pi.	[205,217]
*Pisolithus tinctorius*	hydPt-1 (P52748), hydPt-2 (P52749) and hydPt-3 (O93917): Class I.	Early ectomycorrhizal development, root colonization, and aerial hypha formation. *HYDPt-1* accumulates on hyphal cell walls and is abundant across ectomycorrhizal tissues. *HYDPt-2* shows elevated expression in early eucalypt symbiotic tissues, is abundant in aerial hyphae, and follows a regulatory pattern similar to *HYDPt-1* and *HYDPt-3*. *HYDPt-3* is upregulated during ectomycorrhiza formation, co-regulated with the other two hydrophobins.	[208,218,219,220]
*Pleurotus ostreatus*	vmh1 (Q9Y7G9), Vmh2 (A0A067N4H9), Vmh3 (A0A067N648), Poh1 (O13502), Poh2 (O13503), Poh3 (O60048), fbh1 (O60047), Hydph6 (A0A067PCX8), Hydph7 (A0A067P1E6), Hydph8 (A0A067NFU6), Hydph12 (A0A067N4P3), Hydph13 (A0A067P494), Hydph15 (A0A067NHW8), Hydph16 (A0A067P943), Hydph17 (A0A067NHE1), Hydph18 (A0A067NEJ5), Hydph19 (A0A067NRJ8), Hydph20 (A0A067N7P3), Po.hyd1 (N/A), Po.hyd2 (N/A), Po.hyd3 (N/A), Po.hyd4 (N/A), Po.hyd5 (N/A), Po.hyd7 (N/A), Po.hyd8 (N/A), Po.hyd9 (N/A), Po.hyd10 (N/A), Po.hyd16 (N/A) and Po.Hyd1–10: Class I. Hydph5 (A0A067N582), Hydph9 (A0A067N4P7), Hydph11 (A0A067P0I9) and Hydph14 (A0A067NRQ5): hydrophobin-like protein.	Vegetative development, fruiting-body differentiation, surface structuring, aerial mycelium formation and environmental adaptation. *Vmh1* and *Vmh2* are restricted to vegetative mycelium, supporting surface interaction and stable coating formation, with *Vmh2* additionally enabling protein immobilization, biosensor applications and nanomaterial functionalization. *Vmh3* is glycosylated and expressed in both vegetative and reproductive stages, contributing to structural development. *POH1* and *fbh1* are fruiting-body–specific hydrophobins involved in rodlet formation and tissue specialization, whereas *POH2* and *POH3* function mainly during vegetative growth and extracellular surface assembly. *Hydph6-8*, *Hydph15*, *Hydph17* and *Hydph20* support vegetative growth, substrate colonization and aerial hypha formation, while *Hydph16* is essential for proper cell wall architecture and resistance to wall-targeting stress. Intermediate hydrophobins (*Hydph5*, *Hydph9*, *Hydph11* and *Hydph14*) contribute to developmental regulation and structural surface modulation. *Hydph12*, *Hydph13* and several *Po.Hyd* proteins (*Po.Hyd1–5*, *Po.hyd8*) are expressed during primordia and young fruiting bodies, supporting primordium initiation, fruit-body differentiation and pileus air-channel formation, whereas *Po.hyd7*, *Po.Hyd9* and *Po.hyd10* are associated with substrate colonization, dikaryotic growth and surface adaptation. *Po.hyd16* shows monokaryon-specific expression linked to early developmental specialization.	[194,195,221,222,223,224,225,226,227,228,229,230,231,232]
*Schizophyllum commune*	SC1 (P04158), SC4 (P16934), SC6 (O74300), SC16 (D8QCG9), Hyd3 (D8PTH5), Hyd4 (D8PU87) and Hyd6 (D8Q897): Class I.	Aerial hypha development, fruiting-body differentiation, hydrophobic surface structuring and biointerface formation through amphipathic self-assembly. *SC1* is a weakly expressed dikaryon-specific hydrophobin likely involved in auxiliary wall structuring and surface coating formation. *SC4* accumulates in fruiting-body hyphae, lining gas channels with hydrophobic membranes that maintain gas exchange and prevent water infiltration. *SC6* likely acts as a secondary structural component in fruiting tissues. *SC16* exhibits increased conformational flexibility due to the absence of one disulfide bond, enabling aeration-dependent rodlet formation and controlled self-assembly. *Hyd3* shows stable expression independent of thn1 regulation, *Hyd4* displays moderate repression with limited regulatory responsiveness, and *Hyd6* is strongly thn1-dependent.	[233,234,235,236]
*Suillus luteus*	HydSl1 (A0A0C9ZLX1), HydSl3 (A0A0D0AYW4), HydSl4 (A0A0D0BGG3), HydSl5 (A0A0D0AM44), HydSl6 (A0A0D0B5N3), HydSl7 (A0A0D0ALE0), HydSl8 (A0A0D0AMA6), HydSl9 (A0A0C9ZII0) and HydSl10 (A0A0D0AZF5): Class I.	Surface attachment, interface adaptation, adhesion, and structural coating functions.	[237]
*Tricholoma terreum*	Hyd1 (Q8TFP0): Class I.	Coating vegetative mycelium and aerial hyphae, forming the dominant surface layer in ectomycorrhizal tissues (mantle and Hartig net), contributing to hyphal hydrophobicity, supporting interface architecture for nutrient exchange, and displaying host-dependent regulation indicative of a role in symbiotic recognition and specificity.	[238]
*Ustilago maydis*	Hum2 (A0A0D1BZL3) and Hum3 (A0A0D1CKI0): Class I.	Aerial hyphae formation, surface hydrophobicity, and early cell-wall–associated morphogenesis. *Hum2* is strongly expressed during dikaryotic filamentous growth and acts as a key determinant of aerial hyphae, while *Hum3*, a chimeric hydrophobin–Rep1 fusion, plays a minor or redundant role, contributing to surface hydrophobicity and supporting redundancy with other wall-modifying proteins.	[239,240,241]

**Table 5:** Biotechnological Applications of Basidiomycota Hydrophobins.

Organism	Gene (Accession) and Class	Biotechnological Applications	References
*Agaricus bisporus*	ABH3 (Q9HGX0): Class I.	Expressed exclusively in vegetative mycelium; Substrate attachment, aerial hyphae emergence, and adhesion to hydrophobic surfaces. *ABH3* has potential biotechnological applications in enhancing adhesion and hydration of lignocellulosic materials, improving processes for lignin biodegradation and bioconversion.	[248]
*Coprinopsis cinerea*	Hyd1 (A8P9W4), Hyd2 (A8PA37) and Hyd3 (D6RQ66): Class I.	*Hyd1* is required for hyphal knot and primordium formation and is directly regulated by CcNsdD2, showing strong light-dependent induction. *Hyd2* is upregulated during early light-induced developmental initiation, while *Hyd3* is activated under light/dark rhythms and depends on CcNsdD2 for expression during early reproductive development, thereby enhancing yields in the edible mushroom industry.	[249]
*Ganoderma lucidum*	Hyd1 (A0A9E8S4N7): Class I.	Overall roles include primordium formation, developmental regulation, and stress tolerance. *Hyd1* is expressed during primordium initiation, and its silencing prevents primordium formation, demonstrating an essential role in early development. Its expression is negatively regulated by AreA, increases under nitrate conditions, and contributes to tolerance against heat, cell-wall, and salt stresses.	[250]
*Grifola frondosa*	HgfI (A4LAR6) and HgfII (A0A8A5N547): Class I.	Surface modification, self-assembly at interfaces, and biotechnological applications. *HGFI* forms interfacial nanolayers enabling antibody immobilization, enhances PET hydrolysis, supports antibacterial surface formation via nisin adsorption, and functions as an adhesive amphiphilic modifier promoting hyphal growth. *HGFII* is expressed in mycelia, self-assembles into rodlet-like nanostructures with increased β-sheet content, and exhibits stronger surface hydrophobicity and superior emulsifying capacity, enabling the formation of highly stable oil-in-water emulsions.	[152,251,252,253,254,255]
*Pleurotus ostreatus*	Vmh1 (Q9Y7G9), Vmh2 (A0A067N4H9), Vmh3 (A0A067N648), Fbh1 (O60047): Class I.	*Vmh1-3* and *fbh1* overall roles include surface immobilization, biocatalysis, wood degradation, and biotechnological applications. *Vmh1*, *Vmh2* and *Vmh3* support lipase immobilization via interfacial activation, with *Vmh2* additionally enabling GST-based biosensors for pesticide detection and chimeric antimicrobial surfaces when fused to LL-37. *fbh1* contributes to wood and lignin-derived compound degradation and supports production of secondary metabolites with pharmaceutical potential.	[221,222,223,224,225,226,227,228,229,230,231,232]
*Schizophyllum commune*	SC3 (P16933): Class I.	Forms highly insoluble rodlet layers that reduce surface tension and mediate adhesion to hydrophobic and hydrophilic interfaces, supporting stable surface coatings and biomaterial functionalization.	[233,234,256,257,258,259,260]
*Serpula lacrymans*	Slh1 (N/A) and Slh4 (F8NJA2): hydrophobin-like protein.	Rodlet formation, β-sheet assembly, and interfacial coating. *SL1* can be overexpressed in *E. coli*, refolded into an active conformation, and forms rodlets with strong thioflavin-T fluorescence, suitable for functional and biophysical assays. *SLH4* assembles efficiently at air–water interfaces, forming small, densely clustered rodlets with pH- and ionic-strength–dependent conformational changes, supporting controlled interfacial coatings and nanostructure formation.	[261,262]

## Data Availability

Data will be made available on request.

## Supplementary Materials

The following supporting information can be downloaded at: https://www.mdpi.com/article/10.3390/jof12080587/s1, Methodology for Sequence Alignment and Phylogenetic Analysis. Supplementary Figure S1. Maximum Likelihood phylogenetic tree of Ascomycota hydrophobins. Supplementary Figure S2. Maximum Likelihood phylogenetic tree of Basidiomycota hydrophobins. Supplementary Table S1. Biophysical Properties of Hydrophobins from Ascomycota. Supplementary Table S2. Biophysical Properties of Hydrophobins from Basidiomycota.

## Abbreviations

The following abbreviations are used in this manuscript:

GLP-1	Glucagon-like peptide-1
GPCR	G protein–coupled receptor
GRAVY	Grand Average of Hydropathy
IgE	Immunoglobulin E
kDa	kilodalton
MAPK	Mitogen-Activated Protein Kinase
PBSA	Poly(butylene succinate-co-adipate)
PEG	Polyethylene glycol
PET	Poly(ethylene terephthalate)
Pfam	Protein families database
pH	potential of hydrogen
UniProt	Universal Protein Resource
