# Supplementary material for: Fungal Hydrophobins: Taxonomic Distribution, Functional Roles, Physicochemical Properties, and Biotechnological Applications
Source: J Fungi (Basel). 2026 Aug 7;12(8):587. doi: 10.3390/jof12080587 (PMC13514795; doi:10.3390/jof12080587)
Supplement: Supplementary file 1 [file jof-12-00587-s001.zip › jof-4443196 - Supplementary Figures and Tables.pdf]

Journal of Fungi

MDPI

Supplementary Material

Fungal Hydrophobins: taxonomic distribution, functional roles, physicochemical properties, and biotechnological applications

LAMEU, Sandra de Camargo1, GALVÃO, Matheus Henrique2, MELLO, Isabelle Teixeira2 and SQUINA, Fabio Marcio1,*

1 Laboratory of Molecular Sciences, University of Sorocaba (UNISO), Rodovia Raposo Tavares, km 92.5, Sorocaba, São Paulo, 18023-000, Brazil.

Contents

- Methodology for Sequence Alignment and Phylogenetic Analysis (page 2)
- Supplementary Figure S1. Maximum Likelihood phylogenetic tree of Ascomycota hydrophobins (page 3).
- Supplementary Figure S2. Maximum Likelihood phylogenetic tree of Basidiomycota hydro-phobins (page 4).
- Supplementary Table S1. Biophysical Properties of Hydrophobins from Ascomycota (page 5).
- Supplementary Table S2. Biophysical Properties of Hydrophobins from Basidiomycota (page 34)
- References (page 49)

Methodology for Sequence Alignment and Phylogenetic Analysis

Protein sequences of fungal hydrophobins were aligned using the MUSCLE algorithm [1] (gap open penalty 2.90, gap extension penalty 0.00, UPGMA clustering method) implemented in MEGA 12 software [2]. To minimize statistical noise generated by highly divergent terminal regions, a partial deletion approach was applied with a 95% site coverage cutoff. This strict filtering parameter resulted in final alignments comprising 45 conserved amino acid positions for the Ascomycetes dataset (191 sequences) and 89 positions for the Basidiomycetes dataset (115 sequences).

Evolutionary analyses were conducted using the Maximum Likelihood (ML) method based on the Jones-Taylor-Thornton (JTT) matrix-based substitution model [3] with uniform rates among sites. The initial trees for the heuristic search were generated automatically by applying Neighbor-Joining (NJ) [4] and Maximum Parsimony (MP) algorithms to a matrix of pairwise distances computed using the p-distance model [5], subsequently selecting the topology with the superior log-likelihood was selected.

Statistical support for the clades was evaluated using an adaptive standard bootstrap test, which achieved convergence at 120 replicates for the Ascomycetes tree and 128 replicates for the Basidiomycetes tree. All phylogenetic computations were performed in MEGA 12 [2] utilizing up to 7 parallel computing threads. The resulting original ML trees, conserving branch lengths and bootstrap support values, were exported in Newick format and graphically rendered using the Interactive Tree Of Life (iTOL) web platform [6].

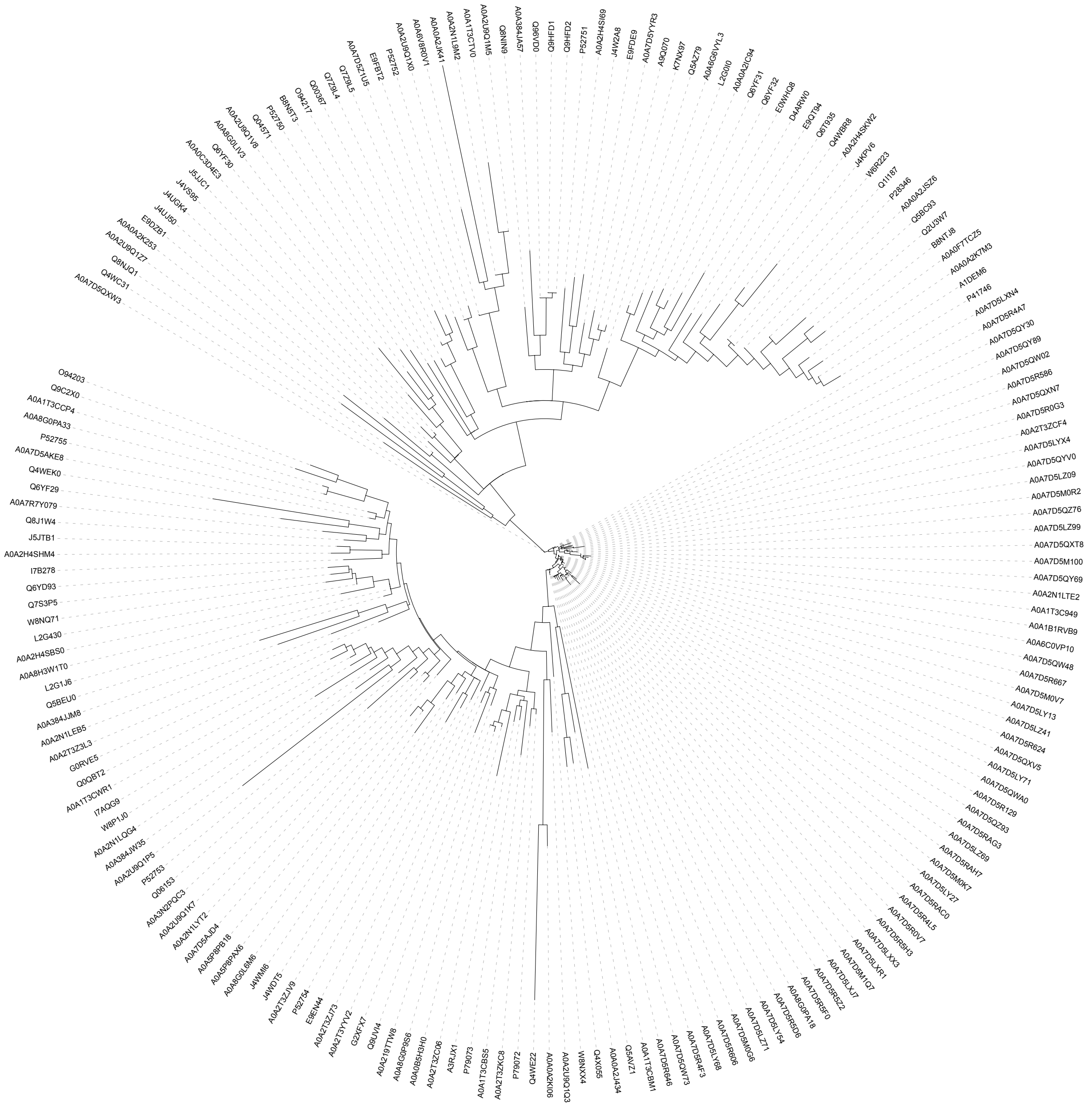

Figure S1. Maximum Likelihood phylogenetic tree of Ascomycota hydrophobins. The evolutionary history was inferred from 191 amino acid sequences using the Maximum Likelihood method and the Jones-Taylor-Thornton (JTT) matrix-based model. The final dataset comprised 45 conserved positions after applying a 95% site coverage cutoff (partial deletion) to remove highly divergent terminal regions. Branch lengths are proportional to the number of substitutions per site. Statistical support for the nodes was obtained using an adaptive bootstrap test with 120 replicates. Evolutionary analyses were conducted in MEGA 12 and visualized using iTOL.

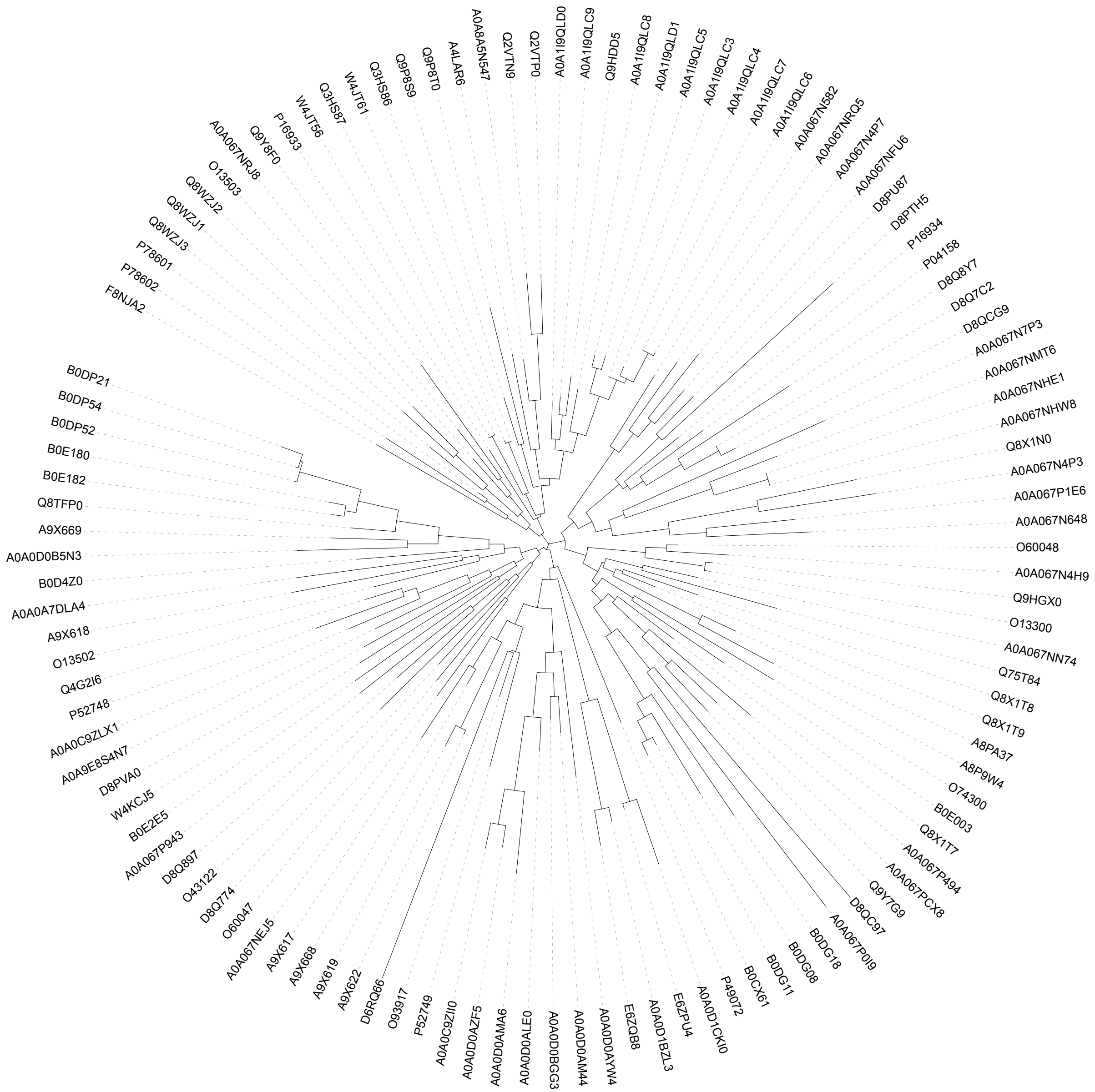

Figure S2. Maximum Likelihood phylogenetic tree of Basidiomycota hydrophobins. The evolutionary history was inferred from 115 amino acid sequences using the Maximum Likelihood method and the Jones-Taylor-Thornton (JTT) matrix-based model. The final dataset comprised 89 conserved positions after applying a 95% site coverage cutoff (partial deletion) to remove highly divergent terminal regions. Branch lengths are proportional to the number of substitutions per site. Statistical support for the nodes was obtained using an adaptive bootstrap test with 128 replicates. Evolutionary analyses were conducted in MEGA 12 and visualized using iTOL.

Journal of *Fungi*

Table S1. Biophysical Properties of Hydrophobins from Ascomycota. Mass (Da), solubility, isoelectric point (pI), and GRAVY index were predicted using ExPASy ProtParam (https://web.expasy.org/protparam/). Protein solubility was evaluated using Protein-Sol (https://protein-sol.manchester.ac.uk/). Subcellular localization was based on UniProt annotations; for those without this description (D), it was used DeepLoc (https://services.healthtech.dtu.dk/services/DeepLoc-2.0/).

Entry (Uniprot)	Organism	Gene Name	Pfam	Length	Protein Sequence	Mass (Da)	Solubility	Pi	Gravy	Location	Protein ID
**D4ARW0**	*Arthroderma benhamiae*	*HYPA*	PF01185	139	MKFSIAAAVLALASAVVAHPGAGYVSTPEKEA NFQQNFQKFVTACGNGNQVSCCNTETKKV GAPLTAGGLIPILDNINLEDFSLLKGCSKVDVA AVIGVQDLLNSNCKTQVSCCKVGDTNQVGL VNANVDLKCAAQNII	14.4	0.1	7.0	0.3	Secreted, cell wall	EFE34024.1 [115]
**B8NTJ8**	*Aspergillus flavus*	*rodA*	-	217	MQFSVAAVLALATAVAALPPASGTGAGQQVG HSKNDFPLPKELTTKQAADKCGDQAQLTCC NKTVKTGDFTQVEEGLLAGLLSNLLGAGQGS QGLGLLDECTNIPVIPIIPIASPQEQCKQPISC CQNTKSSAVSRINAQYTQLSLTITGWRPRRY WSSLHRSRLSPVSDCIREIGSSRSTGWKMA MKCFHSVLFCVLYWLLVSWNPRSEKTVLFAF	23.4	0.48	9.0	-0.1	-	[128]
**B8N5T3**	*Aspergillus flavus*	*rodB*	-	145	MKFFAVAALFATAAMAAPGSAPVPGAAAAAG NGNAPVINQTQQKAFTDACSAKGNHPVCC DQIDTSKTTTVNEGLLGGLLGEGLGGVLNNL VGGEPGACSGLVSALNKQCQTSIGCCQQNA KGDNYQSGLLNLNLQAPCLLSNGL	14.3	0.6	5.6	0.1	-	QRD89515.1 [128]
**P41746**	*Aspergillus fumigatus*	*rodA*	PF01185	159	MKFSLSAAVLAFAVSVAALPQHDVNAAGNG VGNKGNANVRFPVPDDITVKQATEKCGDQA QLSCCNKATYAGDVTDIDEGILAGTLKNLIGG GSGTEGLGLFNQCSKLDLQIPVIGIPIQALVN QKCKQNIACCQNSPSDASGSLIGLGLPCIAL GSIL	16.1	0.4	5.0	0.2	Secreted, spore wall	AAB60712.1 [106]
**E9QT94**	*Aspergillus fumigatus*	*rodB*	PF01185	140	MKFLAVVSLLAATALALPNAGVVHPTFASADK YTLQQAQNKCGEHTTLSCCNHVSKVGDTTA FNYGLLNGLLGNAISGPEGVGILSGCQKISVT ALIGVDDLLNKQCQQNVACCQDNKSVATG GLINIATPACVALDSII	14.3	0.3	6.7	0.4	Secreted, spore wall. Present in the conidium cell wall, but absent	EAL91055.1 [103]

										from hyphae of planktonic or biofilm cultures.	
**Q4WBR8**	*Aspergillus fumigatus*	*rodC*	PF01185	155	MLVTMRLSRSIAVFTLVTYATGLPSLQVIPRGEPQLPLLDPSMTIKEAARKCGDKAQLSCCNRVVKAGDYTSVDEGIGAGLLSNLAGGGSGISSILAFDQCSRLDAQVPVVLLPIQDLLNQHCKQNVACCQKNPGDASSSGVGVSLPCIALGSVA	16.0	0.2	7.6	0.3	Secreted, spore wall	EAL85466.1 [103]
**Q4WE22**	*Aspergillus fumigatus*	*rodD*	-	192	MPHFCLVASTSMLSASYDVSQDLSSALYTSSSTYKYESASARIDCLSTTCTQVLKTLEIEHKLSMKVFATLFLLAASATAALAAVPAPNAGCSQPGQYCNGGTFLCCNNRQCNNNVRVPYSVPGYARNPSYAFSRHHGRNGSSQLSMSIWDALEELLVSGGDWVSYNQWAKYEAGARVVGTIHERRGMNEID	20.9	0.4	7.4	-0.2	-	EAL86155.1 [103]
**Q4WC31**	*Aspergillus fumigatus*	*rodE*	PF01185	145	MHPRPEHLSPTLNHNHRLKLKQPATTSRMLARTILATLVSAACFATVNAQTCSATNAVCCQQLQDPDNLNADALNLLRLLNINPNTLTGAVGLTCKRLTEACFACLLLTRPGTSLVSGSCNANAACCTGNNYNGLIVLGCTQIQV	15.4	0.2	8.4	0.1	Extracellular region, cell wall	EAL85353.1 [103]
**Q4WEK0**	*Aspergillus fumigatus*	*rodF*	PF01185	190	MRPITILCTLATLSTTLAVPFSQASKSTSASRSTSSSTVPASLPSPTLSGPNACPPNKFKQCCTTLSQVGDDLLKPLGAVVPLVGAIQVNSLVGVSCRPMADAAPESTCGNAVMCCDSSTVGGDDLMQTSCQDFALAKKREREAIERQQRRFSEYQRMMLSQSATPAPTSTGVDVMGSSFSKAKATPTRV	19.8	0.1	8.5	-0.1	Secreted, cell wall	EAL85977.2 [103]
**Q4X055**	*Aspergillus fumigatus*	*rodG*	PF01185	125	MKPSIVTFLMLAAVTAAVSAEDPTMSALKSRVEEIAGQVHGVEDQETTPDLSHCVEPKLCCGSLTTPLDPILDPILLSLGINAAQIVGSVGLLCHPWTEECSSAPQCCTEANLLGGTLALGCSKL	12.9	0.3	4.2	0.4	Secreted, cell wall	EAL93760.2 [103]
**P52750**	*Aspergillus nidulans*	*DewA*	PF01185	135	MRFIVSLLAFTAAATATALPASAAKNAKLATSAAFAKQAEGTTCNVGSIACCNSPAETNNDSLLSGLLGAGLLNGLSGNTGSACAKASLIDQLGL	13.2	0.7	4.7	0.4	Secreted, spore wall	AAC13762.1 [65]

					LALVDHTEEGPVCKNIVACCPEGTTNCVAVDNAGAGTKAE						
**Q5BC93**	*Aspergillus nidulans*	*DewB*	PF01185	135	MKFIGLATLSLFALASADKFPVPDSITVAEGSSKCGDQAQLSCCNDVKYGGDTTVVQKGIAAGLLSDLLGAGSAAEGLGAFSGCSKLDISLLIGIEDILNQKCKQNIACCAKSGGSADGDLVGATLPCIALGSIL	13.4	0.3	4.3	0.5	Secreted, spore wall	CBF85659.1 [18]
**Q5AZ79**	*Aspergillus nidulans*	*DewC*	PF01185	162	MQFTIASLIATAVLGLQMASAAPHAPGSSNAAIISQAQEQNTCGNAHLSCCESTDNSVSLTQEEEEGLLHLLGGTSSVLSDGLLGKYSGCSSLASVEGILGAGGNQGLVSGQCNNHVACCDAGDNELVGFLEPGVAVLKPNSDGIQNGLANVAVPCVPVQVL	16.1	0.5	3.9	0.3	Secreted, spore wall	CBF69542.1 [18]
**Q5BEU0**	*Aspergillus nidulans*	*DewD*	-	101	MHLSTSAAAILALSLAGPTMAGRPYFCPLALDAKFLQVPYCCEGFVPARDSKVSFEGVNCIDVSSDEDFVKTCPKGGTPKCCYSIGPKVICTTEVGDGVDK	10.6	0.2	5.0	0.2	Secreted, spore wall	CBF88485.1 [18]
**Q5AVZ1**	*Aspergillus nidulans*	*DewE*	PF01185	109	MKVATALSVLAVAGSALASALPSAANSEKRQSDCIGPLLCCGSLTTPLDPLVDPILELVGIDAAAIVGSVGLLCHAYDDTCTSEPQCCTEANLLGGTLGLGCGPLEHGH	10.8	0.2	4.1	0.5	Secreted, spore wall	CBF79594.1 [18]
**P28346**	*Aspergillus nidulans*	*RodA*	PF01185	157	MKFSIAAAVVAFAASVAALPPAHDSQFAGNGVGNKGNSNVKFPVPENVTVKQASDKCGDQAQLSCCNKATYAGDTTTVDEGLLSGALSGLIGAGSGAEGLGLFDQCSKLDVAVLIGIQDLVNQKCKQNIACCQNSPSSADGNLIGVGLPCVALGSIL	15.6	0.2	4.7	0.3	Secreted, spore wall	AAA33321.1 [49]
**Q2U3W7**	*Aspergillus oryzae*	*RolA*	PF01185	151	MQFSVAAVLALATAVAALPPASGTGAGQQVGHSKNDFPLPKELTTKQAADKCGDQAQLTCCNKTVKTGDFTQVEEGLLAGLLSNLLGAGQGSQGLGLLDECTNIPVIPIISIASPQEKCKQPISCCQNTKSSADGDLVGIGLPCIALGSLL	15.2	0.4	4.7	0.2	Secreted, spore wall	BAE63748.1 [104]
**A0A7R7Y079**	*Aspergillus puulaauensis*	*HYD5*	PF06766	95	MVNLLQAIVSVSVGAIVASAVPLQPRANDVCPGTLYGNPQCCSIDVLGTADFDCSPPSSQPTSVDDFKTICGSEAKPAKCCTIPVAGLSSLCMNA	9.6	0.2	4.1	0.4	-	BCS30178.1 [17]
**J4KPV6**	*Beauveria bassiana*	*hyd1A*	PF01185	136	MRFALAITTLIAAVTAAPSGHHGGKDLHKMTLGQAGDRCGADQSIYCCNEKTTKIEHSSPETG	13.8	0.2	7.4	0.3	Secreted, cell wall,	EJP68119.1 [19]

					LGGLLGATVGADGLLSHLLGTCTKIPVNVLAV GNLLQSECTNRAACCHNTPSVAYGGLVNLA LPCVAIGSLIQ					spore coat, vacuole, cytoplasmic vesicle. Accumulates exclusively on the cell walls of aerial hyphae, conidia, vacuoles and vesicles of hyphae and blastospores.	
**J4W2A8**	*Beauveria bassiana*	*hyd1B*	PF01185	116	MLATTIFATLLALAAAAPHGPSHGPSVKTGDI CGNGNTMHCCNDESVTNKLTGPSVLSDLDL RKLLASECSPISVNVLLNQLVPIDNKCKQQSI CCGEQKQNGLVNLGCTPITVLG	12.0	0.3	6.7	0.2	Secreted, cell wall, spore coat, vacuole, cytoplasmic vesicle. Accumulates exclusively on the cell walls of aerial hyphae, conidia, vacuoles	EJP64605.1 [19]

										and vesicles of hyphae and blastospores.	
**J5JJC1**	*Beauveria bassiana*	*hyd1C*	-	89	MKFSLATIALAAAVAASPTNPPAGAGNCNVKGKTGNVTCCNSAIPILGQLACNVLASGTCNSGQTAYCCDTGGNTGLINVIALNCVKLL	8.7	0.1	8.2	0.6	Secreted, cell wall, spore coat, vacuole, cytoplasmic vesicle. Accumulates exclusively on the cell walls of aerial hyphae, conidia, vacuoles and vesicles of hyphae and blastospores.	EJP63421.1 [19]
**J4VS95**	*Beauveria bassiana*	*hyd1D*	-	89	MKFSLATIALAAAVVASPTNPPTEAGSCNVKGKTGNVTCCNSAIPILGQLLCNVLASGTCNSGQSAYCCDTGGNTGLIVVQAVNCVELL	8.8	0.2	6.1	0.6	Secreted, cell wall, spore coat, vacuole, cytoplasmic vesicle. Accumul	EJP61475.1 [19]

										ates exclusively on the cell walls of aerial hyphae, conidia, vacuoles and vesicles of hyphae and blastospores.	
**J4UGK4**	*Beauveria bassiana*	*hyd1E*	-	91	MKFSIAAIALAAVAVASPVEMEPRTNPSTCNINGNNNGKVVCCNSLIPILGQILCNIAVLGSTCNAGQTVRCCETDANGGLINISLLNCVG	9.3	0.1	6.1	0.6	Secreted, cell wall, spore coat, vacuole, cytoplasmic vesicle. Accumulates exclusively on the cell walls of aerial hyphae, conidia, vacuoles and vesicles of hyphae and blastospores.	EJP62022.1 [19]

**J4UJ50**	*Beauveria bassiana*	*hyd1F*	-	88	MLSRLFTVPAILLATLGSAATLAPREESACQRVCCDALVQSTSRGLVGINCNRGGIDCGFSGQIASCCAAIVPVGARQGTGVQCQRGN	8.9	0.1	8.2	0.4	Secreted, cell wall, spore coat, vacuole, cytoplasmic vesicle. Accumulates exclusively on the cell walls of aerial hyphae, conidia, vacuoles and vesicles of hyphae and blastospores.	EJP63742.1 [19]
**J4WDT5**	*Beauveria bassiana*	*hyd2A*	PF06766	97	MKSVVFASLIASALAVPTELAPRTDVSSLCPAGLYSVPQCCATDILGVARLDCKAPKQTPRNADEFKNICAKGGQQAACCVVPVAGQSLFCMTPVGI	10.0	0.2	8.0	0.5	Secreted, cell wall, spore coat, vacuole, cytoplasmic vesicle. Accumulates exclusively on the cell walls of aerial hyphae,	EJP68175.1 [19]

										conidia, vacuoles and vesicles of hyphae and blastospores.	
**J5JTB1**	*Beauveria bassiana*	*hyd2B*	PF06766	99	MKFFAIAALFAGALAMPTTEIEARTDKAVCPSGLYSNLVCADVDVLGILCLHATSPSETPRDARHFQEICAKVGKQARCAVLPVAEQAVLCQRPAGVSA	10.4	0.2	7.2	0.4	Secreted, cell wall, spore coat, vacuole, cytoplasmic vesicle. Accumulates exclusively on the cell walls of aerial hyphae, conidia, vacuoles and vesicles of hyphae and blastospores.	EJP68103.1 [19]
**J4WMI6**	*Beauveria bassiana*	*hyd2C*	PF06766	299	MKFLTVAAAIFASTSLAVPTNNHNGGGACVPRPNPGNSNTSSAPGGNPTGGHTSSGPGTKPTGSHTSSGPATKPTDGNTSSGPGTKPTDGNTSSGIDCEVPRHAPTSGDDFKGICAAVGKQPLCCVIPVAGQDLLCQDITGGNSPSGQPTSVPGGQSTSVPGGNPTSIPGGQPTSVPGGQPTSVPGGHPTSAPTGHPTSVPGGQPTSVPGGNP	29.0	0.3	6.7	-0.4	Secreted, cell wall, spore coat, vacuole, cytoplasmic	EJP70900.1 [19]

					TSVPGHHNPSPVGNDPSCCPSGLYSNPQC CAANVLGLADLDCSTPSQTFTTGDEFRNICA TIGRQPMCCVLPVAGQAVLCEKPVGV					vesicle. Accumulates exclusively on the cell walls of aerial hyphae, conidia, vacuoles and vesicles of hyphae and blastospores.	
**A0A384JA57**	*Botryotinia fuckeliana*	*Bhp1*	-	124	MRFSIATVVLSLAAMVVAIPTTESTLFARGGG QTCAQGQTLSCCQSVTSGGDGILGNLLGLN CAEIPIPIVGIVLGGKCNSAPVCCNVNGGSTS PDCDDNSNNGGTQGGINVLTNSCVAIPIVL	12.2	0.1	4.0	0.5	Secreted, cell wall	ATZ47525.1 [20–22]
**A0A384JW35**	*Botryotinia fuckeliana*	*Bhp2*	PF06766	98	MFFSRISTIVSMTALFASALAMPTTLTSRQDAI CSSGNPQCCDVDVLGVADLDCEAPPAAYTD IKSFSDVCADVGKINMCCDLPVLGQGLICSS PDNS	10.2	0.1	3.7	0.4	Secreted, cell wall	ATZ54816.1 [20–22]
**A0A384JJM8**	*Botryotinia fuckeliana*	*Bhp3*	PF06766	98	MQFTTTTLIAILSALAVASPIEPRQNATAQQER LCTSAIDTAMCCQTTLAGVINQTCTTPAITPIN KQAFRAYCAAQGQDSSCCKTPLVGDGVICTP P	10.2	0.2	6.1	0.2	Secreted, cell wall	ATZ50554.1 [20–22]
**Q00367**	*Cladosporium fulvum*	*hcf-1*	-	105	MQFTSFAILAISAVASARVTRRDDSSATGADK GGTCAVGSQISCCTTNSSGSDILGNVLGGSC LLDNVSLISSLNSNCPAGNTFCCPSNQDGTL NINVSCIPVSA	10.5	0.1	4.3	0.3	Secreted, cell wall	CAA67187.1 [25]
**O94217**	*Cladosporium fulvum*	*hcf-2*	-	121	MQFTTIVMTLAAAVAVTAYPGSSSAFGVGQD EHKHHSSDDHSATGASKGATCAVGSQVSCC TTDSSGSDVLGNVLGGSCLVDNLSLISILNSQ CPGANTFCCPSNQDGTLNIHAACIPVAL	12.0	0.1	4.8	0.3	Secreted, cell wall	CAB39309.1 [108]
**Q7Z9L5**	*Cladosporium fulvum*	*hcf-3*	-	102	MQFIASILAVPAVAYAVAIPDDNSATGASKGST CATGAQVACCTTNSSNSDLLGNVVGGSCLL	10.0	0.1	3.5	0.4	Secreted, cell wall	CAD92803.1 [108]

					DNLSLLSLNSNCPAGNTFCCPSNSDGTLNINAQCIPISA						
**Q7Z9L4**	*Cladosporium fulvum*	*hcf-4*	-	227	MQFTTFALLAVAAATASAQAPQAYYGQGAKSAQVHTFETRKAVPTRVAEVYGEHEQERVTKTKVYHALVTEEAQHHGEEHKAAPYKAYKVYSVASSYSAQPRATHAAEHYGEGKKADHYAEPAKAVHADPHHVDPVKARPTMAATEMKQPEKEAPSTVCAKGSEISCCTTDSSNSGALGNVLGGSCLLQNLSLLSLNSNCAAANTFCCPTTQEGTLNINLSCIPISL	24.1	0.7	7.1	-0.4	Secreted, cell wall	CAD92804.1 [108]
**O94203**	*Cladosporium fulvum*	*hcf-5*	PF06766	88	MQFLVLALASLAAAAPSIKLRAPSDVCPALDTPLCCQADVLGVLDLTCEAPSDDTSVSNFEAACATTGLTARCCTLPLLGEALLCTTP	8.9	0.1	3.8	0.7	Secreted, cell wall	CAB39312.1 [108]
**Q9C2X0**	*Cladosporium fulvum*	*hcf-6*	PF06766	184	MNFMLLSAALASMAVAGPIAGTYPITYPSSNTPATYPSGNAPIWSSPIHGGNNGGNGGNGGDNNGGNGGNGGSGGGNTGGNAGNGGGNNGGNNNGGNNGGNTGGEGGNGGNGGNGGAPVELCPANRVPQCCQLSVLGVADVTCASPSSGLTSVSAFEADCANDGTTAQCCLIPVLGLGLFCSNP	17.1	0.1	3.7	-0.2	Secreted	CAC27407.1 [111]
**Q8NIN9**	*Cladosporium herbarum*	*HCh-1*	-	105	MAFIKSLLIASVAAVAFAAPQGGASDNNKKVEIDGQDSAPVCGNGQKVACCNSGEDLIGLNCLSIPILAIPIQKACGSNIAACCQTGDSEGNLLNLEANCLAIPL	10.5	0.2	4.1	0.4	Secreted, cell wall	CAD42710.1 [129]
**Q9UVI4**	*Claviceps fusiformis*	*TH1*	PF06766	394	MKFLAAASLLVASTLAVPTSSGGSCRPRPPPGGGNGGNGGNGGNGGNGYQPCPAGLYSNPQCCATDVLGVADLDCKNPSSAPMSGDNFKSICNAVGQQAKCCVLPVAGQAVLCQDSINGGGNGGNNGGNGGNNGGNGGNNGGNTDYPGGNGGNNGGNNGGNNGGNNGGNNGGNNGGNNGGNNGGNNGGNNGGNGGNGGNGYQACPAGLLYSNPQCCSTGVLGVADLDCKNPSSAPTSGDDFQKICANGGQQAQCCSIPVAGQAVLCQPAIGGGNPGGNGGNNGGNGGNGGNNGGNNGGNGDYPGGNGGNNGGSNGGGNGGNGGNGGSFKCPSGLYSVPQCCATDVLGVADLDCGNPSRQPTDSSDFASVCAAKGQRARCCVLPLLGQAVLCTGA	36.8	0.3	4.6	-0.5	Secreted, cell wall	CAB61236.1 [74]

**Q8J1W4**	*Claviceps purpurea*	*cpph1*	PF06766	756	MKFLAVSSILFASSLAVPTSHGGGSCRPRRPP PVNYPGGDYPGGQGGDKPPGGGYPGTTYP GGDYPGGDHPGGYGGGGYGGGDYPGGDY PGGDYPGGDYPGGDYPGGDYPGGNHGGG NHGGGNGGNNGGGNGGGNGGGGNNGN GTDPGGNNGGGNGGGGNVPYKPCPSGLYS NPQCCSTNVLGVAALNCKTPSFAPTSFQSFK SACSGGQPQCCVVPAAGQGVLCQAPIGGG NGGNNGGNNGGNNGGNNGGNNGGNNG GNNGGNNGGNGGNGGNNGGNNGGNGG NNGGNGGNGGDGGDYSACPSGLYSNPQC CATDVLGVASLDCVNPSSKPRSGDDFKSTC GAEGKHASCCAIPVAGQGVLCQPAMGGGG NGGNNGGGNGGNGGNGGNGSNDGGNG GNGGNNGGNNGGNGGNGGDGGDYSACP SGLYSNPQCCSTDVLGVASLDCVNPSSKPRS GDNFKSTCAAAEKHASCCVIPVAGQAVLCQ PAMGGGGNGGNNGGNGGNNGGNNGGN NGGNNGGNNGGNNGGNNGGNGGNGGN NGGNNGGNGGNGGNNGGNNGGNGGNG GGNGGDYSACPSGLYSNPQCCSTDVLGVAS LDCVNPSSKPRSGDNFKSTCAAAGKHASCC VIPVAGQGVLCQPAMGGGGNGGNNGGGN GGNGGNNGGNNGGNGGNNGGNNGGNG GNNGGNGGGSHEVCPSGLYSVPQCCATGL LGVLDLDCQSPSRTSFSSSDFKSTCRSEGRK ARCCVLPVAGQDVLCTNPL	69.9	0.2	5.3	-0.7	Cell envelope	CAD10781.1 [26]
**W8P1J0**	*Clonostachys rosea*	*Hyd1*	PF06766	98	MQFFTTVVLFAAAAMALPGGQQSRPYDPCP NNLLSVPQCCDTDALGLVGLGCVAPPNNRP ASPRAFADCCHASSRRPKCCAVTTGLEASVL CDDPAGF	10.1	0.2	5.7	0.2	Secreted, cell wall	AHL20218.1 [158]
**W8NXX4**	*Clonostachys rosea*	*Hyd2*	PF06766	123	MRSFLVIATLAVGAFGQSYDPCPNDLKNHAL CCNAGLENLINFDCHTRKFDSLLPPFQATCA RTGSYPKCCTIKIISQGLLCEDPAGVENPDTD TFNPEGDNELENLLDDVGNILNEVLNLLN	13.4	0.4	4.1	-0.1	Secreted, cell wall	AHL20219.1 [158]
**W8NQ71**	*Clonostachys rosea*	*Hyd3*	PF06766	99	MRIDILATAALLAQLASASPAPAKRQTYIACSG LYGTAQCCATDVLGLVNLDCGNPPSLPTSPD	10.4	0.3	6.2	0.5	Secreted, cell wall	AHL20220.1 [158]

					EFSSVCSAIGQRARCCVLPIVSFMRLIIQDYPISY						
**A0A8H3W1T0**	*Colletotrichum asianum*	*gene14730*	PF06766	103	MQFTTALIAIFASVAIAAPTGDGHNGGGSTPAPYDPCSGLYDSAQCCATDVLGVANLDCASPTSVPSSANDFRKICAVGGQRARCCVLPVLGQAVLCQTPVGV	10.3	0.2	4.6	0.4	Cell envelope	KAF0320716.1 [23]
**L2G0I0**	*Colletotrichum fructicola*	*rodA*	PF01185	138	MRFSAVTVSALAMALTVAAAPGNTAKEALAKRTDDLTVADAQNICGKDLSVNCCNQVDASTNNNDNSGSGILSGILGGVLGNGGLKLTDGCSSIGVGIANDLLNSQCKQSVACCKTDGNTASGLVAVQLPCIPISGLL	13.7	0.1	4.6	0.3	Secreted, cell wall	ELA31811.1
**L2G430**	*Colletotrichum fructicola*	*CRP*	PF06766	97	MQFLVAVAALFASVAVSAPLDERQAYVACSGLYGTAQCCATDVLGVADLDCGNPPETVTSADNFSEVCSAIGQRARCCVLPILDQGILCNTPTGVSD	9.9	0.2	3.7	0.5	Cell envelope	ELA33409.1
**L2G1J6**	*Colletotrichum fructicola*	*CU*	PF06766	103	MQFTTALIAIFASVAVAAPTGDGHNGGGSTPAPYDPCSGLYDSAQCCATDVLGVADLDCASPTSVPSSANDFRKICAVGGQRARCCVLPVLGQAVLCQTPVGV	10.2	0.2	4.3	0.4	Cell envelope	ELA32569.1
**A0A6G6VYL3**	*Colletotrichum siamense*	*CsHydr1*	PF01185	138	MRFSAATVSALAMALTVAAAPGNTAKEALAKRTDDLTVADAQNICGKDLSVNCCNQVDASTNNNDNSGSGILSGILGGVLGNGGLKLTDGCSSIGVGIANDLLNSQCKQSVACCKTDGNTASGLVAVQLPCIPISGLL	13.6	0.1	4.6	0.3	Secreted, Lipid droplet. Mainly localizes to intracellular lipid droplets in a Cap20-dependent manner.	QIG38049.1
**E0WHQ8**	*Cordyceps farinosa*	*ah2*	PF01185	134	MRFAFAITALIAAVTAAPSKDLAIVNKRHGMTVHDAAKKCGTGQQLNCCNEQHSNEGDKGQVSKPTGALVTADGLLSHLIGTCGKLDVAALIGIDDLLNTNCKGQAACCQDTASVAQGGLLNLALPCIALDSLL	13.7	0.1	6.9	0.3	cell wall	CBV17211.1 [163]

**A0A2H4SBS0**	*Cordyceps militaris*	*CmHYD1*	PF06766	105	MQVLLIATLVASVLAAPTEVSPQTSGGGGGGGGGGGVCRGFLYSNPICCATDILGLACLDGDTPPETPRDAKNFKEICAKNGQQARCCVLPIIDQAVLCIRPLGV	10.6	0.4	4.9	0.4	Secreted, cell wall	ATY60554.1 [24]
**A0A2H4SHM4**	*Cordyceps militaris*	*CmHYD2*	PF06766	99	MKFFVVAALFAGALAAPTELEARTYPVVLCPEGLYANPVCADVDVLGILCLNAVSPSEAPRDANHFREICAKIGKQARCAVLPVLNQAVLCNKPVGITN	10.5	0.1	7.0	0.5	Secreted, cell wall	ATY62608.1 [24]
**A0A2H4SKW2**	*Cordyceps militaris*	*CmHYD3*	PF01185	138	MRFFLAITALVAAVTAAPSADVAAVNQMAAMTLAEADASCGNGASLYCCDKSKQGGDTGSPGQAGGVGLLGAIVGVNGLLQGLLGQCSKININAIGAAGVLNQECTARAACCQNTPSVAHHGLVNIALPCIPINSLVG	13.5	0.1	6.5	0.6	Secreted, cell wall	ATY63719.1 [24]
**A0A2H4SI69**	*Cordyceps militaris*	*CmHYD4*	PF01185	104	MFASTVFVSLLAVAAAVPTNPQGQSCGNGNTVHCCNAETANKLTKGGILGALDLSNLLGQCNDVTAAVAGGAVPIKNTCSSQAVCCGEIHQNGLVNLGCTPIFV	10.3	0.2	6.1	0.5	Secreted, cell wall	ATY62804.1 [24]
**P52753**	*Cryphonectria parasitica*	*Crp*	PF06766	118	MQFSIIAISFLASLAMASPAKRGGGGGGSGSGSGSGSGSGSGGGSTTYTACSSTLYSEAQCCATDVLGVADLDCETVPETPTSASSFESICATSGRDAKCCTIPLLGQALLCQDPVGL	11.4	0.3	4.0	0.3	Secreted, cell wall	AAA19638.1 [27]
**Q6YF32**	*Fusarium verticillioides*	*HYD1*	PF01185	126	MQYMTIVAFLAATVAAGPQIRAYPSIDQITVAQANNACGNNMQVTCCNKVTNTPAGNAVGNGAGILNNLSLFDQCSKLDVNVLAIANGLLNKECQANAACCQNSGGSATGGLVNVALPCIALSSLI	12.7	0.1	6.1	0.4	Secreted, cell wall. Localizes to the outside of conidia, and more particularly to the junctions of individual microconidia.	AAO16867.1 [133]
**Q6YF31**	*Fusarium verticillioides*	*HYD2*	PF01185	123	MYAYTVIAFLAASVAAAGNGPSISSLTVQQAANSCANGQSVYCCNKTSNKPAGNSVGDGAG	12.1	0.1	6.1	0.4	Secreted, cell wall. Localizes	AAO16868.1 [133]

					IANGLSLFSQCSKVDVNVIAIANNLLNKECQANAACCQDSPGTAAAGLVNAALPCVAISNLV					to the outside of conidia, to the junctions of individual microconidia	
**Q6YF30**	*Fusarium verticillioides*	*HYD3*	-	96	MQFAKIASVLAMAAAAVAAPAPGNYEVEPRTGGSNNGNNQPACSAQSSNVCCNGLGCLVQILGAGCSTKSYCCQSDAPLAVGALVNVNALNCVQVL	9.5	0.1	6.1	0.4	Secreted, cell wall	AAO16869.1 [133]
**Q6YF29**	*Fusarium verticillioides*	*HYD4*	PF06766	100	MQFYAIASLFLAGTAFAAPATSPNGYDACPDGGLIGTPQCCSLDLVGVLSGECSSPSKTPNSAKEFQEICAASGQKARCCFLSEVFTLGAFCQKPVGVTA	10.2	0.2	4.5	0.3	Secreted, cell wall	AAO16870.1 [133]
**Q6YD93**	*Fusarium verticillioides*	*HYD5*	PF06766	98	MQFSLALVTLLATAVSALPTEEKRQAYIPCSGLYGTSQCCATDVLGVADLDCGNPPSTPANATDFSAVCSAIGQRARCCVLPILDQGILCNTPTGVQD	10.1	0.5	4.0	0.3	Secreted, cell wall	AAN76355.1 [133]
**I7B278**	*Geosmithia pallida*	Geo1	PF06766	109	MKSFAITAVLFAAAAMAGPLEVRTGGGGGGGGGGGSAYDACPSELLSNPECCDVDVLGLLSINCAPPDNTPTSAADFKSQCAKRGKSATCCVLPVLGQAVGCQAPAGSG	10.5	0.1	4.5	0.3	cell wall	AFO38193.1 [28,29]
**A0A7D5LZ09**	*Hypocrea atroviridis*	*hfb4*	PF06766	65	GPCTGGLTNNIPQCCGAGVLDLLYLDCESPRVVSSALNPLQSICAARGLEAKCCTVGIAGLGVLC	6.5	0.6	4.5	0.7	Cell envelope	QLH01849.1 [85]
**P52751**	*Magnaporthe grisea*	*MPG1*	PF01185	112	MFSLKTVVLALAAAAFVQAIPAPGEGPSVSMAQQKCGAEKVVSCCNSKELKNSKSGAEIPIDVLSGECKNIPINILTINQLIPINNFCSDTVSCCSGEQIGLVNIQCTPILS	11.6	0.7	4.9	0.4	Secreted, cell wall	AAA20128.1 [119]
**E9DZB1**	*Metarhizium acridum*	*HYD3*	-	91	MLFRLFTIPSIALGVLGSAATLPPREESANCRSVCCDAIVPSIFPSGRVGINCHWDELLDCGFSGQVDSCCEAIVPFGVKDGTGIRCSRRQ	9.7	0.1	5.8	0.3	Secreted, cell wall. Localizes on the surface of conidia.	EFY91073.1 [30]

**P52752**	*Metarhizium anisopliae*	*ssgA*	PF01185	96	MFKALIVALAAVAAAIPTQQPSSNEMNCDSGVYCCNKVAQNTGIVVPIDALSSTCGDTLKLVTVDALNDKCTSQTVCCNNVQQNGLVNVACTPIDV	9.9	0.3	4.1	0.4	Secreted, cell wall	AAA33418.1 [120]
**A0A7D5Z1U5**	*Metarhizium brunneum*	*hyd1*	PF01185	94	MFKALIVALAAVAAAIPTQQPSSNKIECNNTVFCCDKQTVGQAGIPIDALNPTCTNLNVLAVDALNPPCPGQTVCCNNVQQNGVVNVACTPISA	9.6	0.1	4.5	0.4	Secreted, cell wall	QLI69905.1 [31]
**A0A7D5YYR3**	*Metarhizium brunneum*	*MPG1*	-	430	MPTHRGFLTASLAKLALIAPFLAQQAIAGDIEARTVEVFATTYLDLGHCHNACTTKKWHEWETPGHESPPYATHTMPAEEGCVTTSISTKHPECQKCKGTVYVYVPKTEHHAKTTTVTKSVHTSTQHTTKPTTVKTWTRPSSSSKPVTESKSSTTKREPTTTKKETTSTKKEPTTTKREPTTTTKREPTTKRTNDYQDMDMGYKQTTIFDQDVHYDERANFIFNEDMDMDMDFNQATLFYQDVNCDERANDYKERANDYQAPLSTKTSTATKEPTSTKREPTTTKREPTSTKHETTTTRTWTWTSKPLSTKTSTATKEPTSTKREWTTTKRESTSTKRETTSTKRYPRPTPHSGPTVGDANAQCGNHQVLSCCRSGNSAGLLNNVLGGQCSPINVLIALVPLQNACSNQVACCTGSANGLLNVECTNLNV	47.7	0.5	8.6	-0.9	Cell wall	QLI69384.1 [31]
**K7NX97**	*Metarhizium rileyi*	*hyd*	PF01185	112	MAFFKVLVAAATLATALALPSGGSGNGNGNGHGHSVGDAAAQCGNHQQLSCCNKGNSAGTLLDGLLGGNCSPLDLSIIGVGVPLSTACSNQVACCTGDQNGLLNLACTNLNL	10.9	0.1	6.2	0.3	Secreted, cell wall	AFA43698.1
**A9Q070**	*Metarhizium rileyi*	*hpb*	PF01185	92	SGGSGNGNGNGHGHSVGDAAAQCGNHQQLSCCNKGNSAGTLLDGLLGGNCSPLDLSIIGVGVPLSTACSNQVACCTGDQNGLLNLACTNLNL	8.9	0.5	5.3	0	Secreted, cell wall	ABO72628.1
**E9FDE9**	*Metarhizium robertsii*	*hyd1*	PF01185	102	MSLFKILVAAATVATALAAPHEHKKPHPSYGDANAQCGNHQKLSCCNRGDSGGVLDGLLGGNCQPINILALLPIQNQCTNQVACCTGNSNGLINIPCTNVNL	10.5	0.4	7.7	0.1	Secreted, cell wall	EFY94230.1 [30,32]
**E9FBT2**	*Metarhizium robertsii*	*hyd2*	PF01185	96	MFKALIVALAAVAAAIPTQQPSSNEMNCDSGVYCCNKVAQNTGIVVPIDALSSTCGDTLKLVTVDALNDKCTSQTVCCNNVQQNGLVNVACTPIDV	9.9	0.3	4.1	0.4	Secreted, cell wall	EFY94798.1 [30,32]

**E9EN44**	*Metarhizium robertsii*	*hyd3*	PF06766	95	MKLLAVAALLAGAAIAAPTSNTGGGSVCPSGLYSNPQCCSTVIIGVIALDCKSPSEVPRDGTDLRNICAKTGQKAACCVIPVAGQALLCETAPGA	9.3	0.1	7.6	0.6	Secreted, cell wall	EFZ04108.1 [30,32]
**A1DEM6**	*Neosartorya fischeri*	-	PF01185	159	MKFSLSAAVLAFAVSVAALPQHDVNAAGNGVGNKGNANVRFPVPDDITVKQATEKCGDQAQLSCCNKATYAGDVTDIDEGILAGTLKNLIGGGSGSEGLGLFNQCSKLDLQIPIIAIAVQDLVNQKCKQNIACCQNSPSDASGSLIGLGLPCIALGSIL	16.1	0.4	4.7	0.3	cell wall	EAW17833.1 [102]
**Q7S3P5**	*Neurospora crassa*	*NC2*	PF06766	97	MQFTIATVLSLLTITLAAPAAMERQVPYTPCSGLYGTAQCCATDVLGVADLDCANPPATLANATHFESTCAAIGQRARCCVLPILGQDILCQTPAGL	10.0	0.7	4.3	0.6	Secreted, cell wall	EAA30046.2 [33]
**Q04571**	*Neurospora crassa*	*EAS*	PF22354	108	MQFTSVFTILAIAMTAAAAPAEVVPRATTIGPNTCSIDDYKPYCCQSMSGPAGSPGLLNLIPVDLSASLGCVVGVIGSQCGASVKCCKDDVTNTGNSFLIINAANCVA	10.9	0.3	4.3	0.6	Secreted, spore wall	CAA47754.1 [34]
**A0A0C3D4E3**	*Oidiodendron maius*	*OmSSP1*	-	93	MKYITAISMLATAALTAATPLNGVEARWGSGGGGGGSTCDNNQQQVCCASVLSLICLVDVLGGNCNGGSYCCDNGASVGGLINLNLLNCVQIL	9.2	0.1	4.1	0.5	Secreted, cell wall	KIM96792.1
**Q06153**	*Ophiostoma novo-ulmi*	*CU*	PF06766	100	MQFSIATIALFLSSAMAAPYSGNSNSDSYDPCTGLLQKSPQCCNTDILGVANLDCHGPPSVPTSPSQFQASCVADGGRSARCCTLSLLGLALVCTDPVGI	10.2	0.2	4.3	0.3	Secreted, cell wall	AAA18912.1 [124]
**Q8NJQ1**	*Paracoccidioides brasiliensis*	*Pbhyd1*	-	100	MQLSNIVAVVALLATTAVASRSPPRPQRPSRPVAPVFQSIQCSSGSPFCCAPESTLGTTCTAMSGSSVQCNSLIVCCNNNGSGTQVCSATLQQPVNFVDL	10.3	0.1	8.0	0.3	-	-
**Q6T935**	*Paracoccidioides brasiliensis*	*Pbhyd2*	PF01185	133	MKFSLTAVVLALATAAVAAPGGNYKGAGPREEGPSISRQCGQAQISCCNKQISTVNGGDKNSGLLNGVLGTVIGKGGSVGIFDQCSKLSLSALIGVTDNLNSQCKQTVACCQGDSKAEGLVALNLPCIPIAAL	13.3	0.1	8.2	0.3	Secreted, cell wall	AAR11449.1 [2,126]
**A0A0F7TCZ5**	*Penicillium brasilianum*	*rodA*	PF01185	164	MQFTISALISLAALATALPPVGPSAGGFGNANGVDNKGNTQIRFPVPDDLTIKQAQAKCGDQAQLSCCNHATYAGDTTDVSKGLLAGTLGHLIG	16.4	0.4	4.4	0.2	Secreted, cell wall	CEJ54629.1

					SGSGSEGLGLFDQCSQLDVQVPILLGLIGVQDLVKKNCQQNIACCQNSPSGAGDDLVGAGLPCIALGALL						
**Q1I187**	*Penicillium camembertii*	*rodA*	PF01185	164	MRFSISALVLGLAATVYALPPGAPSAGGAGSGNGVGNKGNTDVRFSVPDNMTVKQAQAECGDQAQLSCCNKAVYAGDTTDINSGILGGTLSNLIGSGSGASGLGLFDQCSKLDLQIPVLIGIPIQDLINQKCKQNIACCQNSPSSANSDLIGLGLPCVALGSIL	16.4	0.4	4.6	0.3	secreted, cell wall	AAY88881.1 [145]
**A0A0A2K7M3**	*Penicillium expansum*	*HfbA*	PF01185	227	MQFSLSAIVLGLAATVYALPPSAPVAGGAGAGNGVGNKGNTDVRFSVPDNLTVKQAQAKCGDQAQLSCCNKAVYAGDTTDINSGILGGTLSNLIGSGSGASGLGLFDQCSKLDLQVPILALLPIQDLVNQKCKQNIACCQNSPSSAVSTPPIIDQKAQLTHHRTPTSLVLASPVLPLAPSSKRLAMFSRIMQAALVITPALWTLVQSSTYAEMWIVIDSEDGKQAFD	23.4	0.2	7.2	0.2	Secreted, cell wall	KGO62871.1 [35]
**A0A0A2JSZ6**	*Penicillium expansum*	*HfbB*	PF01185	156	MQFTLSAVVLALAGFSAALPAESMNNGLQHQNGTPRFPVPDSLTIEQAQAKCGDQAQLSCCNKATYAGDTTDINSGILGGTLSNLIGAGSGASGLGLFDQCSKLGAQIPIVIGIPIQDILNQQCKQNIACCANSPSTASGNLVGAALPCVALGSIL	15.7	0.3	4.5	0.3	Secreted, cell wall	KGO56793.1 [35]
**A0A0A2IC94**	*Penicillium expansum*	*HfbC*	PF01185	140	MKFFVPAFLFAATAMALPGSGSAAGVAPHSNNEVEDAVNKCGDGAHMSCCNKINKNEGISQNNIGVLSNVLGAIGSEGLGLGQGCTQLDIPISVLGAAGLTDFLKKNCQQNIACCQNSNSQANGNLVGVAAPCVSFGGLL	14.0	0.2	5.6	0.3	Secreted, cell wall	KGO63375.1 [35]
**A0A0A2K253**	*Penicillium expansum*	*HfbD*	-	105	MKFYIVLLALAAFAMAEADPNLEARAGCSQKGKYCNGGTFLCCPGQGSCKGNQIAKKKYSASEAPSTEGVQRLLLVTGYALECLGCLFSQTPGYSGSVLPLLNSH	11.0	0.4	8.3	0.1	Secreted, cell wall	KGO61797.1 [35]
**A0A0A2KI06**	*Penicillium expansum*	*HfbE*	-	170	MQLTTLLTGLISVLSVTTAIPMGSESPTPTSSVSKSLIAHSSSASVSASPTPSPNPYEAYTCPKDKFKACCMSVQQTGKDIVKQLGDLVPVLSGLQVSSAISFQCKNMTEREAPDSCNGQGYTPMCCNTKVEDTGFNTCKPFEDVKKAYYMNNMKDIPESQADMIMDILT	18.2	0.5	4.9	-0.2	Secreted, cell wall	KGO57376.1 [35]

**A0A0A2JK41**	*Penicillium expansum*	*HfbF*	-	97	MRVSALAFAAVLSLVSAKKINMHCNFAEDHTGMVQQPYCCRDLVPARGNSKANEALDCDQLDQPQLCDDQSRPACCYTIGAKKICTSHVIFQDAEDV	10.6	0.6	5.6	-0.2	Secreted, cell wall	KGO55739.1 [35]
**A0A0A2J434**	*Penicillium expansum*	*HfbG*	PF01185	109	MRLSILSVFSLVGAGMVSALPQESNLSMKRSDTSECVGPLLCCGTLTTPLDPVVDPLLLALGIDAANIVGSIGLLCHGYEESSCPTKPQCCTEANLLGGTLALGCADLK	11.2	0.2	4.2	0.5	Secreted, cell wall	KGO58235.1 [35]
**W6R223**	*Penicillium roqueforti*	*rodA*	PF01185	164	MQFSLSAIVLGLAATVYALPPGGASAGGAGSGNGVGNKGNTDVRFAVPDNMTVKQAQAKCGDQAQLSCCNKAVYAGDTTDINSGILGGTLSNLIGSGSGASGLGLFDQCSKLDLQIPVLIGVPIQDLINQKCKQNIACCQNSPSSANSDLIGLGLPCVALGSIL	16.3	0.3	4.8	0.3	Secreted, cell wall	CDM35882.1 [36]
**A0A3N2PQC3**	*Sodiomyces alkalinus*	*Sa-HFB1*	PF06766	99	MKFIAVVAALTASLAMAAPTESSTDTTYIACPISLYGNAQCCATDILGLANLDCESPTDVPRDAGHFQRTCADVGKRARCCAIPVLGQALLCIQPAGAN	10.2	0.2	4.8	0.4	Secreted, cell wall	ROT36721.1 [37]
**A0A7D5LY13**	*T. longibrachiatum*	*Hfb4*	PF06766	65	GPCSDGVTNNIPKCCGAGILDLLYLDCKTPTQVTSILNPLSAVCGRVGLQAKCCTLGIAGLGVLC	6.5	0.6	7.6	0.7	Cell envelope	QLH01968.1 [85]
**A0A7D5R129**	*Trichoderma aethiopicum*	*hfb4*	PF06766	65	GPCSGGVTNNIPKCCGAGILDLLYLDCKTPTQVTSVLNPLSAVCGRVGLQAKCCTAGIAGLGVLC	6.4	0.6	8.0	0.7	Cell envelope	QLH01973.1 [85]
**A0A6C0VP10**	*Trichoderma afroharzianum*	*hfb4*	PF06766	102	MKFSAIALFASLAIAAPATDAADATQFDIYDGPCSAGVTNNIPKCCGAGVLDLLYLDCETPQEVTSPLNPLHAVCARVGLSAKCCTLGIADLGVLCQDALPQ	10.4	0.2	4.0	0.5	Cell envelope	QIB98243.1
**A0A7D5AJD4**	*Trichoderma afroharzianum*	*hfb10*	PF06766	131	MKFLTVAAVFFTAVLAAPGNYPPPPPPTYAPPPPTYTLPPNGNGNGNGNGNGNGNNSHAPLCPSGLLYNTPLCCATDVLGVADLDCATPSTLPINPADFQKVCAAKGQRPRCCVLPLVGQGLLCTTPIGTQ	13.3	0.3	7.6	0	Cell envelope	QKV26102.1 [85]
**A0A7D5QXT8**	*Trichoderma aggressivum*	*hfb4*	PF06766	65	GPCAGGVTNNIPKCCGSGILDLLYLDCETPTQVTSVLNPLSAVCGRVGLQAKCCTLGIADLGVLC	6.5	0.7	4.5	0.7	Cell envelope	QLH01913.1 [85]
**-**	*Trichoderma aggressivum*	*TAE_1*	-	-	Automatic annotation (Putative)	-	-	-	-	-	ES768856 [168]

**-**	*Trichoderma aggressivum*	*TAE_2*	-	-	Automatic annotation (Putative)	-	-	-	-	-	ES768855 [168]
**-**	*Trichoderma aggressivum*	*TAE_3*	-	-	Automatic annotation (Putative)	-	-	-	-	-	AJ904501 [168]
**A0A7D5R606**	*Trichoderma alni*	*hfb4*	PF06766	65	GPCPSGVTNNIPKCCGSGILDLLYLDCKTPTQVTSALNPLSAVCGRVGLQAKCCTVGIADLGVLC	6.5	0.6	7.6	0.6	Cell envelope	QLH01917.1 [85]
**A0A7D5R667**	*Trichoderma andinense*	*hfb4*	PF06766	65	GPCSSGVTNNIPKCCGAGILDLLYLDCKTPTQTTSVLNPLSAVCGRVGLQAKCCTAGIAGLGVLC	6.4	0.5	8.0	0.6	Cell envelope	QLH01977.1 [85]
**A0A6V8R0V1**	*Trichoderma asperellum*	*TasHyd 1*	-	145	MYASVIIYTLVALCGVMSSPVQEAEVAPRAIEPRMPTFADIPFPRFPSHNNNHHHNEKDKGKDKGNGKGVDKNNGGGCDTGTNTQLNACSAGSPYCCSSDGNGGHICSNTTACDQKVICCNNNNGFQICIGEIDFNVPVTINIIYD	15.5	0.4	6.0	-0.4	Secreted, present in the trifluoroacetic-acid-soluble fraction of mycelial cell walls	GFP57915.1
**A0A2T3ZKC8**	*Trichoderma asperellum*	*HFB2-1*	PF06766	88	MKFFIATIFATGALAISVCPTGLYSNPQCCGANILGVAALDCHTPRAPILPGALFEAVCSDEGGKEPLCCTIPVAGQDLLCVAPVGTA	8.9	0.1	4.4	0.8	Secreted, cell wall	PTB45256.1
**A0A2T3ZC06**	*Trichoderma asperellum*	*HFB2-2*	PF06766	89	MKLYIAAALLTLGLAAPITETESHSIASRGSFSCPGGLINSSPMCCSVNVLGLLALDCRNVGPDGCVGSSKPNCCTLGTAGQGLICNEM	8.9	0.2	5.6	0.5	Secreted, cell wall	PTB42336.1
**A0A2T3Z3L3**	*Trichoderma asperellum*	*HFB2-3*	PF06766	97	MKFFAAAALFIAGVLAAPSPNAANSVTPLCNPGLYSNAQCCAVDVLGVADLNCATPPGVVNNAAEFQAACAKIGQEARCCVLPVLGQDVLCETPLGL	9.7	0.2	4.1	0.7	Secreted, cell wall	PTB39397.1
**A0A2T3ZJV9**	*Trichoderma asperellum*	*HFB2-4*	PF06766	97	MKFFTAAALFAAVAIAGPVEVRTGGGSICPDGLFSNPQCCDTQLLGIIGLGCEVPSQTPRDGADFKNICAKTGDQALCCVLPIAGQDLLCQAAVGAA	9.7	0.2	4.2	0.5	Secreted, cell wall	AUB45113.1
**A0A2T3ZCF4**	*Trichoderma asperellum*	*HFB2-5*	PF06766	101	MQLTALLALATLAIAAPAEPSEVAPRNVLTGPCSSGVTNSNPQCCGAGILGILYFDCETPDEVS	10.1	0.4	3.8	0.5	Secreted, cell wall	PTB42484.1

Journal of *Fungi*

MDPI

					SPINPLKTICAAEGLQAKCCTLGIAGLGVLCTDALPE					
**I7AQG9**	*Trichoderma asperellum*	*HFB2-6*	PF06766	106	MQFFTVATLFLATAFAAPSVDTNGNGIAPRLAANFCPPGLLYSNPQCCQIDVLGVADLDCVSPPSGPSKCKTFAGICDKIGRQPKCCVVPVAGQALLCTDPVGANN	10.9	0.2	6.1	0.4	Secreted, cell wall	AFN88524.1 [113]
**A0A2T3YYV2**	*Trichoderma asperellum*	*HFB2-7*	PF06766	142	MKFLTVAIVLAAAASAAPTKEVLPYPPVVAPPPYGGIAPDHNPPFGGGIPPINSGGLPPTNGAYPGGNNRHLCPSGLLYTNPQCCSAGVLGVADLDCKTPSSVPMNGADFERICAADGSKAQCCSVPVAGLALLCQDAIGTN	14.1	0.2	5.6	0.2	Secreted, cell wall	PTB37727.1
**A0A2T3ZJ73**	*Trichoderma asperellum*	*HFB2-8*	PF06766	160	MPASNSCSPPGGDHKPPPPPPSHPSPSPTPSYPPPPNNGGGYYPPPPDNTSHTPPPPSSTPPNNGGGGNDGGSGNTGGGNSDDGSDGGHSICPSGLYSSAQCCSTDVLGIADLNCKPPSRTPKDGSDFAKICSNTGSEARCCVIPVAGQALLCEDVVGAN	15.7	0.4	4.5	-0.8	Secreted, cell wall	PTB44813.1
**A0A7D5LXR1**	*Trichoderma atrogelatinosum*	*hfb4*	PF06766	65	GPCTSGVTNNIPKCCGAGLLNLLYLDCKTPTQSTSVLNPLSAVCGRVGLQAKCCTLGISDLGVLC	6.6	0.6	8.0	0.5	Cell envelope	QLH01908.1 [85]
**A0A7D5R646**	*Trichoderma capillare*	*hfb4*	PF06766	65	GPCPAGVTNNIPKCCGAGILDLLYLDCKTPTEVTSPLNPLSAVCARVGLQAKCCTIGIDGLGVLC	6.5	0.6	6.1	0.6	Cell envelope	QLH01955.1 [85]
**A0A7D5RAH7**	*Trichoderma catoptron*	*hfb4*	PF06766	65	GPCPSGVTNNIPKCCGSGVLDLLYLDCKTPTQATSVLNPLSAVCGRVGLQAKCCTVGIADLGVLC	6.5	0.5	7.6	0.6	Cell envelope	QLH01939.1 [85]
**A0A7D5R5F0**	*Trichoderma ceramicum*	*hfb4*	PF06766	65	GPCPSGVTNNIPKCCGSGILDLLYLDCKTPTQATSVLNPLSAVCGRVGLQAKCCTVGIADLGVLC	6.5	0.5	7.6	0.6	Cell envelope	QLH01936.1
**A0A7D5QYV0**	*Trichoderma cerebriforme*	*hfb4*	PF06766	65	GPCTGGVTNNIPQCCGAGILDLLYLDCESPREVSSVLNPLKAICARHGLEAKCCTLGIAGLGVLC	6.6	0.5	5.6	0.5	Cell envelope	QLH01831.1
**A0A7D5LXX3**	*Trichoderma chromospermum*	*hfb4*	PF06766	65	GPCSSGVTNNIPKCCGSGLLDLLYLDCKTPTQATSALNPLSAVCSRVGLQAKCCTLGISDLGVLC	6.5	0.7	7.6	0.5	Cell envelope	QLH01860.1
**A0A7D5M100**	*Trichoderma citrinoviride*	*hfb4*	PF06766	65	GPCTAGVTNNIPKCCGAGILDLLYLDCETPREVSSILNPLDAICARQGLQAKCCTLGIADLGVLC	6.7	0.6	4.3	0.5	Cell envelope	QLH01942.1

**A0A7D5R 5Z2**	*Trichoderma crassum*	*hfb4*	PF06766	65	GPCSSGVTNNVPKCCSSGILDLLYLDCKTPT QATSVLNPLSAVCGRVGLQAKCCTAGIANLG VLC	6.5	0.4	8.0	0.5	Cell envelope	QLH01897. 1
**A0A7D5 M0K7**	*Trichoderma cremeum*	*hfb4*	PF06766	65	SPCPGGVTNNIPKCCGAGVLDLLYLDCKTPT QVTSVLNPLSAVCGRVGLQAKCCTIGIADLG VLC	6.5	0.5	7.6	0.6	Cell envelope	QLH01862. 1
**A0A7D5Q Z76**	*Trichoderma epimyces*	*hfb4*	PF06766	65	GPCPSGVTNNIPKCCGSGILDLLYLDCETPT QVPSVHNPLSAICARVGLQAKCCTLGIADLG VLC	6.6	0.7	5.6	0.5	Cell envelope	QLH01911. 1
**A0A7D5R AG3**	*Trichoderma estonicum*	*hfb4*	PF06766	65	GACAGGVTNNVPKCCGAGILDLLYLDCKTPT QATSVLNPLSAVCGRVGLQAKCCTLGIAGLG VLC	6.4	0.5	8.0	0.8	Cell envelope	QLH01919. 1
**A0A7D5L Z69**	*Trichoderma fertile*	*hfb4*	PF06766	65	GPCPSGVTNNVPKCCGSGLLDLLYLDCKTPT QATSVLNPLSAVCGRVGLQAKCCTVGIAGLG VLC	6.5	0.6	8.0	0.6	Cell envelope	QLH01909. 1
**A0A7D5R 5H3**	*Trichoderma flagellatum*	*hfb4*	PF06766	65	GPCPSGVTNNIPKCCGAGVLDLLYLDCKTPT QATSVLNPLSAVCGRVGLQAKCCTAGIAGLG VLC	6.4	0.6	8.0	0.6	Cell envelope	QLH01956. 1
**A0A7D5R 0G3**	*Trichoderma gamsii*	*hfb4*	PF06766	65	GPCSSGVTNNIPKCCGAGVLDLLYLDCETPR QTSSVLNPLSNICAVRGLQAKCCTVGIAGLG VLC	6.6	0.5	7.6	0.5	Cell envelope	QLH01853. 1
**A0A1T3C WR1**	*Trichoderma guizhouense*	*hfb3*	PF06766	103	MQFFAVAALLFTAAFAAPSTEAPAGLARRTEA WCPPGLLYSNPLCCDTDVLGVADVDCVTPP EAPSKCKSFGSVCASIGRQPKCCAIPVAGLAL LCTDAIPQY	10.6	0.1	4.5	0.5	Cell envelope	AWT58087. 1
**A0A1T3C 949**	*Trichoderma guizhouense*	*hfb4*	PF06766	102	MKFSAIALFASLAIAAPATEATQEHQFDIYDGP CSGGVTNNIPKCCGAGLLDLLYLDCETPDEV TSPLNPLDAVCARMGLSAKCCTLGIADLGVL CQDALPQ	10.6	0.2	3.9	0.4	Cell envelope	AWT58088. 1
**A0A2U9Q 1K7**	*Trichoderma guizhouense*	*hfb5*	PF06766	97	MKCSFVATLLVAGVLAAPSSDVKAVKRQSQV CPTGLESNAQCCATDVLGIADLDCATPPNPF TDVTSFTAVCAAIGQRARCCAIPVLGQDLLCT SPV	9.9	0.2	4.7	0.6	Cell envelope	AWT58089. 1
**A0A1T3C CP4**	*Trichoderma guizhouense*	*hfb6*	PF06766	173	MKLLAITALFIAGALAVPAGSYPPPPPPPDYGD GGDNGGYSPTPSSSSGYGYVPPTPTLPPPPP PPSYDDGGDYEPPTDGDGDSSDGGDDGD DSGDSGDGGDGERSGSNLCPAVVSGIAKC	17.1	0.2	3.7	-0.4	Cell envelope	OPB38878. 1

Journal of Fungi

MDPI

					CSASVLGLLNLDCVSPSKEPKDRKDFEKICSSIGKQATCCTLGLLGLGVLCGQ						
**A0A1T3CBS5**	*Trichoderma guizhouense*	*hfb8*	PF06766	88	MKFFAAAALFATSAMAAVCPDGGLFSNPLCCSSILLEAVGLDCTTPTAPVVTAGLFQANCASIGKQPACCVAPLAGQGILCNNPAGTH	8.7	0.1	5.5	0.8	Cell envelope	AWT58091.1
**A0A2U9Q1M5**	*Trichoderma guizhouense*	*hfb9a*	-	152	MYASVILYALVTLGGVMASPAQEPGVEPRAIERRVPTMADIPFPRFPTRNYNHNDGHDGKGHDGKDHDSKDHGGKDDDGKGCSSDTNTQVNACSGGNPYCCSSNGKGGHACKNTTACEETIICCNNNNGFQICIGDIDFNVPITINIYEDDD	16.3	0.4	4.6	-0.7	Cell envelope	AWT58092.1
**A0A1T3CTV0**	*Trichoderma guizhouense*	*hfb9b*	-	134	MYASIIFYALVALNGVMASPVQGPEVEPRAIERRVPSMGDIPFPKFPARNKNSNKMGKGNSGSSDCSGSGTNTQVNACSAGSPYCCSSDGNGGQTCSNTTACTETIICCNNNNGFQICIGDLDFNVPITINVYE	14.0	0.2	4.6	-0.2	Cell envelope	AWT58093.1
**A0A5P8PB18**	*Trichoderma guizhouense*	*hfb10*	PF06766	136	MKFLTVAAVFFTAVLAAPGNYPPPPPPTYAPPPPSYTLPAGGNGNGNGNGHGNGNGNGNGNGNTSGGAVCPEGLYSNPQCCSALVLGIVGLDCSTPTSVQSPSAFKNGCAAKGAQAVCCVLPVAGQDVLCQTAIGA	13.2	0.1	7.0	0.1	Cell envelope	QFR52899.1 [85]
**A0A1T3CBM1**	*Trichoderma guizhouense*	*hfb16*	-	277	MLFRTATSLALLACAASAAAEPKPYRLAMMPVLGQSLARRSTNGYQPELSQCHDGDTCAEACGSEYTECASSGKETHCYNPSVKQSCCADGSGNSCDEGYYCTHDHALKTWCCPENMDLAACAAAYSVAGGLESATPTSTSTSSTSTSTTSTTVVTTSAAPTTTSTTEAPTTTAAPTTTKAPTTTKAPATTVTKVDEETTTVPCTTSAGFTSAYTGFNSTVTSSVPNQPHQSKTPLPPVASISTGAPQPPQPSTISGAATTSASALLLLAAGVIALL	28.0	0.3	4.9	-0.1	-	AWT58096.1
**A0A219TTW8**	*Trichoderma harzianum*	*ThHyd1*	PF06766	93	MKFFAVAALFVASAMAGPMGSEGCPGGLTGTVPLCCATNVLNIADLDCSTPTIPVPNVGIFQAHCASKGKQPVCCTLPVAGQGLLCNKPTGAQ	9.3	0.1	7.6	0.6	Cell membrane, localizes to the plasma membrane in maize roots	ANU06237.1

									during plant interactio n	
**A0A1B1R VB9**	*Trichoderma harzianum*	*ThHyd2*	PF06766	102	MKFSAIALFASLAIAAPATDAADATQFDIYDGP CSAGVTNNIPKCCGAGVLDLLYLDCETPQEV TSPLNPLHAVCARVGLSAKCCTLGIADLGVL CQDALPQ	10.4	0.2	4.0	0.5	Cell envelope	ANU06238. 1
**A3RJX1**	*Trichoderma harzianum*	*Hytra1*	PF06766	71	AVCPTGLFSNPLCCSTNVLDLVGVDCVTPTA AVPDGVFFQAHCASKGKQPLCCVAPVAKQG VLCQKPIGTQ	7.2	0.5	7.6	0.5	Cell envelope	ABN64104. 1
**P52755**	*Trichoderma harzianum*	*Qid3*	PF06766	143	MKFLTVAAVFFTAVLAAPGNYPPPPPPTYAPP PPTYTLPPNGNGGGNGNGNGNGNGGGNG NGNGNTNTGGSALCPAGLYSNPQSCATDVL GLADLDCAVPSTTPHDGPNFQSICVANGGK RARCCVLPVLGLGVLCQNPVGTN	14.0	0.2	7.0	-0.1	Secreted, cell wall	CAA50728. 1 [88]
**P79072**	*Trichoderma harzianum*	*Srh1*	PF06766	89	MQFSIVALFATGALASVSVCPNGLYSNPQCC GANVLGVAALDCHTPRVDVLTGPIFQAVCAA EGGKQPLCCVVPVAGQDLLCEEAQGTF	9.0	0.1	4.1	0.7	Cell envelope	CAA72539. 1 [91]
**A0A2N1L EB5**	*Trichoderma harzianum*	*Hfb1*	PF06766	111	MKFFAIAALLAAVAVASPLEDALNVGSEWWG DHDHHNDDHASLICPKGLYSNPQCCGSYLL GIIGLDCHIPNEKFYDSEDLRKTCTKTKESALC CVASASDEAILCQLPLA	12.0	0.3	4.6	0.1	Cell envelope	AWT58097. 1
**A0A2N1L QG4**	*Trichoderma harzianum*	*Hfb3*	PF06766	100	MQFLAVAALLFTAAFAAPSTEAPALVRRANAY CPNGLYAVPQCCDVDVLGVAALDCRSPPVT PTKCNSFNNICAAIGLQPKCCVLPVAGQALL CTNPVPQ	10.3	0.1	7.6	0.6	Cell envelope	AWT58098. 1
**A0A2N1L TE2**	*Trichoderma harzianum*	*Hfb4*	PF06766	102	MKFSAIALFASLAIAAPATEATQEHQFDIYDGP CSAGVTNNIPKCCGAGILDLLYLNCETPKEVT SPLNPLDDVCARVGLSAKCCTLGIADLGVLC QDALPQ	10.6	0.3	4.1	0.4	Cell envelope	AWT58099. 1
**A0A2N1L YT2**	*Trichoderma harzianum*	*Hfb5*	PF06766	94	MKSFFVATLLVAGVLAAPSSDVKRQTSPCPAG LEATPQCCATDVLGVADLDCANPPNPFTDAA SFTAVCAAIGQRARCCAIPVAGQDLLCTSPV	9.5	0.2	4.4	0.5	Cell envelope	AWT58100. 1
**A0A2U9Q 1P5**	*Trichoderma harzianum*	*Hfb6*	PF06766	153	MKLLAITALFIAGALAVPAGSYPPPPPPDYGGY SPVPSSSGDDYGYVPPTPTPPPPPPPPPPPP SYDDGGDYEPPTDGDGDSSDGGDDGDDS	15.6	0.2	3.6	-0.7	Cell envelope	AWT58101. 1

					DDGSDGGDGERSQTNLCPAVISGIAKCCSTNVLDLLNLDCVSPNKEPKDRKDFEKIFAADS						
**A0A0B5H3H0**	*Trichoderma harzianum*	*Hfb7*	PF06766	93	MKFFAVAALFVASAMASPMGSEGCPGGLTNTVPLCCATNVLGVATLDCSTPTVPVPNVGIFQAHCASKGKQPVCCTVPVAGLGLLCQKPTGAQ	9.2	0.2	8.0	0.7	Cell envelope	AJF36076.1 [38]
**A0A2N1L9M2**	*Trichoderma harzianum*	*Hfb9a*	-	167	MYASVILYALVTLGGAMASPAQEPGVEPRAIERRVPTMADIPFPRFPTRNYNHNDGGKGGKGGKDGKDGKGHDGKGHDGKDHDGKDHGGKDDDGKGCSSDTNTQVNACSGGNPYCCSSNGKGGHACKNTTACEETIICCNNNNGFQICIGDIDFNVPITINIYEDDD	17.5	0.3	5.2	-0.8	-	AWT58103.1
**A0A2U9Q1X0**	*Trichoderma harzianum*	*Hfb9b*	-	131	MYASIIFYALVALNGVMASPVQGPEVEPRAIERRVPSMGDIPFPKFPARNKNSNKMGKGSSDCSGSGTNTQVNACSAGSPYCCSSDGNGGQTCSNTTACTETIICCNNNNGFQICIGDLDFNVPITINVYE	13.8	0.2	4.6	-0.2	-	AWT58104.1
**A0A5P8PAX6**	*Trichoderma harzianum*	*Hfb10*	PF06766	130	MKFLTVATLLFTAVLAAPSKLLTPPPPPPTYTLPAGGNGNGNGNGHGNGNGNGNTNDAPVCPTGLYSNPQCCSTLVLGIVGLDCSTRNIATSVHDPSAFKNACAAKGAQAVCCVLPVAGQDVLCQTAIGA	12.9	0.2	7.7	0.2	Cell envelope	QFR52898.1
**A0A2U9Q1V8**	*Trichoderma harzianum*	*Hfb15*	PF00188	293	MRLLLFIYTLITFFKFSSSTVVVTITAAPAIPSGEPSYSNSASFTSAILNSTNTYRREYNATSLYWNTTLEKFATAYLQSDTTCRFAHSGGPYGENIAIGYANATATVEAWGNEDEKYNFNNPGFTEETGHFSQLVWKATRGVGCGRKLCGTRGWFVVCEYWPRGNVGGQYGEEVDRRVGGGGAGGGATRLSGGVVAAIAVVYTTALLALAATAIAVPTTSSNKGNGGGKDNNGGESTCNTNQVAVCCNGLLGGILCNIGILGNSCSSGSYCCDSSAEQGGLINLNLLNCVAL	30.6	0.3	6.5	0	Extracellular region	AWT58106.1
**A0A2U9Q1Z7**	*Trichoderma harzianum*	*Hfb16*	-	283	MLFRTATSVALLACAVSVSAEPKPYRLAMMPVLGQSLARRSTNGYQPELSQCHDGDTCAEACGSEYTECASSGKETHCYNPSVKQSCCADGSGNSCDEGYYCTHDHALKTWCCPENMDLAACAAAYSVAGGLESATPTSTSTSSTSTSTSTSTSTTSTTVVTTSAAPTTTSTTEAPTTTAAPTTTKAPTT	28.6	0.4	4.8	-0.2	-	AWT58107.1

					TKAPATTVTKVDEETTTVPCTTSAGFTSAYTGFNSTVTSSVPNQPNQSKTPLPPVASISTGAPQPPQPSTISGAATTSASALLLLAAGVIALL						
**A0A2U9Q1Q3**	*Trichoderma harzianum*	*Hfb17*	PF06766	92	MNMKLLFLSILAVSVFAFDPCPTNNRNRPACCSVDVLKVADLDCETPPGVEQFQNGMEFKQACQTLGQAPRCCTLLLTGEGIGCDDFVKLDK	10.0	0.4	4.4	0.1	Cell envelope	AWT58108.1
**A0A7D5R0V7**	*Trichoderma helicum*	*hfb4*	PF06766	65	GPCPSGVTNNVPKCCGAGVLDLLYLDCKTPTQATSVLNPLSAVCGRVGLQAKCCTVGIAGLGVLC	6.4	0.6	8.0	0.6	Cell envelope	QLH01933.1 [85]
**A0A7D5QY69**	*Trichoderma inhamatum*	*hfb4*	PF06766	65	GPCSAGVTNNIPKCCGAGVLDLLYLDCETPQEVTSPLNPLDAVCARVGLSAKCCTLGIADLGVLC	6.5	0.6	4.0	0.6	Cell envelope	QLH01872.1 [85]
**A0A7D5R4L5**	*Trichoderma konilangbra*	*hfb4*	PF06766	65	GPCPSGVTNNIPKCCGAGVLELLYLDCKTPTQATSVLNPLSAVCGRVGLQAKCCTAGIAGLGVLC	6.4	0.6	8.0	0.6	Cell envelope	QLH01958.1 [85]
**A0A7D5R4A7**	*Trichoderma koningiopsis*	*hfb4*	PF06766	65	GPCSAGVTNNIPQCCGAGILDLLYLDCQTPTAVSSVLNPLSAICATKGLQAKCCTVGIAGLGVLC	6.4	0.6	5.9	0.8	Cell envelope	QLH01858.1 [85]
**A0A7D5AKE8**	*Trichoderma lixii*	*hfb10*	PF06766	148	MKFLTVAAVFFTAALAAPGNYPPPPPPTYAPPPPTYTLPPNGNGGGNGNGNGNGNGNGNGNGNGGGNGNGNTNTGGSPLCPSGLYSNPQCCATDVLGLADLDCAVPSTTPHDGPNFQSICAANGKRARCCVLPVVGLGVLCENPVGTN	14.5	0.2	5.6	-0.3	Cell envelope	QKV26103.1 [85]
**A0A7D5QWA0**	*Trichoderma longipile*	*hfb4*	PF06766	65	GPCAGGVTNNVPKCCGAGILDLLYLDCKTPTQATSVLNPLSAVCGRVGLQAKCCTLGIAGLGVLC	6.4	0.5	8.0	0.7	Cell envelope	QLH01930.1 [85]
**A0A7D5LZ71**	*Trichoderma novae-zelandiae*	*Hfb4*	PF06766	65	GPCPSGVTNNIPKCCGSGILDLLYLDCKTPTQATSVLNPLSAVCGRSGLQAKCCTVGIADLGVLC	6.5	0.5	7.6	0.5	Cell envelope	QLH01945.1 [85]
**A0A7D5QW73**	*Trichoderma oblongisporum*	*hfb4*	PF06766	65	GPCPAGVTNNVPKCCGAGILDLLYLDCKTPTQATSVLNPLSAVCGRVGLQAKCCTLGIAGLGVLC	6.4	0.5	8.0	0.7	Cell envelope	QLH01910.1 [85]
**A0A7D5M0V7**	*Trichoderma orientale*	*hfb4*	PF06766	65	GPCSDGVTNNIPKCCGAGILDLLYLDCKTPTQVTSILNPLSAVCGRVGLQAKCCTVGIAGLGVLC	6.5	0.6	7.6	0.7	Cell envelope	QLH01964.1 [85]
**A0A7D5QW48**	*Trichoderma parapiluliferum*	*hfb4*	PF06766	65	GPCSSGVTNNIPQCCGAGILDLLYLDCKTPTQATSVLNPLSAVCGRVGLQAKCCTLGIAGLGVLC	6.5	0.5	7.6	0.7	Cell envelope	QLH01890.1 [85]

**A0A7D5M0R2**	*Trichoderma patella*	*hfb4*	PF06766	65	GPCTSGVTNSIPKCCGSGILDLLYLDCETPKQVSSILNPLSAICGRVGLQAKCCTVGIADLGVLC	6.6	0.6	6.1	0.6	Cell envelope	QLH01944.1 [85]
**A0A7D5R586**	*Trichoderma petersenii*	*hfb4*	PF06766	65	GPCSAGLTNNIPQCCGAGVLDLLYLDCETPREISSVLNPLRNICAIRGLQAKCCTVGIAGLGVLC	6.7	0.5	6.1	0.6	Cell envelope	QLH01856.1 [85]
**A0A7D5LXN4**	*Trichoderma pezizoides*	*hfb4*	PF06766	65	GPCAAGVTNNIPQCCGAGLLNILYLDCKTPTQATSVLNPLSAVCGRVGLQAKCCTLGIAGLGVLC	6.4	0.4	8.0	0.7	Cell envelope	QLH01888.1 [85]
**A0A7D5LY54**	*Trichoderma phyllostachydis*	*hfb4*	PF06766	65	GPCPGGVTNNVPKCCGSGILDLLYLDCKTPTQATSVLNPLSAVCGRVGLQAKCCTVGIANLGVLC	6.5	0.5	8.0	0.6	Cell envelope	QLH01920.1 [85]
**A0A7D5QY89**	*Trichoderma pleuroti*	*hfb4*	PF06766	65	GPCPAGVTNNIPKCCGSGILDLLYLDCKTPTQVSSILNPLSAICGRVGLQAKCCTAGIAGLGVLC	6.5	0.5	8.0	0.7	Cell envelope	QLH01892.1 [85]
**A0A7D5LZ41**	*Trichoderma polysporum*	*hfb4*	PF06766	65	GPCASGVTNNIPKCCGAGILDLLYLDCKTPTQATSVLNPLSAVCGRVGLQAKCCTVGIAGLGVLC	6.4	0.5	8.0	0.7	Cell envelope	QLH01889.1 [85]
**A0A7D5RAC0**	*Trichoderma pseudonigrovirens*	*hfb4*	PF06766	65	GPCPAGVTNNIPKCCGTGVLGLLYFDCKTPTQSTSAHNPLSAICGRVGLQAKCCTLGIASLGVLC	6.5	0.5	8.2	0.5	Cell envelope	QLH01859.1 [85]
**A0A7D5QZ93**	*Trichoderma pubescens*	*hfb4*	PF06766	65	GPCAGGVTNNVPKCCGAGILDLLYLDCKTPTQATSVLNPLSAVCGRVGLQAKCCTLGIAGLGVLC	6.4	0.5	8.0	0.7	Cell envelope	QLH01931.1 [85]
**P52754**	*Trichoderma reesei*	*hfb1*	PF06766	97	MKFFAIAALFAAAAVAQPLEDRSNGNGNVCPPGLFSNPQCCATQVLGLIGLDCKVPSQNVYDGTDFRNVCAKTGAQPLCCVAPVAGQALLCQTAVGA	9.9	0.2	6.1	0.4	Secreted in aqueous environment, cell wall	CAA92208.1 [39]
**P79073**	*Trichoderma reesei*	*hfb2*	PF06766	86	MQFFAVALFATSALAAVCPTGLFSNPLCCATNVLDLIGVDCKTPTIAVDTGAIFQAHCASKGSKPLCCVAPVADQALLCQKAIGTF	8.8	0.1	7.0	0.9	Secreted, cell wall, spore wall	CAA72636.1 [97]
**G0RVE5**	*Trichoderma reesei*	*Trhfb3*	PF06766	102	MQFLAVAALLFTTALAAPSSDVNGIIRRANAFCPEGLLYTNPLCCDLDVLGVADVDCVVPPAKPSSCKSFGSVCASIGRKPRCCAVPVAGVALLCTDPIPAI	10.4	0.1	6.2	0.8	Secreted, cell wall, cytoplasm,	EGR44794.1 [40]

										uniformly detected in the cytoplasm of the conidia and hyphae	
**A0A7D5LZ99**	*Trichoderma reesei*	*hfb4*	PF06766	65	GPCTAGVTNNIPMCCGSGILDLLYLDCETPTQATSVLNPLSAVCGRVGLQAKCCTLGIADLGVLC	6.5	0.3	4.1	0.7	Cell envelope	QLH01947.1 [85]
**A0A7D5QW02**	*Trichoderma rogersonii*	*hfb4*	PF06766	65	GPCSSGVTNNIPQCCGAGILDILYLDCETPRADSSILNPLRNICAARGLQAKCCTVGIAGLGVLC	6.6	0.4	6.1	0.5	Cell envelope	QLH01850.1 [85]
**A0A7D5QXW3**	*Trichoderma saturnisporum*	*hfb4*	PF06766	65	GPCSAGATNSIPKCCGGGLLDLLYLDCKTPTQATSVLNPLSAVCGRVGLQAKCCTAGIAGLGVLC	6.3	0.5	8.0	0.6	Cell envelope	QLH01954.1 [85]
**A0A8G0PA18**	*Trichoderma simmonsii*	*hfb4*	PF06766	101	MKFTAVALFASLAIAAPATETSLEERQVGGGPCPSGVTNNIPKCCGSGILDLLYLDCETPTQVTSVLNPLSAVCARVGLQAKCCTVGIADLGVLCQDALPQ	10.3	0.5	4.3	0.5	Cell envelope	QYS95010.1 [41]
**A0A8G0PA33**	*Trichoderma simmonsii*	*hfb6*	PF06766	173	MKLLAITALFIAGALAVPAGSYPPPPPPPDYGDGGDNGGYSPTPSSSSGYGYVPPTPTPPPPPPPPSYDDGGDYEPPTDGDGDSSDGGDDGDDSGDSGDGGDGERSGSNLCPAVVSGIAKCCSASVLGLLNLDCVSPSKEPKDRKDFEKICSSIGKQATCCTLGLLGLGVLCGQ	17.1	0.2	3.7	-0.4	Cell envelope	QYS92912.1 [41]
**A0A8G0L6M6**	*Trichoderma simmonsii*	*hfb10*	PF06766	140	MKFLTVAAVFFTAVLAAPGNYPPPPPPPTYAPPPPAYTLPPNGNGNGNGNGNGNGHGSGNGNGNGNTNTSGGSVCPAGLYSNPQCCSTLVLGIVGLDCSTPTNVHDPSAFTKGCATAGKQAVCCVLPVVSDFSEDKFSSLY	13.9	0.2	6.5	-0.1	Cell envelope	QYS96712.1 [41]
**A0A8G0LIV3**	*Trichoderma simmonsii*	*hfb15*	-	93	MKYTTALLALAATAIAMPTTGGNKGNGDGKDNNGGESTCNTNQVAVCCNGLLGGILCNIDILGNSCSSGSYCCDSSAEQGGLINLNLLNCVAL	9.2	0.2	4.0	0.2	-	QYT03133.1 [41]
**A0A8G0P9S6**	*Trichoderma simmonsii*	*hfb1*	PF06766	93	MKFFAVAALFVASAMAGPMGSEGCPGGLTGTVPLCCATNVLDIADLDCSTPTIPVPNVGIFQ	9.3	0.1	7.0	0.6	Cell envelope	QYS93361.1 [41]

					AHCASKGKQPVCCTLPIAGQGLLCNKPTGAQ						
-	*Trichoderma simmonsii*	*hfb7*	-	-	Automatic annotation (Putative)	-	-	-	-	-	[93]
**A0A7D5M0G6**	*Trichoderma sinuosum*	*hfb4*	PF06766	65	GPCPAGVTNNIPKCCGSGILDLLYLDCKTPTQATSVLNPLSAVCGRVGLQAKCCTVGIADLGVLC	6.5	0.5	7.6	0.6	Cell envelope	QLH01864.1 [85]
**A0A7D5R4F3**	*Trichoderma spirale*	*hfb4*	PF06766	65	GPCPSGVTNNVPKCCGAGILDLLYLDCKTPTQATSVLNPLSAVCGRVGLQAKCCTVGIAGLGVLC	6.4	0.5	8.0	0.7	Cell envelope	QLH01918.1 [85]
**A0A7D5LY68**	*Trichoderma stramineum*	*hfb4*	PF06766	65	GPCPSGVTNNIPKCCGAGILDLLYLDCKTPTQATSVLNPLSAVCGRVGLQAKCCTAGIAGLGVLC	6.4	0.5	8.0	0.6	Cell envelope	QLH01940.1 [85]
**A0A7D5LXJ7**	*Trichoderma strictipile*	*hfb4*	PF06766	65	GPCSSGVTNNIPKCCGSGILDILYLDCKTPTQATSALNPLSAVCGRVGLQAKCCTLGIADLGVLC	6.5	0.6	7.6	0.5	Cell envelope	QLH01887.1 [85]
**A0A7D5LY71**	*Trichoderma strictipile*	*hfb4*	PF06766	65	GPCAGGVTNNQPKCCGAGILDLLYLDCKTPTQATSVLNPLSAVCGRVGLQAKCCTVGIAGLGVLC	6.4	0.5	8.0	0.6	Cell envelope	QLH01906.1 [85]
**A0A7D5QY30**	*Trichoderma strigosum*	*hfb4*	PF06766	65	GPCSAGVTNNIPQCCGAGILDLLYLDCKTPTQVSSALNPLSAICGKVGLQAKCCTVGIAGLGVLC	6.4	0.6	7.6	0.7	Cell envelope	QLH01832.1 [85]
**A0A7D5M1Q7**	*Trichoderma surrotundum*	*hfb4*	PF06766	65	GPCSSGVTNNVPKCCGSGILDLLYLDCKTPTQATSVLNPLSAVCGRVGLQAKCCTVGIAGLGVLC	6.4	0.6	8.0	0.6	Cell envelope	QLH01863.1 [85]
**A0A7D5R5D6**	*Trichoderma tawa*	*hfb4*	PF06766	65	GPCPSGVTNNIPKCCGSGILDLLYLDCKTPTQVTSVLNPLSAVCGRVGLQAKCCTVGIADLGVLC	6.6	0.6	7.6	0.6	Cell envelope	QLH01916.1 [85]
**A0A7D5QXV5**	*Trichoderma tomentosum*	*hfb4*	PF06766	65	GPCGSGVTNNNPKCCGAGILDLLYLDCKTPTQATSVLNPLSAVCGRVGLQAKCCTVGIAGLGVLC	6.4	0.5	8.0	0.6	Cell envelope	QLH01934.1 [85]
**A0A7D5R624**	*Trichoderma velutinum*	*hfb4*	PF06766	65	GPCASGVTNNVPKCCGSGILDLLYLDCKTPTQATSVLNPLSAVCGRVGLQAKCCTVGIAGLGVLC	6.4	0.5	8.0	0.7	Cell envelope	QLH01937.1 [85]
**Q0QBT2**	*Trichoderma virens*	*TVH1*	PF06766	45	TPPVAPSKCKSFGSVCAAIGLQPKCCVLPVAGVAVLCTDPLPPAF	4.5	0.1	8.2	0.8	Cell envelope	ABE60727.1 [42]

**A0A7D5LY27**	*Trichoderma virens*	*HFB4*	PF06766	65	GPCSSGVTNNVPKCCGTGVLDLLYLDCKTPTQATSVLNPLSAVCGRVGLQAKCCTAGIGSLGVLC	6.4	0.5	8.0	0.5	Cell envelope	QLH01900.1 [85]
**A0A7D5QXN7**	*Trichoderma viridescens*	*hfb4*	PF06766	65	GPCSSGVTNNVPKCCGAGILDLLYLDCETPRAVSSVLNPLSNICAARGLQAKCCTVGIAGLGVLC	6.5	0.4	7.6	0.6	Cell envelope	QLH01854.1 [85]
**A0A7D5LYX4**	*Trichoderma voglmayrii*	*hfb4*	PF06766	65	GPCSNGLTNNIPQCCGAGILDLLYLDCESPNQVSSILNPLNAICSRVGLQAKCCTVGIAGLGVLC	6.6	0.6	4.4	0.6	Cell envelope	QLH01829.1 [85]
**G2XFX7**	*Verticillium dahliae*	-	PF06766	96	MQFTIVAALFASVAMAAPATLHARATVCPTGLLYGVAQCCATSVLGVADLDCSVPSSTPSDGADLKRICAESGAAAMCCSIPLAGQGVLCTPVIG	9.3	0.1	4.6	0.9	cell wall	-
**Q9HFD2**	*Xanthoria ectaneoides*	*H1*	PF01185	83	TTPGQAVQNQCSQGQTAKCCNSLSKAVANLIPIQVGLNCVSLDLISVLPIGKQCTQSQALACCSSGQQTGLVNLGNVCVPVSL	8.4	0.5	8.0	0.4	Secreted, cell wall	CAC08469.1 [100]
**Q9HFD1**	*Xanthoria parietina*	*H1*	PF01185	83	TTPGQSIQNQCSQGQTAKCCNSLSKAVANLIPIQIGLNCVSLDLISVLPIGKQCTQSQALACCSSGQQTGLVNLGNVCVPVSL	8.5	0.5	8.0	0.3	Secreted, cell wall	CAC08470.1 [100]
**Q96VD0**	*Xanthoria parietina*	-	PF01185	110	MQFKNIIAFVSLAVMASAAPAENLVERTTPGQSIQNQCSQGQTAKCCNSLSKAVANLIPIQIGLNCVSLDLISVLPIGKQCTQSQALACCSSGQQTGLVNLGNVCVPVSL	11.4	0.2	8.0	0.4	cell wall	CAC43386.1 [99]

Journal of Fungi

MDPI

Table S2. Biophysical Properties of Hydrophobins from Basidiomycota. Mass (Da), solubility, isoelectric point (pI), and GRAVY index were predicted using ExPASy ProtParam (https://web.expasy.org/protparam/). Protein solubility was evaluated using Protein-Sol (https://protein-sol.manchester.ac.uk/). Subcellular localization was based on UniProt annotations; for those without this description (D), it was used DeepLoc (https://services.healthtech.dtu.dk/services/DeepLoc-2.0/).

Entry (Uniprot)	Organism	Gene Name	Pfam	Length	Protein Sequence	Mass (Da)	Solubility	Pi	Gravy	Location	Protein n°
**P49072**	*Agaricus bisporus*	*hypA*	PF01185	112	MISRVLVAALVALPALVTATPAPGKPKASSQC DVGEIHCCDTQQTPDHTSAAASGLLGVPINL GAFLGFDCTPISVLGVGGNNCAAQPVCCTG NQFTALINALDCSPVNVNL	11.2	0.2	4.7	0.5	Secreted, cell wall	CAA61530. 1 [210]
**O13300**	*Agaricus bisporus*	*hypC*	PF01185	119	MFARISTIITTLFFAMLAAATAVPRTDPPPATGS QCTAVGGDVNCCNSVQDASNPIVGLLAGLL GIVLGPIQGLVGLTCNPISVIGGGNSCSSQTV CCTGNNFSGGLLVIGCSPINIDL	11.8	0.1	4.0	0.8	Secreted, cell wall	CAA74939. 1 [248]
**O43122**	*Agaricus bisporus*	*hypB*	PF01185	119	MVSTFITVAKTLLVALLFVNINIVVGTATTGKHC STGPIECCKQVMDSKSPQATELLTKNGLGLG VLAGVKGLVGANCSPITAIGIGSGSQCSGQT VCCQNNNFNGVVAIGCTPINANV	11.9	0.2	8.2	0.6	Secreted, cell wall	CAA75893. 1 [189]
**Q9HGX0**	*Agaricus bisporus*	*abh3*	PF01185	119	MFAGMSTIITILLFAMLAAASAVPRTDPPPARG SQCTAVGGDVNCCNSVQDASNPIVGLLAGL LGIVLGPIQGLVGLTCNPISIIGGGNSCSSQTV CCTGNNFSGGLLVIGCSPINIDL	11.7	0.1	4.0	0.8	Secreted, cell wall	CAC03465. 1 [173]
**Q9Y8F0**	*Agrocybe aegerita*	*Aa-pri2*	PF01185	123	MVAIKSLAILALPVMAMASPLVPRTDSPSQC NNGSLQCCNSSMTQDRGNLQIAQGVLGGL LGGLLGLGGLLDLVDLNALIGVQCSPISIVGN ANTCTQQTVCCSNNNFNGLIALGCTPININL	12.5	0.3	4.5	0.6	Secreted, cell wall	AAD41222. 1 [211]
**P78601**	*Coprinopsis cinerea*	*coH1*	PF01185	113	MQFKFLSTVALATLAVAAPAPTDPTPIPPSQC NTGPIQCCNTVTQASNPVAGLLLGLLGIVLQ DLNVLVGLTCSPISIIGLPGNSCNAQPVCCQ NNNFNGLIAIGCTPININL	11.5	0.3	4.1	0.6	Secreted, cell wall	CAA71652. 1 [203]
**P78602**	*Coprinopsis cinerea*	*coH2*	PF01185	110	MQFKFLTTVALATLAVAVPTDPPPTNQCNAP NNLECCNSVQAPTNSGLIGTLLGLLNISVGDI TGLVGLTCNPISLIGGGNSCNAQTVCCQNNY FGGLISIGCTPIIIDV	11.2	0.3	3.7	0.6	Secreted, cell wall	CAA71653. 1 [203]
**A8P9W4**	*Coprinopsis cinerea*	*hyd1*	PF01185	118	MFARLSTALLAFTLATAVVAAPGGRPSEVEYE QCNGGEIQCCNSYQKADSLDYNTSKLLGLL	12.1	0.3	6.2	0.3	Secreted, cell wall	EAU82003. 1 [174]

Journal of Fungi

MDPI

					NIDVKQITAGVGLTCTGINAVGIGGGSSCTQQKVCCTNNKFNGVVALGCSPINVSL						
**A8PA37**	*Coprinopsis cinerea*	*hyd2*	PF01185	135	MFARLTSTLFALAAVSAVFAAPGATTEQCNGGEVQCCNSVQDANNLDSSVKKIITGLLHLDLKQITGQVGVTCTSVNVLGIGGGSSWYGFAFSYSYILASDWAIGVCSTQQKVCCTNNSFHGLIALGCTPINVSV	14	0.2	6.5	0.5	Secreted, cell wall	EAU81875.2 [174]
**D6RQ66**	*Coprinopsis cinerea*	*hyd3*	PF01185	162	MAQRPSVPIAIIGNYISRRRQVSLEEAEALAQGLKCRFLGEVHAIRDGPYSYADGTLESILNLYRDAFFGDFDSETGEHRKEKEFKGEVLSSGLKDIAKQKRHKLANIFMNPKPPKYRSSTSLGGIATGCSPRSVVGVGGGNRCAHRPVCCTDNKFSECLPL	17.8	0.3	9.1	-0.4	Secreted, cell wall	EFI26839.1 [174]
**Q8WZJ3**	*Dictyonema glabratum*	*H1*	PF01185	130	MFARTSVFIILALTASFVAAVPTTPKPPPPKPLPPKPSPPPSAPVIQCNTGPVQCCDSVQNSNDKEIANLLGFLGIVTGGDEVPIGVTCSPISVAGGTGSNSCTAQPVCCQNNTFNGVVAIGCNPININK	13.2	0.1	7.6	0.2	Secreted, cell wall	CAC86002.1 [212]
**Q8WZJ2**	*Dictyonema glabratum*	*H2*	PF01185	115	MLIRVTAFTILALTSSFVGALPSPAAATIPASQCNTGSLQCCQTLSNSSNSGVTTLSGLLGIVIPANVPVGLTCNPISLLVGIGGNSCSAQPVCCQGNNFNGLIVLGCTPVNLNL	11.4	0.1	7.6	0.8	Secreted, cell wall	CAC86005.1 [212]
**Q8WZJ1**	*Dictyonema glabratum*	*H3*	PF01185	118	MLIRITAFTVLALTTGSFVAALPSPALATTPIPASQCNTGPVQCCQSISSSSNSGTASLLGPLGLVLSGLNIPIGVTCTPISLLGIGGNSCSANPVCCQNNNFNGVVAVGCTPVNLSL	11.6	0.1	7.6	0.8	Secreted, cell wall	CAC86006.1 [212]
**Q9HDD5**	*Flammulina velutipes*	*fvh1*	PF01185	113	MVSFRAFTVAASLFATLAAATPLDTALPRAADQCNVSNQQCCNSVQQASSGPAALILGLLGVVLQDVNVLVGLDCSPITVIGGGNGGCNASPVCCENNSFGSLISIGCVPISI	11.2	0.2	*3.9*	0.7	Secreted, cell wall	BAB17622.1 [196]
**Q75T84**	*Flammulina velutipes*	*Hyd-1*	PF01185	121	MRLSISKLSVVMLALPILASASVLPRTGSTGASCGSTTSGQAQCCNSTQSNTTPLPFPLSVITGLLGIDLGAITGLVGLTCNPITVLGLGGTNCNSQAVCCTGNTFNGLINLGCSPLNVAL	12	0.1	*8.0*	0.7	Secreted, cell wall	ANH11479.1 [214]
**A0A1I9QLC3**	*Flammulina velutipes*	*Hyd-2*	PF01185	105	MFARCAATILALALATLTAATPTGQCNVGNQQCCNTVQEASSDPAAGLLALLGVNVQDVTGLVGLTCNPITAIGGLNSNCDASPVCCENNSFGSLISIGCVPISL	10.4	0.2	*3.6*	0.7	Secreted, cell wall	AOV80982.1 [214]

**A0A1I9QLC4**	*Flammulina velutipes*	*Hyd-3*	PF01185	105	MFARCAATIFALALATLTAATPTGQCNVGNQQCCNTVQEASSDPAAGILALLGVNVQDVTGLVGLTCNPITGIGGLNSNCDASPVCCENNSFGSLISLGCVPISL	10.4	0.2	3.6	0.6	Secreted, cell wall	AOV80983.1 [214]
**A0A1I9QLC5**	*Flammulina velutipes*	*Hyd-4*	PF01185	122	MFARSAATILAFVLVTLTAATPAYYAPGTKTTSVDKAVPAGQCNVGNQQCCNTVQEASSNPVAGLLGLLGVNVQDVTGLVGLTCNPITGIGGLNSNCDASPVCCENNSFGSLISIGCIPISL	12.2	0.2	4.2	0.5	Secreted, cell wall	AOV80984.1 [214]
**A0A1I9QLC6**	*Flammulina velutipes*	*Hyd-5*	PF01185	112	MFSAAISVFVLATLTAATPMARGIIPAPPAGQCNVGTQQCCNSVQDASSDPAAGLLALLGINVQDVTGQVGLTCNPITVIGGVNSNCDASPVCCENNSFGSLISLGCVPVSL	11.1	0.1	3.6	0.7	Secreted, cell wall	AOV80985.1 [214]
**A0A1I9QLC7**	*Flammulina velutipes*	*Hyd-6*	PF01185	112	MFSAAISVFVLATLTAATPMARGIVPAPPAGQCNVGTQQCCNTVQDASSDPAAGILALLGINVQDVTGQVGLTCNPITVIGGVNSNCDASPVCCENNSFGSLISLGCVPVSL	11.1	0.1	3.6	0.7	Secreted, cell wall	AOV80986.1 [214]
**A0A1I9QLC8**	*Flammulina velutipes*	*Hyd-7*	PF01185	112	MFARQATSVSAFLVLTLSLFAAASPLGPAPPTTSQCNVGNQQCCNTVQDSSSAPAAALLGLLGVVLQDVDVLVCSPITVIGGLNSACDASPVCCENNSFGSLISVGCIPISL	11.2	0.1	3.6	0.8	Secreted, cell wall	AOV80987.1 [214]
**A0A1I9QLC9**	*Flammulina velutipes*	*Hyd-8*	PF01185	128	MFALRTFAVLFASLSALAAATPLDARTNPTTTVTVTAPASTATVPAGQCNVSNQQCCNSVEKASSGPAALVLGLLGVVVQDVNVLVGLDCSPITVIGGGNGACNASPVCCENNSFGSLISIGCIPISL	12.6	0.2	4.2	0.7	Secreted, cell wall	AOV80988.1 [214]
**A0A1I9QLD0**	*Flammulina velutipes*	*Hyd-9*	PF01185	131	MFARRAISIFAFMLVALSIFAAATPLDARTNPTVTVTVTAPGSTATIPAGQCNVSNQQCCNSVQSASSTPVSVILGLLGIVLQDLNVLVGLTCSPITVIGGGNGGCNANPVCCQNNSFGSLISIGCIPISL	13.2	0.1	7.6	0.8	Secreted, cell wall	AOV80989.1 [214]
**A0A1I9QLD1**	*Flammulina velutipes*	*Hyd-10*	PF01185	118	MFARCVATIFASLLVTLAVASPEYYAPQGSTAPAPAGQCNVGNQQCCNTVQEASSDPVAGLLGLLGINVQDVTGLVGLTCNPITGIGGLNSGCDASPVCCENNSFGSLISIGCIPISL	11.8	0.2	3.5	0.6	Secreted, cell wall	AOV80990.1 [214]
**A0A9E8S4N7**	*Ganoderma lucidum*	*hyd1*	PF01185	145	MKFSYAIAAVVAAAASVQAVETNGQRMARGLKPLPPRNIHRRASRTNEARGATPSGTPSGGDYNCNTGSVQCCNQVSKANDTVISAILGLLGI	14.7	0.3	8.8	0.1	Secreted, cell wall	WAB46481.1

					GGVADDVLVGLKCSPLSVVGLGSGNSCSQRPVCCENNSKGGLVSIGCIPITL						
**A4LAR6**	*Grifola frondosa*	*hgfI*	PF01185	107	MFSKLAIFATAAFAVLAAATPVRRQQCTTGQLQCCESTSTANDPATSELLGLIGVVISDVDALVGLTCSPISVIGVGSGSACTANPVCCDSSPIGGLVSIGCVPVNV	10.6	0.1	4.0	0.8	Secreted, cell wall	ABB84487.1 [255]
**A0A8A5N547**	*Grifola frondosa*	*hgfII*	PF01185	108	MFSRIAAVSFLALPLLAAATPVLRRGDCNTGPIQCCQSTETAGSAAGSALLGLLGVVVQSVDVVLGLNCSPISVIGVGSGSACDASPVCCENNSVGGIISIGCVPIQL	10.6	0.2	4.2	0.9	Secreted, cell wall	QTF98737.1
**Q3HS86**	*Heterobasidion annosum*	*Hah1*	PF01185	139	MFFRISTVFVVALAAFAAASPAPWGAPPPTTTTHPPVTTTVTVTAPATTTTIPASQCNTGDAQCCNSVQSATAPAVTSLLGLLGIVLEDINVLVGLSCDPISVIGVGGGANCVQQPVCCENNNFNGLINIGCTPINIFL	14	0.1	3.9	0.7	Secreted, cell wall	ABA46363.1 [244]
**Q3HS87**	*Heterobasidion annosum*	*Hah2*	PF01185	122	MFARVSTLFAMFFLGLALMVSATPAALKPVARDTIPASQCNTGDLQCCNTVENADSPSAAALLGLLGVVVQGLDVLVGLTCTPITVIGVGSGANCVQQPVCCENNNFNGLINIGCTPVNLGL	12.4	0.3	4.0	0.8	Secreted, cell wall	ABA46362.1 [244]
**W4JT61**	*Heterobasidion irregulare*	*Hah1*	PF01185	139	MFFRISTVFVVALATFAAASPAPWGAPPPTTTTHPPVTTTVTVTAPATTTTIPASQCNTGDAQCCNSVQSATAPAVTSLLGLLGIVLEDINVLVGLSCDPISVIGVGGGANCVQQPVCCENNNFNGLINIGCTPINIFL	14	0.1	3.9	0.6	Secreted, cell wall	ETW76649.1 [175]
**W4JT56**	*Heterobasidion irregulare*	*Hah2*	PF01185	122	MFARVSTLFAMFFLGLALMVSATPAALKPVARDTIPASQCNTGDLQCCNTVENADSPSAAALLGLLGVVVQGLDVLVGLTCTPITVIGVGSGANCVQQPVCCENNNFNGLINIGCTPVNLGL	12.4	0.3	4.0	0.8	Secreted, cell wall	ETW76644.1 [175]
**W4KCJ5**	*Heterobasidion irregulare*	*hbf1*	PF01185	697	MLVQLSALSTVSVLGLALLASAAPSEPIPYGSSYGAAAPASASSLAPELYSSAAAPYSQPSPSSEVSAAVPAPYSSESPAPYKEYAPEVYSSAPAPYSSAAPEPSKTPEVYISAPVSYSSPAPVPSRAPEVYSAAPASYSSAAPAVYSSAPAPYSSVVSGAYASVPVSYSSAAPEPSKAPEAYSSAPASYSSAAPAPYKEYAPEVYSSAPAPYSSAAPEVYTSTPVSYSSAAPESSKGPEVYSSAAPAPYKEYAPEVYSSAPAPYSSAAPEVYTSTPVSYSSAAPEPSKGPEVYSSAAPAPYKEYAPEVYSSAPAPYSSVVPEV	71.6	0.4	4.3	-0.3	Secreted, cell wall	ETW83065.1 [175]

					YSSAPAAYSSVAPGPSKAPEAYSSAPAPYSSAAPTPYKEYAPEVYSSAPAPYSSAALYKAPEVYSSAAPAPYKEYAPEVYSSAPAPYSSVVPEVYSSAPAPSSSPAPEPYKAYVSEVYSSAPASYTSAAPEPYKASPSEVYSSVAMPYSSSTPQVYTSLAPEPYKAYAPEYNSTSAPYAKPTAEAYSSAPAPYSSAPPEVYSPVMASSSAYTIKVYSSANSLPAPAPTPYATWGPKPVAYKRGDYKPGEEPKVYKPEEKRPEVYKPEAKKTEVYPAPAPVYQAIEEHDVKQCNVGELRCCNTVETASATSASKELAVLGLVLQDLNTPIGLNCDALNTGGFGSGSNCAQQPVCCEKNEYGGLAIGCTPVNVVL						
**A0A0A7DLA4**	*Hypsizygus marmoreus*	*hyd*	PF01185	113	MFSKLAAVITISSVALSASAGHHRRGSQYQCNTGPVQCCNQLAESQSGAATAILALVGLSAQGLSGQVGAVCSPFTVVGVGSGANCATQPVCCDKNYIGGLVSLGCSPVNLCA	11.1	0.2	7.9	0.5	Secreted, cell wall	AIO05504.1
**B0CX61**	*Laccaria bicolor*	*LbH1*	PF01185	124	MKFSGLSTALVLLASGAAVSATQTVTVTTYAPVPTPPPASQCNTGALWCCNNTQAASGSIIAPLLALLGIVVDPVTALLGSGCTSINFLGIGTGGMCTAQPVCCQNNNFSGLVVIGCTPVTLGV	12.2	0.2	5.8	0.8	Secreted, cell wall	EDR13197.1 [176]
**B0D4Z0**	*Laccaria bicolor*	*LbH2*	PF01185	143	MKFTIIFTILSALFFAAVSAAPSLTNAQRLARGWSPNPPARRTTPVAAAKRGQPSGIHNSCNTGTMHCCNSVTSSNSRVASTLAGLLGIVLPHNTAVGITCSPLSAVGLGHGGTCTQQPVCCTGNNFSGLVVVGCSPININVT	14.5	0.0	9.4	0.3	Secreted, cell wall	EDR10424.1 [176]
**B0DG08**	*Laccaria bicolor*	*LbH3*	PF01185	163	MKFSRLSTALVLLAFGAAVSATQTVTATTYAPVPTPMPASLCPPPDLWCCNNTQPVNGGIIAPLLAILGIVVVPITTLCGAGCTSINYLGIGAGGMCTAQPVCCQYNNFSGVVVIGCIPVTLGISARRSAQYVFLKSFVTMLSCTSTPVSVYVFGIYDLHGHD	16.9	0.1	7.9	0.8	Secreted, cell wall	EDR06557.1 [176]
**B0DG11**	*Laccaria bicolor*	*LbH5*	PF01185	121	MKFSRLSTALVLLASGAAVSATQTVTYAPVQTPPPASQCNTGALWCCNNTQAANGSIIAPLLALLGIVVDPITALLGSGCTSINFLGVGTGGMCTAQPVCCQNNNFSGLVVIGCTPVTLGV	12	0.1	7.6	0.7	Secreted, cell wall	EDR06559.1 [176]
**B0DG18**	*Laccaria bicolor*	*LbH7*	PF01185	121	MTTATSFSTALVLLVLTIFVSVTVTTVRQPLSQWRTAVLWCCNETQAGQGNVVSALLVLLGIV	12.4	0.1	4.4	1.1	Secreted, cell wall	EDR06437.1 [176]

					ADLISALVGSGCTSINALGIGAGGMCTNNNF VSFLSFCEGCAPVTNGALSVVLCCWAM						
**B0DP21**	*Laccaria bicolor*	*LbH8*	PF01185	128	MFSQVLFVAAALVAFVAGSPVPGVENSCNTG SMQCCNQTFASDSNQANILASLLNINLSQFT GQVGTSCTPISALPIGQGGSCTQQPVCCDG NTFHGLIAVGCSPVNVNPQIFPISGQKMAIG AITVS	13	0.1	4.4	0.5	Secreted, cell wall	EDR03612.1 [176]
**B0DP52**	*Laccaria bicolor*	*LbH9*	PF01185	110	MFSKVLFVATALVAFVAASPVPDIENSCNTGS MQCCNQTFASNSKQANVLASLLNINLSQFT GQVGTSCTPISALAISRGGSCTQQPVCCDG NTFHGLIVVGCSPINVNL	11.3	0.1	7.0	0.5	Secreted, cell wall	EDR03553.1 [176]
**B0DP54**	*Laccaria bicolor*	*LbH10*	PF01185	110	MFSKVLFVAAALVSFVAASPVPDIENSCNTGS MQCCNQTFASDSKQANVLASLLNINLSQFT GQVGTSCTPISALAISQGGSCTQQPVCCDG NTFHGLIVVGCSPINVNL	11.3	0.2	4.5	0.5	Secreted, cell wall	EDR03555.1 [176]
**B0E003**	*Laccaria bicolor*	*LbH11*	PF01185	111	MFSKLALIVTSACALAAVAAPTSTGGSSCNAS GGTLQCCNSTESASNISGSISGLLGILGVNPA TLTGLIGLQCGISVVGGTSCSSQPVCCSGNN FNGLVVLGCSPVNVGL	10.5	0.2	5.9	0.8	Secreted, cell wall	EDQ99813.1 [176]
**B0E180**	*Laccaria bicolor*	*LbH12*	PF01185	109	MFSKVLFVAATLATFVAATPVPDGVSNSCNTG SLQCCNQTFSSTSGEANLLGALLNLNLGQLT GQIGLSCTPISVIGLGQGASCTQQPVCCSGN TFNGLINVGCTPISL	10.9	0.3	4.2	0.6	Secreted, cell wall	EDQ99412.1 [176]
**B0E182**	*Laccaria bicolor*	*LbH13*	PF01185	109	MFSKVLLVAATLVTFVAATPVPGGVDNSCNT GTLQCCNQTFSSTSGTATLLAALLNLNLSQLT GQIGLSCTPISVIGLGQGASCTQQPVCCSGN TYNGLINVGCSPINL	10.9	0.1	5.8	0.6	Secreted, cell wall	EDQ99414.1 [176]
**B0E2E5**	*Laccaria bicolor*	*LbH14*	PF01185	248	MLARLTASLYVVATMNIFAAASPQLGYYPPAS STVTTGSCTVATFTTTLAVTKPAIYTTTQTVTATP FTTTSTITIPLITTVTQPFTTTATTSIVSTLTVPITTT VTRPVTTTYTQQTTVGTVSTTSVTVPITTTIIKPV TITNTVTFIDPITTTVTLTATPSPSPSHQCNNGP ILCCDQVQEAGLLGAILGLLGIILSDVEGLLIG VTCVPLSTLGGNIGSKQPVCCDNDNYGGLV AIGCTPYPYAL	25.5	0.3	4.8	0.5	Secreted, cell wall	EDQ98979.1 [176]
**Q9P8T0**	*Lentinula edodes*	*hyd1*	PF01185	127	MLSLLSKAVSLAILVTAVVASPAGNGVEWSG GGTTTVTVTASSPTTTVTQSQCSTGDQKCCQ SVQNSSAAGVSSLLGLLGIVLSGTDVPVGLTC	12.4	0.2	4.1	0.6	Secreted, cell wall	AAF61065.1 [199]

Journal of Fungi

MDPI

					LPIVGGACQSQAVCCTDNSYGNLISLGCSPLQL						
**Q9P8S9**	*Lentinula edodes*	*hyd2*	PF01185	109	MQFKLAFVSIALATLAVATPAPRGEPASSCSTGDLQCCNTVEPASSPSASTILGLLGIVIQGVDVLVGLTCSPITVIGLESGGCSAQAVCCTDNSNGGLISIGCLPVTL	10.7	0.2	3.8	0.8	Secreted, cell wall	AAF61066.1 [199]
**Q4G2I6**	*Paxillus involutus*	*hydA*	PF01185	141	MKFTYVFALAAAATVVSAETNAQRMARGLPPKGPARRGTPSFGAKRGSPSGTPSGGSQCNTGPVQCCNSVQQCGSGNEVDTMLSLLGLSVPAGTNVGTNCSPISAVGTGSGASCTQQPVCCENNNFNGLVNIGCTPININL	14.1	0.2	8.3	0	Secreted, cell wall	AAX51848.1 [177]
**A9X617**	*Paxillus involutus*	*hydB*	PF01185	111	MFARLFTIASVAALALAGAIPATTPASQCNTGGLQCCNTVQSASVANAANPIWNLVSVVPGLAGNVGTDCTPITAIGLGSGSSCTAQPVCCTGNTFNGLVNVGCSPVNLGL	10.8	0.2	5.8	0.7	Secreted, cell wall	ABG33846.1 [216]
**A9X618**	*Paxillus involutus*	*hydC*	PF01185	107	MFSTSLLWIASVVALATADGHSQCTTGQIVCCNSLTSADAVPDLFSVTGLLATQAGLLGNVGVRCSTLTVIGEGKSCIASQQPLCCTDNNYNGLFSLGCSPINAMLV	10.9	0.4	4.2	0.6	Secreted, cell wall	ABG33847.1 [216]
**A9X619**	*Paxillus involutus*	*hydD*	PF01185	108	MFARVFALLPLALLASASHLEARDQCNTGSISCCNQVQSAQEASGLLSQLNLVALAAAAGGNVGLSCTPVTLIGTGSGCDATQQPVCCTGNQFNGLVNVGCTPINVNA	10.8	0.2	4.5	0.5	Secreted, cell wall	ABG33848.1 [216]
**A9X668**	*Paxillus involutus*	*hydE*	PF01185	107	MFTRVFTVVSVAALAIAGAAPTSQCNTGTTQCCNNVQSASSMNQLLSAVGFVNVLAGLTGNVGTDCTPITAVGTGSGADCTAQPVCCTDNTFNGLINIGCTPININA	10.6	0.1	3.8	0.5	Secreted, cell wall	ABG33849.1 [216]
**A9X669**	*Paxillus involutus*	*hydF*	PF01185	116	MFIRPSSIVLPIVALAAVVTAAPSALEARGSSQCNTGPIQCCSSTMTSSTTTLAELSSLLGLSLPSIGGLIGLSCSSITGVGTGTGAVCTQQPVCCSDNTYNGLINLGCSPININL	11.5	0.1	4.4	0.7	Secreted, cell wall	ABG33850.1 [216]
**A9X622**	*Paxillus involutus*	*hydG*	PF01185	108	MFARVFALLPLALLASASHLEARDQCNTGSISCCNQVQSAQQANQVLSQYNLVAAAAAAGGNVGLTCTPVTVIGSGSGCDATQQPVCCTGNQFNGLVNIGCTPINVNA	10.9	0.2	5.5	0.4	Secreted, cell wall	ABG33851.1 [216]
**Q2VTP0**	*Phlebiopsis gigantea*	*h1*	PF01185	108	MKFSVATLLALPLLAVATPLEVRQSGSCDTGSLQCCDSTITSTSASSNILLGLLGVVVGDLTGLV	10.6	0.3	4.1	0.9	Secreted, cell wall	AAY97884.1 [246]

					GLNCSPLSIIGIGSGNACTASPVCCSNTQVG GLIGVGCIPITL						
**Q2VTN9**	*Phlebiopsis gigantea*	*h2*	PF01185	153	MDICPVNSGHYGRGESSTASSCIPQSQIMKA QPPLSILALPLLAVATPLEVRQNGSCDTGSLQ CCTNTISSTSAQGNILLGLLGIVLSDVTGLLGL GCSPISVVGVGSGNACTASPVCCSNNSVGG LINIGCITHYPLIPSAVFWHVATEVDP	15.4	0.4	4.8	0.5	Secreted, cell wall	AAY97885. 1 [246]
**Q8X1T9**	*Pholiota nameko*	*pnh1*	PF01185	114	MQFTKMSAFATLALATLAAATPTRRNDSPSN QCNTGSLQCCNSLQSANSASLAGLLGLLGV VVGTITGQVGVTCSPITGVGVSGTSCSEQPVC CTNNAFNGVIALGCSPININL	11.3	0.1	7.6	0.5	Secreted, cell wall	BAB84545. 1 [205]
**Q8X1T8**	*Pholiota nameko*	*pnh2*	PF01185	110	MFARAASVFVLSLPILATANVLPRQDNQCNT GSLQCCSSVQSSSSSLVAILLGLLGVAAGGLT GQVGVTCSPITVIGVSGTSCSEQPVCCTGNT FNGVIATGCTPVNVNL	10.9	0.2	6.0	0.7	Secreted, cell wall	BAB84546. 1 [205]
**Q8X1T7**	*Pholiota nameko*	*pnh3*	PF01185	107	MQFKVLAALVIGATLAAATGSPSNQCNTGSL QCCNSTGGSATDPAIAKLFALLGINVEDVTALV GVTCSPITVVGASGSSCSEQPVCCTNDSFN GIVALGCAPINLNL	10.5	0.1	3.9	0.7	Secreted, cell wall	BAB84547. 1 [205]
**P52748**	*Pisolithus tinctorius*	*hydPt-1*	PF01185	140	MKFAAVVVLAAAAAAVSAETNAQRMARGLPP KAPIRRHGTPADTEKRSHPSSTGGGQCNTGP IQCCNTVATSGSQSGVDELLTLLGLSVPVGTQ VGASCSPISAVGTGSGAQCSGQTVCCEQNE WNGLVNIGCMPINLNA	14	0.1	7.7	0	Secreted, cell wall. Localizes at the surface of the hyphae with no preferential accumulation in the fungal cells of the different tissues of the	AAC49307. 1 [207]

										ectomyco rrhiza.	
**P52749**	*Pisolithus tinctorius*	*hydPt-2*	PF01185	117	EIVSLSLSLLAVVPLVVLVIAGPLNVRGGTPSQQCNTGTPQCCQQVQQTSDLQQFRSSFGLVDALAGASALVGANCNPVSVLGTGNGAQCNTQPVCCTSNQMLGAVNMGCMPLNVNA	11.8	0.2	4.4	0.4	Secreted, cell wall	AAC49308.1 [207]
**O93917**	*Pisolithus tinctorius*	*hydPt-3*	PF01185	108	MFSRVFAVASLAALALAGPLSVRDQCNTGTIQCCQQVQQASYYQSAFQEIGLGELLAGVTGQIGTQCSPISVVGASNGAQCNAQTVCCTNTQFNGLINIGCMPINVNA	11.1	0.2	4.4	0.4	Secreted, cell wall	AAC95356.1 [207]
**Q9Y7G9**	*Pleurotus ostreatus*	*vmh1*	PF01185	116	MLFKQAILVATTLTDLAVATPVVDVRRRTDPASSCSTGTLNCCNSSGTVEDKTIAGLLGILNIVVSDITALVGITCTPITVVGAGGTSCTSQTLCCDNNNFSGLITLGCIPININL	11.8	0.1	4.4	0.7	Secreted, cell wall	CAB41405.1 [178]
**A0A067N4H9**	*Pleurotus ostreatus*	*vmh2*	PF01185	111	MFSRVMFCTFLILPLLAAATAIPRTDTPSCSTGSLQCCSSVQKASDPLVGIIVALLGIVLGPLDLNVGLTCSPITVIGVGGTSCTQQTVCCTGNNFDGLIVAGCSPINIGL	11.2	0.2	4.5	0.9	Secreted, cell wall	KDQ22759.1 [179]
**A0A067N648**	*Pleurotus ostreatus*	*vmh3*	PF01185	108	MFFQTTIVAALASLAVATPLALRTDSRCNTESVKCCNKSEDAETFKKSASAALIPIKIGDITGEVYSECSPIVGLIGGSSCSAQTVCCDNAKFNGLVNIGCTPINVAL	11.2	0.2	4.9	0.4	Secreted, cell wall	KDQ23304.1 [179]
**O13502**	*Pleurotus ostreatus*	*POH1*	PF01185	113	MFSIRISTVVLAASALLAVAIPMTNTETPQCNTGPIQCCNSVQSATSSAAAGPLAALGVLSGIASLLGEVGLDCSPLQVIGVGANSCSSQAACCTGNTFNGAVVLGCSPIKLL	11	0.1	4.4	0.8	Secreted, cell wall	CAA12391.1 [230]
**O13503**	*Pleurotus ostreatus*	*POH2*	PF01185	131	MFFRTSSLFTTIVAFTVMAAAMPGNPKPTTTTVTVTAPAHPTATAPASECKTGPVQCCNSVQSSKSPAASLLLGLLGIVLQGVAVPVGLTCNPITVIGVGGNSCSAQTVCCENNNFSGLIAIGCTPINLSL	13.1	0.1	8.0	0.6	Secreted, cell wall	CAA12392.1 [230]
**O60048**	*Pleurotus ostreatus*	*POH3*	PF01185	113	MFSRVIFCTFLILPLLAAATAIPRTNPPAPTCTTGSLQCCNSVQAASNPVVGLLAGLLGIVLGPITGQVGLTCSPITVIGVGGTSCSAQTVCCNGNSFNGLIVVGCSPVNISL	11.2	0.2	7.9	1	Secreted, cell wall	CAA76494.1 [230]
**O60047**	*Pleurotus ostreatus*	*fbh1*	PF01185	113	MFSIRIATVVLAASAALRPPARITNTETPVNQCGSGSIQCCESVQSASAAQAAGILGPLDILTNL	11.2	0.1	7.6	0.5	Secreted, cell wall	CAA06192.1 [232]

Journal of Fungi

MDPI

					QGLVGSHCSPLAAVGVSGTSCSSQTVCCKDVSKSGLVNLGCSPINLNL						
**A0A067N582**	*Pleurotus ostreatus*	*Hydph5*	PF01185	127	MFNKQTNAIVLLFTFALFATLAVATPTIVARTGGSGSSCPTTDIKCCSHVGTFSEVQGYIDDDTKVKLIPALLGLLGATIDVVLGVVLGVTCSSISVGSSCSATTVCCENVVFNGLINVGCTAIDIL	13	0.2	4.5	0.9	Secreted, cell wall	KDQ22994.1 [179]
**A0A067PCX8**	*Pleurotus ostreatus*	*Hydph6*	PF01185	114	MLFKQLILVATALTTLAVATPAVMRRTEPASSCSTGPVNCCNSSGTAKDGNIAKELALLGIVVPDINALIGVSCSPITVIGAGGASCSSQTLCCEDNKYNGIVALGCIPVDISL	11.5	0.1	*4.8*	0.7	Secreted, cell wall	KDQ34252.1 [179]
**A0A067P1E6**	*Pleurotus ostreatus*	*Hydph7*	PF01185	109	MFAQSFIITALAALAVASPLQVRTSDKCNTGTVHCCNSMKKSDSADISKIASLLQLDVKGVVGDVGLQCSPLVSLVGGGSKCSGQTVCCDQTKFNGLVNIGCSPINVGL	11.1	0.1	8.0	0.4	Secreted, cell wall	KDQ30217.1 [179]
**A0A067NFU6**	*Pleurotus ostreatus*	*Hydph8*	PF01185	125	MMFSKPVVLATTALATFAAATPLVARTEPTCSSTEIKCCENVGTAAVVSSVLTPILVSIPLVGPLLSLLGLNAVVALLFGTVDVVLGLTCSGIDVGGSCNAQTVCCENVQFNGLINVGCVAIDIL	12.6	0.1	4.0	1.1	Secreted, cell wall	KDQ22992.1 [179]
**A0A067N4P7**	*Pleurotus ostreatus*	*Hydph9*	PF01185	123	MFFFNTKPIVFLVVLSVVATFAAATPAMLARGGGSSCAPSTSLKCCDHVGTFSEVSPYINPLELVGVIAALLGLVGGLFTSVTLALTCSGIDIGGSCNSQTVCCENVVFNGLVNVGCTAIDIL	12.5	0.1	4.4	1	Secreted, cell wall	KDQ22993.1 [179]
**A0A067NN74**	*Pleurotus ostreatus*	*Hydph10*	PF01185	111	MLFNTFVVTALASLAAATPTRRNEPAAGSCSTGELQCCKSVQSADSKSLTSLFGLLGLVVGDITGLVGVTCSPVTVVGAGGAQCNAQAVCCNDNSFNGLIALGCTPINLNL	11.1	0.2	4.6	0.6	Secreted, cell wall	KDQ29538.1 [179]
**A0A067P0I9**	*Pleurotus ostreatus*	*Hydph11*	PF01185	129	MRLTPLLAALALPLLTVLATPLTPVGEAEVVEVDVAPRQISLNPASSCPKGQLWCCVSLDLVSNSLIAGLLVTLGILDPTGINTAGVQCTPLSWPFQMSCGNKDLCCDPDLPVVGLLLKLNCRSVNPLL	13.5	0.2	4.3	0.7	Secreted, cell wall	KDQ29912.1 [179]
**A0A067N4P3**	*Pleurotus ostreatus*	*Hydph12*	PF01185	104	MFSKATLFFTAAVVIVAAGATPTTSQCNTGPVQCCDHTQQASGPTTLTGIGEVDLGDTSTLIAYGNCSPVLPVLGGPKCQGQTVCCDNTEFEGLVNVGCTNVAV	10.5	0.1	3.9	0.3	Secreted, cell wall	KDQ22804.1 [179]
**A0A067P494**	*Pleurotus ostreatus*	*Hydph13*	PF01185	95	PTKMTRRTEPASSCSTGSLDCCNSSGDATDTSITTLLAGIGLPIGSVTGLVGVTCSPITGIGLGGSGCSSEALCCSNNSFKGLVALGCVPVDLSL	9.2	0.1	4.3	0.4	Secreted, cell wall	KDQ34065.1 [179]

**A0A067NRQ5**	*Pleurotus ostreatus*	*Hydph14*	PF01185	133	MFSNAAAFSTVALATYAAATPAVVARTEPHPSSSNCGTTTIECCKDVGTATSLESPLKSAFIQHGLVSSLLVALGVVKVVDVVLGVVVDPNVVLGLTCSTVAVGGSCNAQQVCCENVAFNGLVNVGCTAIDIL	13.3	0.1	4.5	0.8	Secreted, cell wall	KDQ26291.1 [179]
**A0A067NHW8**	*Pleurotus ostreatus*	*Hydph15*	PF01185	135	MFAKSATIAIVLAALAGFSAAVPTADCRTSKTCSSGKVALVAPAAPVGSCSTGKVQCCNSVEHSTQPHVNNLLLGLEHFGLVKGLIGGLTGNVGIQCSPLLLSGNSCTAQTACCENVHFNGLIAVGCSPVNLSLL	13.5	0.1	7.9	0.6	Secreted, cell wall	KDQ23321.1 [179]
**A0A067P943**	*Pleurotus ostreatus*	*Hydph16*	PF01185	136	MKFTSVIALVATAATLVGAVPFETNAERLARGLPPLPPARRASGVEAAKPKPSPSHGCSTGPVQCCNDLVDRSNHTVGILIGLLGIVLGPVTGLFGLGCSPLLGGGAKCQSQTVCCSDNKFSGIINIGCSPINIGL	13.7	0.2	8.4	0.4	Secreted, cell wall	KDQ32927.1 [179]
**A0A067NHE1**	*Pleurotus ostreatus*	*Hydph17*	PF01185	135	MFAKSATIAIVLAALAGFSAAVPTADCRTSKTCSSGKVALVAPAAPVGSCSTGKVQCCNSVEHSTQPHVNNLLLGLEHFGLVKGLIGGLTGNVGIQCSPLLLSGNSCTAQTACCENVHFNGLIAVGCSPVNLSLL	13.5	0.1	7.9	0.6	Secreted, cell wall	KDQ23321.1 [179]
**A0A067NEJ5**	*Pleurotus ostreatus*	*Hydph18*	PF01185	109	MFTEQVLNVIILLQTATVTATAIPASGCNAGDVQCCKSVQKADSAGGAALLGLVGIVLSNLDVFIGGDCSPITAIGVGGTSCSSQAVCCENNSFNGLIALGCVPINLSL	10.8	0.1	3.8	0.8	Secreted, cell wall	KDQ26264.1 [179]
**A0A067NRJ8**	*Pleurotus ostreatus*	*Hydph19*	PF01185	128	MILLAFSGELFKRINTSPIVHVERRTNPTTTVTVTTKPSPTPISPNQCNTNKIQCCNSVQSSDAPLLTGLLGLLGIVLQGVIPIGVTCSPISVIGIGGNSCSAQTVCCENNTFNGVIAIGCTPINIDL	13.3	0.2	7.6	0.4	Secreted, cell wall	KDQ26241.1 [179]
**A0A0677P3**	*Pleurotus ostreatus*	*Hydph20*	PF01185	143	MLSHPMKLLFFVFALSALLAAATPVVEVRDYQQFSRVSLINFISFNKARGGDLCANGKKTMCCGELKTFDKNILGLLDGLLTTVVGLAGEILKPTGLVGLECVPITLLGNGGLSDVCKANGVCCKDLTLRAAINVGCVPINIL	15.1	0.2	8.2	0.7	Secreted, cell wall	KDQ22990.1 [179]
**A0A067NMT6**	*Pleurotus ostreatus*	*Hydph21*	PF01185	115	MFAAPATMLVLAALAALSSAIPAAQTASKCSTGPVQCCDSVEHHTQPHVNNLLLGLEHFGLVKGLVGGLTGNVGIKCNPILLSSNDCTAQSVCCEHVHFKGNIAVGCTPANIDIL	11.7	0.2	6.8	0.5	Secreted, cell wall	KDQ25427.1 [179]

Journal of Fungi

MDPI

**Q8X1N0**	*Pleurotus ostreatus*	*Po.hyd*	PF01185	110	MFSKATLFFTTVSRYRDTQAPIPTGQTNQPTTNQCNTGPVQCCDTTQSASDPINIPGMGLVHVSNANGLVAFGNCSPLVSGGSKCNNQAVCCNGTEFNGLVNVGCTNVSL	11.4	0.4	7.0	-0.1	Secreted, cell wall	AAL57034.1 [180]
**P04158**	*Schizophyllum commune*	*SC1*	PF01185	109	MRFSLAILALPVLAAATAVPRGGASKCNSGPVQCCNTLVDTKDKHQTNIVGALLGLDLGSLTGLAGVNCSPVSVIGVGGNSCSTQTVCCEGTQFNGLVNVGCTPINVGL	10.8	0.3	7.6	0.5	Secreted, cell wall	CAA25366.1 [181]
**P16933**	*Schizophyllum commune*	*SC3*	PF01185	136	MFARLPVVFLYAFVAFGALVAALPGGHPGTTTPPVTTTVTVTTPPSTTTIAAGGTCTTGSLSCCNQVQSASSSPVTALLGLLGIVLSDLNVLVGISCSPLTVIGVGGSGCSAQTVCCENTQFNGLINIGCTPINIL	13.4	0.1	5.4	0.8	Secreted, cell wall. Localizes in cell wall of aerial hyphae and is abundantly secreted from the tips of submerged hyphae.	AAA96324.1 [182]
**P16934**	*Schizophyllum commune*	*SC4*	PF01185	111	MRFSLALLALPALAAAAPVPGGGKGAGQACNSGPVQCCNETTTVANAQKQGLLGGLLGVVVGPITGLVGLNCSPISVVGVLTGNSCTAQTVCCDHVTQNGLVNVGCTPISL	10.7	0.3	7.6	0.7	Secreted, cell wall	AAA33927.1 [182]
**O74300**	*Schizophyllum commune*	*SC6*	PF01185	185	MVSRVLALISVAMLVGARPYVQNVGDVDLNDVDATVGTGVVDTGMSGDLLAGLLANGLLADLLSEDADGHIPEVTGSSTEEATSSSTWSGASSKPTDSAPTQCNSGTLQCCESTTEAKDIDRVLLSTLLGVDVGSITGLIGKNCSPVSVVGVGAGSTCSTQTVCCDGDSFDGLINLGCKSGNVAV	18.4	0.2	3.7	0.3	Secreted, cell wall	CAA07545.1
**D8QCG9**	*Schizophyllum commune*	*SC16*	PF01185	114	MRFFATLVLALPALAMATAVPRDVNGGTPPKSCSSGPVYCCNKTEDHLDKGTTALLGLLNIKIGDLKDLVGLNCSPLSVIGVGGNSCSAQTVCCTNTYQHGLVNVGCTPINIGL	11.7	0.7	7.7	0.4	Secreted, cell wall	EFI94929.1 [183]

**D8Q774**	*Schizophyllum commune*	*HYD2*	PF01185	122	MAVLARVYTMFFFIFAVLPCLAAAAAVVPRGGGGNDSGQHCSSGPVQCCQTSQSADSQGAAGLLGLLGIVLQDVTALIGLSCTDVNVLGGGGSECSAQTLCCENNHFDGLINLGCSPINIYL	12.3	0.2	4.1	0.6	Secreted, cell wall	EFI96361.1 [183]
**D8PTH5**	*Schizophyllum commune*	*HYD3*	PF01185	113	MRFTIALFAALPLLVAASAVPRAPGGQKCRAESVQCCNTTYSHKDYSHVEYLQGLGISPEVLANPDASSYADCTPLSVVGGHGCQASSTAVCCENNYQRGAVNMGCVPISMAA	11.7	0.3	6.7	0.2	Secreted, cell wall	EFJ01319.1 [183]
**D8PU87**	*Schizophyllum commune*	*HYD4*	PF01185	109	MRFTLAAVLALPLLVSAAAPGNNKCNTGEVQCCNQVTTPKDAAQSDVTAGLIGALIGPITGPLVGLHCSPLSLVGVGGACQASAQAVCCENVHETGLVNVGCTPIDLKA	10.8	0.2	5.5	0.6	Secreted, cell wall	EFJ00584.1 [183]
**D8QC97**	*Schizophyllum commune*	*HYD5*	PF01185	90	MRVVALSALFASSLLGVGALPSASGCSAEGIWCCNENAIRTDISGLGKGCTQTSIIGIGGTRCQGQTVCCDKTDQNGILNAGCKPTILGV	9	0.2	7.6	0.4	Secreted, cell wall	EFI94892.1 [183]
**D8Q897**	*Schizophyllum commune*	*HYD6*	PF01185	151	MKLVTFFAFLFALLIAVSAVPTAETNADRMRRGLTPMRPRNLGRATPVDSASKHKPSGGGGNGQSCSSGPVQCCQSVQKSNSGSLLGIILKLLGIVLGEVTDIGLHCTPIDVLGGGGNQCFPSSAQTVCCKDNSYSGHGINIGCSPINIGL	15.5	0.2	8.6	0.2	Secreted, cell wall	EFI96155.1 [183]
**D8Q7C2**	*Schizophyllum commune*	*HYD7*	PF01185	116	MRFFATLVLALPALAMATAVPRDVNGGTPPKSCSSGPVYCCNKTEDSKHLDKGTTALLGLLNIKVGDLKNLVGLNCSPISVIGVGGNSCSAQTVCCTNTYQHGLVNVGCTPINIGL	11.9	0.7	8.2	0.3	Secreted, cell wall	EFI96392.1 [183]
**D8PVA0**	*Schizophyllum commune*	*HYD8*	PF01185	279	MFARIFTIVSVAMLVAAQPHGRGLPVLGGILGGGGGGGGATVTVTVPGECPTVTQTVTVTGSGTWGVPTSSSTWGVPTSSSTWGVPSSSSSSVHSSSTSKSDEHTSSSSVPTWGPTGTPTWTSKTEDPTSKTSTTSHSSVNTSTWGTSKTDTTSATWGTGSSSVPSSSSTSKDTGSTTWTSEPTKPTDPGNGGGQCNSGTVKCCNSVKKVKDVGSAGLIAGLLAIGGLNLDSLVGVQCSPLSVIGVGGNSCSSQVACCSNNTYNGLINIGCVPISISL	27.4	0.2	7.7	-0.1	Secreted, cell wall	EFI99995.1 [183]
**D8Q8Y7**	*Schizophyllum commune*	*HYD9*	PF01185	103	MRFSILVFALPALVAATGTPASCSGGGVYCCNKTVDHNDKSIQKQLSLVGVTAGSIHDLGLTCTPITAIGVGSGAQCSAQSVCCSKSYQNGLVNVNCTPIKIL	10.4	0.2	8.2	0.5	Secreted, cell wall	EFI95556.1 [183]

**F8NJA2**	*Serpula lacrymans*	*slh4*	PF01185	104	MFAKVVLVFTALAAVVAAGSSQCNAGPVQCCNTLTSASNSQAAGLIQQLGLSGVGANVPVGINCNPITGIGAGSGSSCNANPACCDNVYTNGLGVQCNPINVNL	10.1	0.3	5.8	0.5	Secreted, cell wall	EGO29586.1 [184]
**E6ZQB8**	*Sporisorium reilianum*	*Hum2*	PF01185	117	MQFKTIFAAVATLAAVASALPTASEHNGNGQCAVGQAQCCSQIQKQGKQAQDALAGLGLAFNEVLDGAIGLDCQQVPVGVLGVAVGVQNTCKNTAVCCQGSANNGLIQTSCTPLSIN	11.7	0.4	5.6	0.3	Secreted, cell wall	CBQ69425.1 [185]
**E6ZPU4**	*Sporisorium reilianum*	*Hum3*	PF01185	789	MKYLQFLAAVAAVSAFSGPVLAGSVVDNTNQVAPVQLTGALLSQVANGQHAERAVKVDNSAQFFPIEGTAAALSAVLNQQKATKRWEKEGKVVDNTNQVLPIQATLAALCQVLNEQKAAKRDNVVKNDNQIVPIEATLAALSAVANGQKSHARSVKVDNSAQFVPIQGTIAALSQVLNEQKATKRDVVDNTNQVLPVEATLSALSQVLNSQKAERSVDVHNSAQFLPIEATLAALSAVANAQKAEKRKAPEFDSVENTNQVAPIQATAALLSQVANLQAAHSKRWEKDGYVVENTNQVLPIQATAALLSQVANLQSATKRWEHPAHVVDNSNQVLPVQGTLAGLSQVANGQLVSRKAPEFDSVKNSNQVAPIQATAALLSQVINKQTVEKRKAPEFHSVKNTNQVAPIQGTAAAFSQVLNEQIASRKAPEFDNVVENTNQVLPIQATAALLSQVINKQAATKRHEGENNIVENTNQVLPIEATLAALSSVLNAQKAERSVDVDNSSQFAPIEATLAALSQVANGQHATKRGEAVINDNQVAPVEAVAAALSQVVSGQKTRRGETKEIHHVNQVAPAQATLTALSDIVNSAHSRRAMDVKQYETLQYAIDALQQALDKTNTGDATHHVDALTGSAEHITPKRDSGLDPAAFLGSATSGISVPVGNKDLLKSLVKRYAEEEDAAMAKRTPSPHGHGQRPRPRGASEHNNNGQCSVGKAQCCNQVIHDESKKKTLAGLLGFNNLAGSIGLNCQQIPVLGVSLQSVCKSTPVCCTNVVQDGLVNIGCTSIPIN	83.5	0.4	8.0	-0.2	Secreted, cell wall	CBQ69251.1 [185]
**A0A0C9ZLX1**	*Suillus luteus*	*HydSl1*	PF01185	136	MKFASVLAVAVAATVVSAETNAERMVRGLPPLAPARRGTPVARAKRTSPSSGSGQCNTGSIQCCESVSKSGHGNVLDELLSVLNIDIPFDTSC	13.8	0.1	7.6	0.1	Secreted, cell wall	KIK38525.1

					GVSCSPISVVGGGSGAKCNQEPVCCENNSYNGLVNIGCSPINI						
**A0A0D0AYW4**	*Suillus luteus*	*HydSl3*	PF01185	110	MFVRILSITSLAVLAIASPLALRADGQCDTGNIQCCQSTQPVDAYNNNATMYGEIPILIDILGDVGLGCSPISVIGTGNGANCLQQPVCCSNESYSGLINIGCTPIDLSL	11.4	0.1	3.6	0.5	Secreted, cell wall	KIK42959.1
**A0A0D0BGG3**	*Suillus luteus*	*HydSl4*	PF01185	109	MFARFFAVASLAALAVATPLSLRDGQCNTGSIQCCQHSTSTSNYNKAAEILGLVPIGADVAGSVGLSCSPISVVGTGSGASCNQQPMCCSNNKMNGLVNVGCTPINLSL	11	0.1	7.6	0.4	Secreted, cell wall	KIK42333.1
**A0A0D0AM44**	*Suillus luteus*	*HydSl5*	PF01185	109	MFARFFTVVFLAALAVANPLSLRDGQCNTGDIQCCQSSTTTSTYNKAAKSLGLVEIAAGVVGQVGLACSPVSVVGTGSGASCNQQPMCCSNNKMNGLVNIGCTPINLKL	11.1	0.1	8.2	0.4	Secreted, cell wall	KIK39179.1
**A0A0D0B5N3**	*Suillus luteus*	*HydSl6*	PF01185	121	MFALSLLLVSLFAFIVGSSAVPTLSARDNSTCSSSGAQCNTGTVSCCQSTYASNSNSLSRITSALNIALDPVEGSVGLGCSPISVIGTGSGAVCNQEPVCCSGNTYYGLINIGCSPINIYL	12.2	0.1	4.1	0.6	Secreted, cell wall	KIK39158.1
**A0A0D0ALE0**	*Suillus luteus*	*HydSl7*	PF01185	103	MFARIFAVASLVAFALADGQCNTGNIQCCEGSTTVSKYNDASGILGLAAIAADVTALVGLNCSPVSVVGTGAGCEANQEPLCCSNNKYNGLVNIGCTPINVIL	10.3	0.2	4.1	0.6	Secreted, cell wall	KIK38939.1
**A0A0D0AMA6**	*Suillus luteus*	*HydSl8*	PF01185	103	MIFSIFALLPFALFAAAQSQCDTGTIQCCEKDSMYSQTEPTLTELGLVDVAAVVDAFVGLSCSGINVVGTSTGCTANQEPVCCENEKYNGVASIGCTPINVDL	10.7	0.2	3.5	0.5	Secreted, cell wall	KIK42956.1
**A0A0C9ZII0**	*Suillus luteus*	*HydSl9*	PF01185	106	MFASILALLPFALLAVADSGQCNTGEISCCNNSTSYDSNAAQKAFEQVGLVDVAAVVDAFVGLECSGISVVGTSSGCEANQEPLCCEDNSYNGLVNLGCTPINVVL	10.8	0.2	3.3	0.5	Secreted, cell wall	KIK37230.1
**A0A0D0AZF5**	*Suillus luteus*	*HydSl10*	PF01185	105	MFASILALLPFALLAVADGQCNTGEIQCCNNSTSYNSYAAQKAFNQTGLVDVVAVVNAVVGLDCSGINVVGTSDGCDANQEPLCCEDNKYNGVVSLGCTPINVKL	10.8	0.2	3.8	0.4	Secreted, cell wall	KIK37233.1
**Q8TFP0**	*Tricholoma terreum*	*Hyd1*	PF01185	108	MFSKVALIVATLAAFVAATPLPGGSSQCNTGSIQCCQQTYSSTSSEASLIASIVGLDLSGVTGSIGSQCSPISAIGLGSGSSCTQQPVCCSNNNYQGLIVVGCSPINL	10.7	0.1	4.2	0.5	Secreted, cell wall	AAL05426.1 [238]

**A0A0D1B ZL3**	*Ustilago maydis*	*Hum2*	PF01185	118	MQFKTIFATLAAFAAVASALPTSSSEQNGNG QCAVGQAQCCSQVQKQGKDASDALAGLGL AFNQILDGAIGLDCQQIPVGVLGGAIAIQNTC KNTAVCCQGSANNGLIQTSCTPLSIN	11.8	0.4	4.6	0.3	Secreted, cell wall	KIS67142.1 [3]
**A0A0D1C KI0**	*Ustilago maydis*	*Hum3*	PF01185	828	MKYLQFLAAVAAVSAFSGPVLAGSSVENTNQ VLPVQLTGALFSQVANGQHAERAIKVDNSAQ FVPVQGTIAALSAVLNEQKAGKRSFSVENTN QVLPIDVIIAGLSQVLTEQKAAKRDNIVKNTN QILPIEATIAALSAIANGQKAATKRSTAVDNSS QFIPIQGTLAAFSNVLNSQKATKRNTGKVVD NTNQVVPIQGTLAALSTVLNEQKASKRGVDV DNSSQTLPIEATIAALSTIANGQQAGKRNAPD FDVVKNSNQVLPIQATAALLSQIANGQSVEK RNAPDFDVVKNSNQVLPIQATAALLSQIANG QSVEKRNAPDFDVVKNSNQVLPIQATAALLS QIANGQSVEKRNAPDFDVVKNSNQVLPIQAT AALLSQIANGQSVEKRNAPDFDSVKNSNQV VPIQATAALLSAVKNLQSVERNAPDFDSVKN SNQVVPIQATAALLSQIANGQSVEKRNAPDF DSVKNSNQVVPIQGTAALLSVVGNGQSVSK RHEGENNIVDNTNQVIPIQATIAALSTLLNSQ KAERSYSVDNTNQVLPIEATLAAFSSVLNSQK AERSYSVENDNEVVPVDLVAAALSQVANGQ KVTRRGETKESIKHNVNQVAPAQASLSALSDI VNSAHSRRAMDVKQYETLQYAINALQQALDK TNTGDKTHHVDAMTGTAEHFDERDAGLDPA AFLGSAASGISVPVGNKDLLKSLVKRYAAEED EAMAKRTPSLNGRGQRPRPRNSSSSEHNN NGQCSVGEAKCCSQVITDEGKKKTLAGLLGF NNLVGDIGLNCQQIPVLGVSLQSICKATPVC CTNVSQDGLVNVGCTSIPIN	87.2	0.5	8.2	-0.2	Secreted, cell wall	KIS67333.1 [3]

References

1. Edgar, R. C. MUSCLE: multiple sequence alignment with high accuracy and high throughput. Nucleic Acids Research, 2004, 32(5), 1792-1797, doi:10.1093/nar/gkh340.

2. Kumar, S., Stecher, G., Suleski, M., Sanderford, M., Sharma, S., & Tamura, K. MEGA12: Molecular Evolutionary Genetics Analysis Version 12 for adaptive and green computing. Molecular Biology and Evolution, 2024, 41(1), 1-9, doi:10.1093/molbev/msae263.

3. Jones, D. T., Taylor, W. R., & Thornton, J. M. The rapid generation of mutation data matrices from protein sequences. Computer Applications in the Biosciences, 1992, 8(3), 275-282, doi:10.1093/bioinformatics/8.3.275.

4. Saitou, N., & Nei, M. The neighbor-joining method: A new method for reconstructing phylogenetic trees. Molecular Biology and Evolution, 1987, 4(4), 406-425, doi:10.1093/oxfordjournals.molbev.a040454.

5. Nei, M., & Kumar, S. Molecular Evolution and Phylogenetics. Oxford University Press, New York, 2000.

6. Letunic, I., & Bork, P. Interactive Tree Of Life (iTOL) v5: an online tool for phylogenetic tree display and annotation. Nucleic Acids Research, 2021, 49(W1), W293-W296, doi:10.1093/nar/gkab301.
